# Point Defect-Mediated Electronic Tuning and Thermal Stability of Carbon-Doped Boron-Nitride Fullerenes for Radical Scavenging

**DOI:** 10.1021/acsomega.6c05661

**Published:** 2026-10-21

**Authors:** Gabriel García-Laiton, Edith Corona-Jiménez, Kerry J. Wrighton Araneda, Ernesto Chigo Anota

**Affiliations:** † Benemérita Universidad Autónoma de Puebla, Facultad de Ingeniería Química, 3972 Ciudad Universitaria, San Manuel, Puebla 72570, Mexico; ‡ Escuela de Kinesiología, Facultad de Ciencias de la Salud, Centro Integrativo de Biología y Química Aplicada (CIBQA), 28042 Universidad Bernardo O’Higgins, General Gana 1702, Santiago 8370854, Chile

## Abstract

Reactive oxygen and nitrogen species (ROS/RNS) are involved in redox biology, but they remain challenging to control due to their high reactivity. This research evaluates the point defect-mediated electronic tuning of boron nitride fullerenes (BNFs) via progressive carbon (C) doping to enhance the scavenging of nitric oxide (NO) and superoxide anion (O_2_^–^). High-level density functional theory (DFT) calculations (ωB97X-D/6-311++G­(d,p)) in gas and aqueous phases demonstrate that C-doping induces a transition from weak physisorption in pristine B_12_N_12_ to strong covalent chemisorption in C-doped systems when NO is adsorbed. Calculated thermodynamic functions support favorable adsorption of strongly bound complexes within the modeled temperature range. Pristine B_12_N_12_–NO remains unfavorable at 298 K. Quantum Theory of Atoms in Molecules, Electron Localization Function, and Localized Orbital Locator analyses reveal that localized spin density at the C-defect sites facilitates the formation of stable C–N and bridging C–O/B–O bonds. Finite-temperature stability was assessed using 300 K atomic density matrix propagation (ADMP) trajectories together with potential energy surface (PES)-based Langevin first-passage and mean first-passage time (MFPT) analyses. Adsorption produces system-dependent spectral redistribution, including selected near-ultraviolet/visible features and charge-transfer transitions. These results identify C-doped BNFs as theoretical metal-free radical-trapping platforms, while emphasizing the necessity of experimental validation before proposing biomedical or sensing applications.

## Graphical abstract

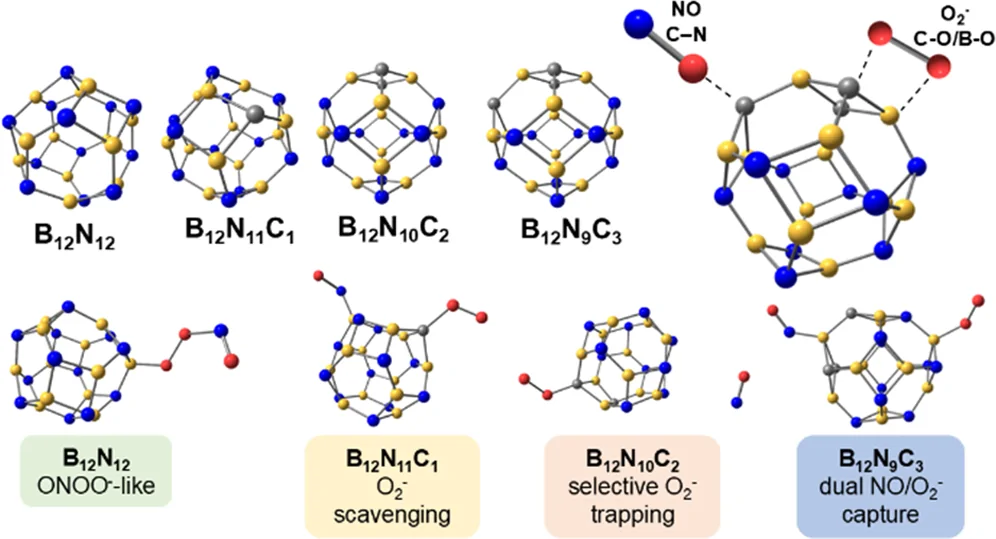

## 1 Introduction

Nitric oxide (NO) and superoxide ion (O_2_^–^) are reactive oxygen and nitrogen species (ROS/RNS) involved in redox biology. NO is synthesized in blood and vascular tissues and is implicated in cellular contraction, messenger activity, and relaxation phenomena.^1^ The high reactivity and rapid diffusion of these molecules out of cells lead to a variety of intracellular and transcellular processes, including neurotransmitter release, nervous system function, synaptic plasticity, and modulation of gene expression.^2^ As a signaling molecule, O_2_^–^ regulates various physiological functions, including vascular homeostasis, immune response, and cell proliferation^3^ and its production increases in conditions of oxidative stress or cellular imbalance. Both NO and O_2_^–^ are pivotal molecules in a wide range of cellular processes, and alterations in their signaling are associated with diseases such as Alzheimer’s, Parkinson’s and amyotrophic lateral sclerosis (ALS).

Peroxynitrite (ONOO^–^) is a highly cytotoxic substance produced by the combination of NO and O_2_^–^. ONOO^–^ is involved in a variety of biological processes, including apoptosis, protein oxidation, and lipid peroxidation.^4^ This reaction is notably rapid and diffusion-controlled, implying that the rate of ONOO^–^ formation is conditional upon the concentrations of NO and O_2_^–^ within the body. Studies on cellular toxicity show that an excess of calcium ions (Ca^2+^) can stimulate neuronal nitric oxide synthase (nNOS) activity, resulting in elevated NO levels.^5^ In vascular environments, the accumulation of ONOO^–^ can compromise endothelial NOS (eNOS) function, leading to pathway uncoupling and subsequent cell damage. Experimental ROS/RNS sensing has been performed using complementary optical and electrochemical platforms. NO has been monitored in biological environments using near-infrared fluorescent single-walled carbon (C) nanotubes; hemin-functionalized graphene field-effect transistors; C-dot colorimetric/fluorescent probes; flexible Ni–N_4_ single-atom electrochemical sensors; and biodegradable wireless electrodes.^6–10^ O_2_^–^ release from living cells has been quantified using O_2_^–^ dismutase-functionalized C nanotube/ionic liquid composites, cytochrome *c*/nanoporous gold electrodes, nonenzymatic Ag NP/MWCNT sensors, and graphene/DNA/Mn_3_(PO_4_)_2_ biomimetic electrodes.^11–14^ Dual-reactivity surface-enhanced Raman scattering (SERS) nanosensors have also enabled the simultaneous intracellular detection of NO and ONOO^–^.^15^ These studies demonstrate the experimental feasibility of ROS/RNS monitoring, illustrating that many high-performance platforms rely on transition-metal centers, metal nanoparticles, immobilized enzymes, or irreversible probe chemistry. C-doped BNFs represent a complementary, metal-free design concept in which molecular recognition and optical response are modulated by C-induced defect states and adsorption-mediated charge transfer. Nonetheless, the present calculations establish only theoretical sensing and radical-capture potential. Experimental selectivity, regeneration, aqueous stability, and biocompatibility must still be demonstrated.

Despite the exploration of nanomaterials for ROS/RNS sensing and scavenging, current chemical sensors face limitations such as restricted selectivity, instability in aqueous media,^16^ and a lack of adsorption reversibility.^17,18^ Systems capable of capturing, stabilizing, or modulating the behavior of ROS/RNS are of therapeutic interest, extending toward indirect functional evaluation. In this context, boron-nitride-based nanomaterials, owing to their biocompatibility, chemical stability, and special electronic properties, are well-suited for the selective adsorption of biomolecules, as semiconductor materials,^19^ and drug transporters.^20^ Boron nitride fullerenes (BNFs) exhibit distinctive electronic, mechanical, and chemical properties, positioning them as practical candidates for applications in nanoscience.^21^ The ability of BNFs to interact with biomolecules through hydrogen bonds and van der Waals (vdW) forces is ideal for applications in biosensing. Their high thermal and chemical stability, low-toxicity, and insulating behavior have led to their growing exploration in environmental, biomedical, and catalytic fields.^22,23^ BNFs are structurally analogous to carbon fullerenes (CFs) but composed of alternating boron (B) and nitrogen (N) atoms and exhibit distinct allotropes, such as B_12_N_12_ and B_24_N_24_, with unique electronic distributions and B–N bonding patterns that confer specific reactivities.^24,25^

The unreactive nature of pristine BNFs toward these radical species, however, limits their direct application as efficient sensors or scavengers. One effective strategy for overcoming this inertness is the use of C-doping, which serves as a highly effective electronic activation method. Research has shown that C-doped BNFs exhibit increased reactivity toward small reactive molecules, such as NO. Notable changes in adsorption energy, electronic structure, and magnetic properties were observed upon doping.^26–29^ Their nonmetallic nature, in connection with the capacity to undergo electronically modified states through doping or functionalization, positions them as practical candidates for the development of biocompatible and environmentally sustainable materials. C-doped BNFs were selected for their ability to modify electron density, reduce the HOMO–LUMO gap (HL_gap_), and create active sites through the doping of C atoms,^30^ enhancing their interaction with radicals compared to their pristine structure. Titanium^31^ and transition metals^32^ have been shown to enhance the adsorptive strength of nanomaterials. Their application in biomedical contexts is more limited due to concerns related to redox activity and potential toxicity.^33^ These nanomaterials have potential for therapeutic delivery, catalysis, or redox sensing for species like NO or O_2_^–^.

These advancements in nanomaterial design are notable, but there is still a lack of comprehensive studies integrating adsorption mechanisms, dynamic thermal stability, desorption kinetics, and optical responses for the specific capture of ROS/RNS using C-doped BNFs. Evaluating these functional properties in a combined analysis is essential to define their potential as candidate radical-capture and sensing platforms in complex biological or aqueous environments.

To address this scientific gap, this study investigates the selective capture of NO and O_2_^–^ on pristine B_12_N_12_ and progressively C-doped BNFs (B_12_N_11_C_1_, B_12_N_10_C_2_, B_12_N_9_C_3_, and C_24_). Simultaneously evaluating NO and O_2_^–^ enables testing whether C-doped BNFs can capture both species, selectively remove O_2_^–^ while preserving NO bioavailability, or stabilize coadsorbed intermediates. This selection also provides a sensitive electronic test because NO and O_2_^–^ differ in charge, spin distribution, frontier orbital occupation, and hard/soft character. In this work, C-doping denotes substitutional C defects in the B_12_N_12_ BNF. Specifically, it refers to the B_12_N_12–*x*_C_*x*_ (*x* = 1–3) series, in which N sites are replaced by C according to the lowest-energy arrangements previously identified for these stoichiometries.^34^ Replacing N with C alters the valence-electron count and disrupts the local B–N symmetry, generating electron-deficient, spin-polarized defect states. This restricted series was chosen to isolate the effect of progressive N-site substitution at fixed B content. It is not intended to be an exhaustive survey of B-, mixed-, or all symmetry-inequivalent substitution patterns. Experimentally, B_12_N_12_ clusters have been detected by laser desorption time-of-flight mass spectrometry,^35^ and C incorporation has been demonstrated in extended BN/BCN materials.^36–38^ These studies support the plausibility of the parent BNF and of C incorporation into BNFs, but they do not constitute synthesis of the exact B_12_N_12–*x*_C_*x*_ molecular BNFs modeled here.

This work is based on a solid theoretical foundation of first-principles calculations within the framework of density functional theory (DFT). By combining structural, topological, kinetic-retention, and time-dependent DFT (TD-DFT) analyses, this study compares radical-specific adsorption mechanisms for NO@B_12_N_12‑*x*_C_*x*_ and O_2_^–^@B_12_N_12‑*x*_C_*x*_ (for *x* = 1–3) in gas-phase and aqueous environments. The results reveal composition-dependent radical-capture behavior and propose optical fingerprints whose practical applicability requires experimental validation. Topological analyses are also included to unravel the nature of the bonding interactions, coupled with an extensive assessment of dynamic kinetic stability using potential energy surface (PES) scans, Langevin first-passage simulations, and PES-based mean first-passage time (MFPT) projections. This approach reveals composition-dependent selectivity toward NO and O_2_^–^, as well as distinct finite-temperature retention and reconstruction regimes. These findings support the theoretical potential of C-doped BNFs as composition-tunable radical-capture platforms.

## 2 Computational Details

### 2.1 DFT Calculations

The interactions between BNFs (B_12_N_12_, B_12_N_11_C_1_, B_12_N_10_C_2_, B_12_N_9_C_3,_ and C_24_) and NO and O_2_^–^ were studied by first principles. Calculations were carried out using both the restricted and unrestricted spin formalisms to describe closed-shell and open-shell electronic configurations, respectively. DFT calculations were performed in both the gas phase and in aqueous solution. Solvation effects were modeled using an implicit water environment (the dielectric constant of water employed is represented by the value of ε = 78.3553 ^39,40^ ). Electronic-structure calculations were performed employing the Gaussian-16 package (Revision A.03).^41^ The structural and electronic properties were calculated using the range-separated hybrid functional ωB97X-D^42^ and the basis set 6-311++G­(d,p).^43^ To evaluate the sensitivity of the aqueous-phase results to the implicit solvent model, additional vertical single-point calculations were performed using the Polarizable Continuum Model (PCM) and the Solvation Model Based on Density (SMD)^44^ at the same ωB97X-D/6-311++G­(d,p) level. These calculations were performed on optimized aqueous geometries to isolate the effect of the continuum-solvent model and prevent changes in geometry, functional, or basis set. To evaluate the impact of local hydration on adsorption, microhydrated complexes with five explicit water molecules surrounding the BNF-adsorbate binding region were generated and optimized in PCM water using the same level. The results of this analysis are presented in the Section S7. The 5H_2_O cluster-continuum models were used for the primary explicit-water comparison and converged 10H_2_O models were used to check for size sensitivity.

The hybrid functional was employed to achieve an accurate treatment of electronic correlation and long-range noncovalent interactions. ωB97X-D combines a range-separated hybrid approach with empirical dispersion corrections,^42^ describing noncovalent interactions and electronic properties of complex molecular systems.

Gas-phase Boys–Bernardi counterpoise calculations were performed at the same ωB97X-D/6-311++G­(d,p) level using a two-fragment CP2 approach. The BNF and the adsorbed radical were treated as separate fragments to estimate the magnitude of the basis set superposition error (BSSE). Full BSSE diagnostics are provided in the Section S2.

The minimum energy position of the BNF-molecule complexes was determined by selecting ten probable interaction positions (using a test distance of 2.0 Å), prioritizing those areas with N–O, N–B, N–C, B–C, B–O, N–N, C–O and O–O bonds, in perpendicular and parallel positions. The analysis was based on obtaining molecular electrostatic potential (MEP) maps, granting visualization of charge distribution (nucleophilic and electrophilic regions) within structures.

To ensure the reliability of the open-shell calculations, spin purity was monitored by analyzing the ⟨*S* ^2^⟩ values before and after the standard spin-annihilation procedure (as summarized in Table S6). ⟨*S* ^2^⟩ is calculated as ⟨*S* ^2^⟩ = *s*(*s* + 1) with $s=\frac{M-1}{2}$ and *M* is the multiplicity of the system. For all C-doped BNFs, the resulting wave functions exhibit relatively slight changes in error with respect to the ideal eigenvalues (0.00, 0.75, 2.00, and 3.75 for the singlet, doublet, triplet, and quartet states, respectively). Residual spin contamination was negligible for these BN-based systems. C_24_–NO and C_24_–O_2_^–^ exhibited significant and minor residual contamination, respectively. The reported final energetic and electronic descriptors for the C_24_ complexes were obtained using the restricted open-shell formalism (RO–DFT). Unrestricted DFT (UDFT) spin-density distributions are interpreted qualitatively and are not used as evidence of multireference character or as proof of PES accuracy.

### 2.2 Quantum Descriptors Analysis

Upon selection of the ground-state configuration, structural and electronic properties were evaluated. This included quantum descriptors such as adsorption energy (*E*_ad_), the HL_gap_, dipole moment (DM), work function (WF), chemical potential (μ), electron affinity (EA), ionization potential (IP), polarizability (α), global hardness (η), global softness (*S*), and electrophilicity (ω).

Vibrational frequency analyses were conducted to validate the optimized structures as true minima on the PES. Within the harmonic approximation, the vibrational frequencies were obtained from the second derivatives of the electronic energy with respect to the nuclear displacements, as defined by the Hessian matrix. The structural stability of the optimized geometries was confirmed by the absence of imaginary frequencies.

The *E*_ad_ was determined according to the expression

$$E_{ad}=E_{BNF‐ROS/RNS}-E_{ROS/RNS}-E_{BNF}$$

where *E*_BNF‑ROS/RNS_, *E*_ROS/RNS,_ *E*_BNF_ correspond to the total energies of the complex, the isolated ROS/RNS and the BNF, respectively.

The coadsorption energies of NO and O_2_^–^ were calculated according to

$$E_{ad,NO}=E_{BNF+NO+O_{2}^{-}}-\left(E_{BNF+O_{2}^{-}}+E_{NO}\right)$$

$$E_{ad,O_{2}^{-}}=E_{BNF+NO+O_{2}^{-}}-\left(E_{BNF+NO}+E_{O_{2}^{-}}\right)$$

Here, the terms $E_{BNF+O_{2}^{-}}$ , *E*_NO_, *E*_BNF+NO_ and $E_{O_{2}^{-}}$ represent the energies obtained from single-point calculations for each reference system.

The HOMO–LUMO gap was calculated as HL_gap_ = |LUMO–HOMO| and HL_gap,↑_ = |ε_HOMO_ – ε_LUMO_|↑, HL_gap, ↓_ = |ε_HOMO_ – ε_LUMO_|↓ for polarized systems.^45^ Based on Koopmans’ theorem approximation,^46^ the IP and EA were expressed as IP = −*E*_HOMO_ and EA = −*E*_LUMO_. These values were used to derive the chemical potential μ ≈ (*E*_HOMO_ + *E*_LUMO_)/2, global hardness (η = (IP – EA)/2), global softness (*S* = 1/2η), and the electrophilicity index (ω = μ^2^/2η). The work function was evaluated as WF = *E*_LUMO_ – μ.^47^ Under the adopted frontier-orbital approximation the calculated WF is numerically equivalent to the global hardness and is not interpreted as an independent descriptor.

Calculations were carried out considering neutral (*Q* = 0|e|), anionic (*Q* = −1|e|), and cationic (*Q* = +1|*e*|) charge states. For each system, spin multiplicities (*M* = 2*S*_T_ + 1) were evaluated from singlet to sextet to determine the lowest-energy electronic state, with the selection guided by the electron count and molecular symmetry of the charged species.

A Natural Bond Orbital (NBO) analysis was performed on all optimized adsorption and coadsorption complexes to quantify the direction and magnitude of the charge transfer between the adsorbate and the BNF.^48^ Fragment charges were obtained by summing the NBO atomic charges over the BNF and adsorbate fragments. The Section S4 provides a full description of all atoms and fragments for the complexes. For the neutral NO complex, negative *q*(NO) values indicate electron donation from BNF to NO, whereas positive *q*(NO) values indicate electron donation from NO to BNF. For O_2_^–^, charge depletion relative to the isolated anion was estimated as *q*(O_2_^–^) + 1; positive values indicate partial O_2_^–^-to-BNF charge transfer. To complement the NBO analysis with a local reactivity descriptor, condensed Fukui functions were calculated on the extracted BNF geometries after removing the adsorbate from each optimized complex.^49^ Single-point calculations were performed for the *N*, *N* + 1, and *N* – 1 electron states without further structural relaxation. Hirshfeld atomic charges were used to compute *f*_*k*_^+^,*f*_*k*_^–^, *f*_*k*_^0^, and the dual descriptor Δ*f*_*k*_. This combined NBO–Fukui protocol was used to connect fragment charge transfer with local reactivity generated by C-substitution.

### 2.3 Topological Analyses

To characterize the nature of the adsorption interactions, Quantum Theory of Atoms in Molecules (QTAIM) analysis was conducted utilizing the Multiwfn software.^50^ This approach enabled the discrimination between covalent (chemisorption), and noncovalent (physisorption) bonding based on the extent of electron density redistribution and orbital stabilization.

Within the QTAIM formalism, bond critical points (BCPs) between the BNF and the adsorbate were located by analyzing the gradient field of the electron density, ∇ρ­(*r*). The bonding character was assessed based on the values of the electron density, its Laplacian, and the total energy density at the BCPs, with *H*(*r*) = *G*(*r*) + *V*(*r*). Following Cremer and Kraka’s criterion,^51^ a shared-shell interaction is generally associated with ∇^2^ρ­(*r*) < 0 and *H*(*r*) < 0, whereas closed-shell interactions typically exhibit ∇^2^ρ­(*r*) > 0 and *H*(*r*) > 0. Intermediate interactions with ∇^2^ρ­(*r*) > 0, *H*(*r*) < 0, and $0.5<\frac{G\left(r\right)}{\left|V\left(r\right)\right|}<1$ are described here as polar or partially covalent. Additionally, topological features were visualized through Electron Localization Function (ELF)^52^ and Localized Orbital Locator (LOL)^53^ analyses to map regions of electron localization/delocalization, thereby reinforcing the QTAIM interpretation.

The mapping of these functions was used to identify the covalent nature of chemisorbed interactions, which are characterized by significant electron localization between nuclei, from the noncovalent interactions observed in physisorbed systems.

### 2.4 Thermal Stability

Thermal stability was evaluated from relaxed one-dimensional potential-energy surfaces (1D-PES) calculated at the ωB97X-D/6-311++G­(d,p) level in the gas phase and with PCM water. For monodentate profiles, the reaction coordinate *q* was defined as the distance between the adsorbate anchoring atom and the initially bonded BNF atom. For bidentate O_2_^–^ configurations, the symmetric coordinate *q* = 0.5­(*d*_1_ + *d*_2_) was used, where *d*_1_ and *d*_2_ are the two original BNF–O distances. At each constrained optimization window, preservation of the initial bonding branch, fragment identity, O–O distance, charge, multiplicity, and electronic continuity was examined. Windows exhibiting reanchoring, contact switching, molecular collapse, duplicated geometries, or electronic-root discontinuities were excluded from interpolation and quantitative kinetic analysis. The relative energy profiles were defined as Δ*E*(*q*) = *U*(*q*) – *U*(*q*_min_). Shape-preserving piecewise-cubic Hermite interpolation (PCHIP) was applied only within structurally and electronically continuous PES segments for force evaluation and numerical integration. Missing or rejected windows were not bridged by interpolation.

Transition-state (TS) searches and bidirectional intrinsic reaction coordinate (IRC) calculations were performed to determine the applicability of formal transition-state theory (TST). The validity of a desorption TS required the presence of exactly one significant desorption-aligned imaginary frequency, as well as forward and reverse IRC paths connecting the adsorbed and dissociated states on the same bonding and electronic branch. No desorption candidate used in the present thermal-stability analysis fulfilled all conditions, so formal Eyring-TST and harmonic Arrhenius lifetimes were not reported.

For continuous PES profiles that increased monotonically toward a stable dissociation plateau, an effective escape threshold was defined as Δ*E*_eff_ = *U*(*q*_abs_) – *U*(*q*_min_), where *q*_min_ is the adsorption minimum and *q*_abs_ is an absorbing boundary located in the plateau region or immediately preceding a change in the bonding branch. The resulting Δ*E*_eff_ values are effective electronic desorption thresholds. They were not interpreted as stationary-point activation free energies.

The finite-temperature escape was simulated along the same coordinate using the underdamped Langevin equation

$$m_{eff}\mathrm{q̈}=-\frac{dU\left(q\right)}{dq}-\zeta \mathrm{q̇}+\sqrt{2\zeta k_{B}T}R\left(t\right)$$

With

$$\left\langle R\left(t\right)\right\rangle =0,,\left\langle R\left(t\right)R\left(t^{\prime }\right)\right\rangle =\delta \left(t-t^{\prime }\right)$$

Here, *q*(*t*) is the 1D desorption coordinate, *q̇* and *q̈* are its velocity and acceleration, respectively, and *U*(*q*) is the interpolated potential-energy profile. The effective inertial mass was approximated by the reduced mass of the atom pair defining the BNF–adsorbate contact

$$m_{eff}=\frac{m_{A}m_{B}}{m_{A}+m_{B}}$$

where *m*_A_ and *m*_B_ are the atomic masses of the BNF anchoring atom and the adsorbate atom, respectively. This approximation yielded effective masses of 6.10 amu for B–N contacts, 6.47 amu for C–N contacts, and approximately 6.45 amu for the B–O coordinate used for O_2_^–^. The parameter ζ is the effective friction coefficient, *k*_B_ is the Boltzmann constant, *T* is the absolute temperature, and *R*(*t*) is normalized Gaussian white noise. The Dirac delta function δ­(*t*–*t* ^′^) specifies that the stochastic fluctuations are temporally uncorrelated.

A reflecting boundary was imposed at *q*_min_, and rupture was measured when *q*_abs_ was first reached. The central friction coefficient of ζ = 5.0 × 10^–13^ kg s^–1^ was used. Trajectories were propagated using the BAOAB splitting scheme,^54^ which is designed for stable and accurate Langevin integration. Screening calculations comprised 500 independent 100 ps trajectories for each system and temperature at 21 temperatures between 298.15 and 2000 K. Direct rupture probabilities were obtained from 500 independent 100 ps trajectories. Probabilities at 1 ns, 1 μs, and longer times were obtained exclusively from the PES-based MFPT projections.

Direct 100 ps trajectories are unable to resolve long residence times for the high-energy surfaces. For this reason, the mean first-passage time (MFPT) was also calculated from the 1D-PES. The MFPT was defined as the ensemble-averaged time required for the desorption coordinate *q*(*t*), initially located at the adsorbed minimum *q*_min_, to reach the absorbing boundary *q*_abs_ for the first time.

$\tau_{MFPT}\left(q_{\min}\to q_{abs}\right)=\frac{\zeta }{k_{B}T}\int_{q_{\min}}^{q_{abs}}dye^{\beta U\left(y\right)}\int_{q_{\min}}^{y}dze^{-\beta U\left(z\right)}$ where $\beta ={\left(k_{B}T\right)}^{-1}$ and *y* and *z* are integration variables along the same desorption coordinate. Because the PES was referenced to *U*(*q*_min_) = 0, the relative potential Δ*E*(*q*) could equivalently be used in the integral. The corresponding rupture probability was calculated as

$$P_{des}\left(t;T\right)=1-\exp\left[-\frac{t}{\tau \left(T\right)}\right]$$

This first-passage formulation follows the diffusion-controlled escape framework introduced by Kramers^55^ and subsequently developed for friction-dependent reaction dynamics.^56^ Probabilities within the explicitly simulated 100 ps interval were reported as direct trajectory results, whereas probabilities at 1 ns, 1 μs, and longer times were identified as PES-MFPT projections. Thermal comparisons relevant to biological conditions were restricted to the near-ambient/physiological range of approximately 298.15–313.15 K.

To complement the 1D desorption analysis with an explicit finite-temperature structural test, DFT-based molecular dynamics trajectories were performed using the atomic density matrix propagation (ADMP) formalism^57^ implemented in Gaussian 16. Each trajectory was initiated from the corresponding optimized geometry, retaining the charge and spin multiplicity assigned in the static calculations. The electronic structure was evaluated at the same ωB97X-D/6-311++G­(d,p) level at every propagation step using FullSCF, an UltraFine integration grid, and SCF = (XQC, MaxCycle = 10,000, NoVarAcc). Nuclear motion was propagated with a 0.5 fs time step under a nominal 300 K thermostat, with the initial nuclear kinetic energy adjusted to the number of atoms in each system. System-dependent production lengths were used for the ADMP calculations, and the effective contiguous observation window obtained for each trajectory is reported explicitly in the Supporting Information. Gas-phase and PCM-water trajectories were considered for the radical-bound systems. The isolated B_12_N_12_, B_12_N_11_C_1_, B_12_N_10_C_2_, and B_12_N_9_C_3_ cages were analyzed independently to address intrinsic finite-temperature structural stability. Trajectory analysis distinguished cage deformation from adsorbate motion. Structural integrity of the BNF was quantified from the Kabsch-aligned RMSD^58^ of the 24 cage atoms and by monitoring preservation of the original cage-bond network. For radical-bound trajectories, the cage-only RMSD was evaluated together with the minimum adsorbate–cage separation, the internal N–O or O–O distance, and the defect-localized contacts involved in the optimized adsorption motif. This separation is necessary because desorption of a small fragment can generate a large whole-system RMSD even when the cage itself remains essentially unchanged. The trajectories were interpreted as finite observation windows: an observed bond-breaking, desorption, or reanchoring event was treated as direct dynamical evidence, whereas absence of an event was not converted into a long-time lifetime. PCM trajectories represent implicit aqueous solvation and do not reproduce explicit solvent collisions or hydrogen-bond dynamics.

### 2.5 Thermodynamic Analysis and Van’t Hoff Dependence

To assess the temperature dependence and the spontaneity of the radical scavenging process, a thermodynamic analysis was conducted using the Van’t Hoff equation. The equilibrium constant (*K*_ad_) for the adsorption of NO and O_2_^–^ on the BNFs is calculated based on the standard Gibbs free energy of adsorption (Δ*G*_ad_)­

$$K_{ad}\left(T\right)=\exp\left(-\frac{\Delta G_{ad}\left(T\right)}{RT}\right)$$

where *R* is the universal gas constant (8.314 × 10^–3^ kJ mol^–1^ K^–1^) and *T* is the absolute temperature. To extract the standard enthalpic (Δ*H*_ad_) and entropic (Δ*S*_ad_) contributions, the linear form of the Van’t Hoff relation was employed

$$\ln\;K_{ad}=-\frac{\Delta H_{ad}}{R}\left(\frac{1}{T}\right)+\frac{\Delta S_{ad}}{R}$$

The interaction was modeled across a temperature range of 298.15 to 700 K. *T*_eq_ was calculated as the model-derived temperature at which Δ*G*_ad_ = 0; values outside 298.15–700 K were treated only as qualitative extrapolations.

$$T_{eq}=\frac{\Delta H_{ad}}{\Delta S_{ad}}$$

### 2.6 Optical Signatures

The TD-DFT UV–Vis spectra were computed from the optimized ground-state geometries at the ωB97X-D/6-311++G­(d,p) level in gas and aqueous environments. Vertical excitation energies were calculated using the Franck–Condon approximation,^59,60^ and 40 excited states were obtained for each system to cover the UV–Vis region. Within linear-response TD-DFT, the excitation energies (ω) are obtained by solving the Casida equation:^61^

$$\left(\begin{array}{cc} A & B\\ B & A \end{array})(\begin{array}{c} X\\ Y \end{array})=\omega \left(\begin{array}{rr} 1 & 0\\ 0 & -1 \end{array}\right)(\begin{array}{c} X\\ Y \end{array}\right)$$

where the matrices **A** and **B** are defined as

$$\begin{array}{c} A_{ia,jb}=\delta_{ij}\delta_{ab}\left(\varepsilon_{a}-\varepsilon_{i}\right)+\left\langle ia∥jb\right\rangle \\ B_{ia,jb}=\left\langle ia∥bj\right\rangle \end{array}$$

with *i*, *j* denoting occupied orbitals, *a*, *b* virtual orbitals, and ε their corresponding Kohn–Sham orbital energies. The eigenvalues ω correspond to the vertical excitation energies, while the eigenvectors (*X*, *Y*) define the excitation character.

The intensity of each electronic transition was quantified through the oscillator strength (*f*),^62^ given by

$$f=\frac{2}{3}\omega {\left|\left\langle \Psi_{0}\left|\mathrm{\mu ̂}\right|\Psi_{n}\right\rangle \right|}^{2}$$

where μ̂ is the electric dipole moment operator, Ψ_0_ the ground-state wave function, and Ψ_*n*_ the excited-state wave function.

To assess solvent effects, additional TD-DFT calculations were carried out using implicit SMD with water as the solvent. All calculations considered the appropriate singlet or doublet multiplicity according to the closed- or open-shell character of each system. Excited-state population analyses were used to assign the dominant orbital transitions. UV–Vis spectra were simulated by Gaussian convolution of the oscillator strengths using an FWHM of 3000 cm^–1^ (≈0.37 eV), and the resulting bands were correlated with the electronic character of the corresponding transitions.

## 3 Results and Discussion

### 3.1 Structural and Electronic Features of BNFs

With the aim of evaluating the adsorption capacity of C-doped BNFs for ROS/RNS biosensing, the interactions between BNFs and NO/O_2_^–^ species were investigated. The geometric optimization of the pristine B_12_N_12_ and C-doped fullerenes (B_12_N_11_C_1_, B_12_N_10_C_2_, B_12_N_9_C_3_ and C_24_) was performed to establish ground-state reference properties. The ωB97X-D/6-311++G­(d,p) level of theory was applied to obtain the minimum energy complex. As summarized in Table 1, the pristine BNF is characterized by its high structural symmetry, with B–N bond lengths ranging from 1.43 to 1.48 Å and 1.90 Å for B–B bonds. Structural and vibrational analysis of the pristine B_12_N_12_ BNF (Figure 1) shows strong B–N bonds and a high-frequency vibrational mode at about 1445 cm^–1^. B_12_N_12_ has been shown to be highly stable and rigid,^63^ making it an ideal reference for studying the electronic modifications induced by C-doping. This stability is attributed to an efficient atomic arrangement that maximizes the strong heteropolar attraction within the B–N bonds.^64^ Electronically, this BNF is an insulator with a wide HL_gap_ of 10.94 eV in the gas phase, indicating high kinetic stability and low chemical reactivity. Its negligible DM and high global η confirm its chemically inert and insulating character.

**Table 1:** Bond Length (*l*; Å), HOMO–LUMO Gap (HL_gap_; eV), Dipole Moment (DM; Debye), Chemical Potential (μ; eV), Work Function (WF; eV), Electron Affinity (EA; eV), Ionization Potential (IP; eV), Polarizability (α; a. u.), Global Hardness (η; eV), Global Softness (*S*; eV^–1^), and Electrophilicity (ω; eV) for C-Doped BNFs and Test Molecules in Neutral Charge^a^

structure	B_12_N_12_		B_12_N_11_C_1_		B_12_N_10_C_2_		B_12_N_9_C_3_		C_24_		NO		O_2_	
property	gas	water	gas	water	gas	water	gas	water	gas	water	gas	water	gas	water
***L***	1.90, 1.91 (B–B)		1.89, 2.01 (B–B)		1.89, 1.91 (B–B)		1.86, 1.99 (B–B)		1.35, 1.52 (C–C)		1.14 (N–O)		1.19 (O–O)	
	1.43, 1.48 (B–N)		1.54, 1.57 (B–C)		1.54, 1.57 (B–C)		1.53, 1.59 (B–C)							
			1.43, 1.49 (B–N)		1.42, 1.48 (B–N)		1.43, 1.49 (B–N)							
							1.95 (C–C)							
HL_gap_	10.94	10.98	8.76	8.80	7.58	7.62	7.20	7.23	5.45	5.46	11.12	9.84	14.79	14.75
DM	0.00	0.01	0.44	0.69	0.37	0.54	0.29	0.42	0.00	0.01	0.09	0.11	0.00	0.00
μ	–4.63	–4.58	–5.34	–5.28	–5.54	–5.46	–5.53	–5.48	–5.21	–5.11	–6.19	–6.78	–5.73	–5.70
WF	5.47	5.49	4.38	4.40	3.79	3.81	3.60	3.61	2.72	2.73	5.56	4.92	7.40	7.38
EA^b^	0.37	2.09	3.44	5.17	3.25	4.90	3.24	4.66	2.87	4.47	0.25	3.25	0.40	3.52
IP^b^	9.80	8.01	8.96	7.12	8.15	6.37	8.19	6.43	7.38	5.58	9.58	6.31	12.50	9.16
α	156.37	221.65	163.10	233.79	170.20	247.81	176.84	260.19	209.40	349.91	9.28	10.58	8.26	9.48
η	5.47	5.49	4.38	4.40	3.79	3.81	3.60	3.61	2.72	2.73	5.56	4.92	7.40	7.38
*S*	0.09	0.09	0.11	0.11	0.13	0.13	0.14	0.14	0.18	0.18	0.09	0.10	0.07	0.07
ω	1.96	1.91	3.26	3.17	4.05	3.91	4.25	4.15	4.99	4.78	3.44	4.68	2.22	2.20

a Reported at the ωB97X-D/6-311++G­(d,p) level.

b The IP and EA were obtained from total energy differences as *IP*_ΔSCF_ = *E*(*N* – 1) – *E*(*N*) and EA_ΔSCF_ = *E*(*N*) – *E*(*N* + 1), respectively. The conceptual DFT descriptors μ, η, *S*, and ω were derived independently from the frontier-orbital energies. For open-shell systems, the reported frontier levels correspond to the arithmetic mean of the α- and β-spin values.

**Figure 1:**
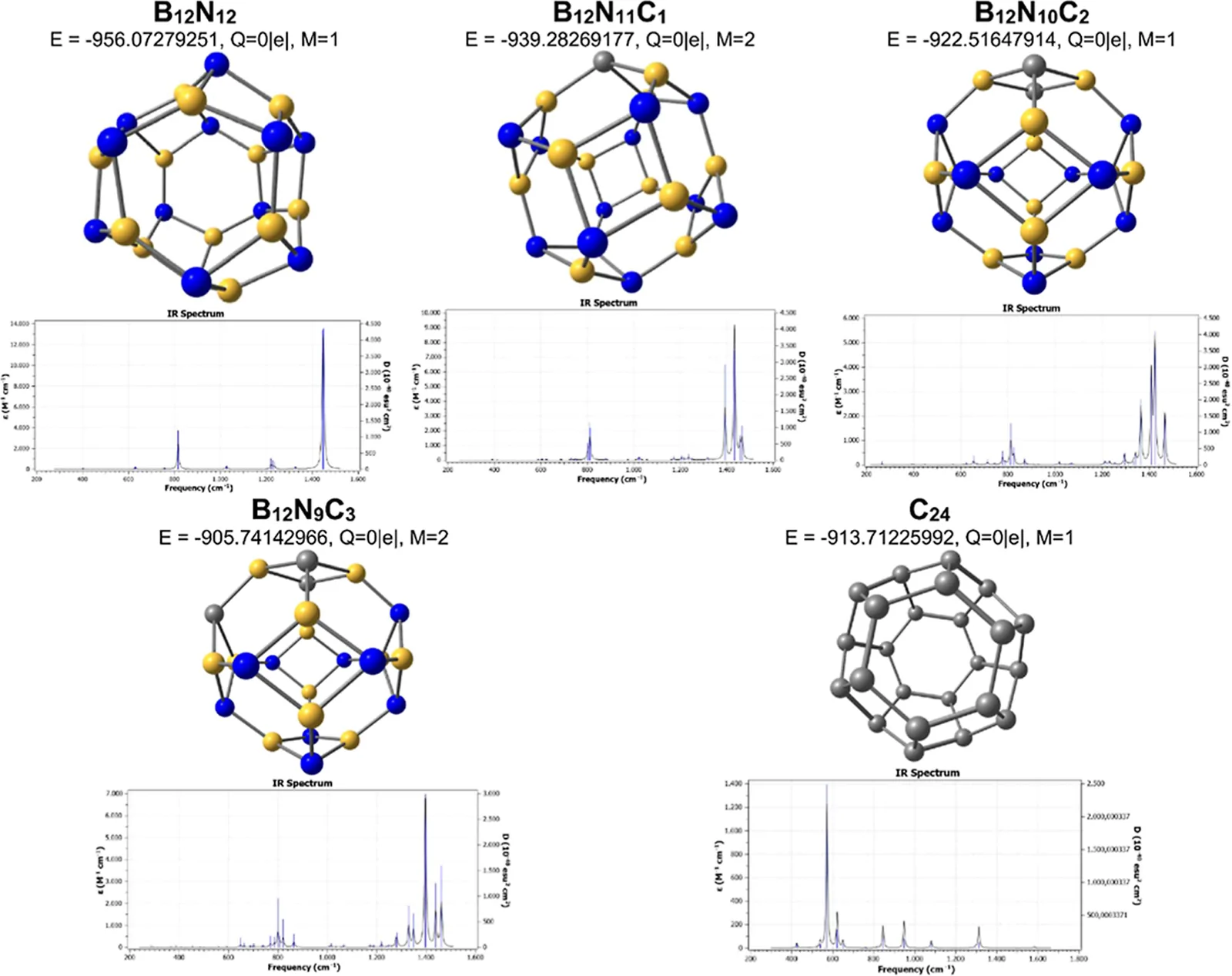
Graphical representation of the optimized structures (ball-and-stick model) of the BNFs. The minimum energy (E; Hartree), multiplicity (M), and global charge (*Q*) are also included, along with the theoretical IR spectra. B, N, and C atoms are distinguished by the colors yellow, blue, and gray, respectively.

Substituting N with C atoms introduces structural disorder and asymmetry, as well as significant electronic modulation. As a result, bond rigidity decreases, and the maximum absorption frequency is reduced to ∼1396 cm^–1^ for B_12_N_9_C_3_. C-doping reduces geometric strain energy, a critical thermodynamic requirement that drives molecular reorganization during chemisorption processes.^34^ In C-doped BNFs, the B–C bond lengths are longer than typical B–N bonds (1.53 to 1.59 Å), reflecting local strain induced by the C atom. The infrared (IR) spectra of the C-doped systems (Figure 1) exhibit an increased number of active peaks compared to the highly symmetric B_12_N_12_, reflecting a transition toward greater conformational flexibility and localized instability induced by C perturbations.^65^

A reduction of the HL_gap_ (from 10.94 eV in B_12_N_12_ to 7.20 eV in B_12_N_9_C_3_), an increase in polarizability, and an increase in global softness are induced by progressive C-doping. The monodoped B_12_N_11_C_1_ has a HL_gap_ of 8.76 eV, while the tridoped B_12_N_9_C_3_ shows a reduced HL_gap_ of 7.20 eV. Transition from a wide-gap insulator to a semiconductor-like behavior is driven by the formation of localized defect states within the bandgap. Midgap states, generated by the C-doping, act as bridges for electronic excitation, reducing the energy barrier for CT.^66^ Frontier Molecular Orbitals (FMO) analysis indicates that B_12_N_9_C_3_ is the most effective nucleophile (highest HOMO) and B_12_N_10_C_2_ is the most reactive electrophile (lowest LUMO). These findings imply that C-substitution enables dual control over the electron transfer ability of the nanostructure.^67^ Localized impurity states within the bandgap are introduced by C-doping. Disruption of the highly symmetrical BN cage and the introduction of localized electronic states associated with C–C and C–N bonding motifs enhance the fullerene’s ability to participate in CT processes. The substitution of C atoms introduces an electron deficiency into the system, leading to acceptor-like behavior (p-type doping). This electronic imbalance is also evident in the molecular orbital distribution, where localized states emerge near the HOMO, along with a progressive reduction in the HL_gap_ as the C content increases. C acts as an electron acceptor in the BN structure.

From an electronic view, B_12_N_12_ has a very low EA of 0.37 eV in the gas phase and a moderate EA of 2.09 eV in water, making it a poor candidate for accepting extra charge. The EA increases when the structure is doped with C. As the doping level increases (B_12_N_10_C_2_ and B_12_N_9_C_3_), the EA decreases slightly compared to B_12_N_11_C_1_ (3.25 and 3.24 eV in gas, respectively), although it remains higher than that of B_12_N_12_. Pure C_24_ exhibits a lower electron affinity (2.87 eV in gas). B_12_N_11_C_1_ is the most effective system for O_2_^–^ adsorption. Its high EA makes it the most efficient electron sink in these BNFs, which is thermodynamically ideal for stabilizing the extra negative charge provided by the anion.

B_12_N_12_ has the highest IP (9.80 eV in gas/8.01 eV in water), indicating its difficulty in donating electrons. When C is introduced, the IP decreases. C_24_ is the optimal pure donor (IP of 7.38 eV gas/5.58 eV water). B_12_N_10_C_2_ (IP = 8.15 eV gas) and B_12_N_9_C_3_ (IP = 8.19 eV gas) have the lowest IP among the C-doped BNFs, making them much more likely to give up electron density to NO than B_12_N_12_ or B_12_N_11_C_1_. Optimal NO adsorption is governed by the specific CT mechanism involved. Systems with higher C-doping exhibit the best thermodynamic balance, reducing the IP (improve donation) while maintaining a high EA (improve acceptance) compared to B_12_N_12_. This enhances their reactivity and versatility when exposed to a radical, such as NO. These C-doped BNFs exhibit enhanced electron transfer capabilities in an aqueous solvent medium compared to a vacuum, facilitating the chemical adsorption of NO and O_2_^–^.

C-doping enhances the chemical reactivity of BNFs, making them more suitable as active adsorption platforms. An increase in polarizability (α) indicates that the system redistributes its electron density more easily to stabilize the incoming molecule. As a result, the dispersion forces are strengthened, and the initiation of CT is more efficient. Polarizability increases progressively with the number of C atoms. The B_12_N_9_C_3_ system reaches 176.84 au in gas (and 260.19 au in water), making it the optimal choice. Its high α allows it to better accommodate the initial electronic repulsion and stabilize the adsorption complex through polarization forces. B_12_N_9_C_3_ shows promise in adsorbing O_2_^–^ or NO which facilitates interaction between FMO orbitals and leads to the formation of a chemical bond. Furthermore, the global η decreases from 5.47 to 3.60 eV, confirming that C-doped BNFs are softer and more reactive than pristine BNF. The fullerenes exhibit reactivity like that of pure CFs while preserving the structural stability of the BN structure. These effects are preserved in the aqueous phase, suggesting that the electronic activation induced by C is stable under solvation effects.

The reactivity of these BNFs is further enhanced by the electronic properties of the target molecules, NO and O_2_^–^ (Figure 2). O_2_^–^ has a lower hardness (4.35 eV) and higher softness compared to the neutral NO radical (5.56 eV), aligning with the Hard and Soft Acids and Bases (HSAB) principle. This principle predicts strong covalent interactions with the doped surfaces.^68^ The NO radical acts as a rigid, hard electron acceptor through its π* LUMO, whereas the O_2_^–^ acts as a flexible nucleophile due to the occupancy of its antibonding orbitals. This weakens the O–O bond and lowers its vibrational frequency to ∼ 1258 cm^–1^. O_2_^–^ tends to transfer or share part of that negative charge. Therefore, the ideal system for its adsorption must be an excellent electron acceptor. NO is a free radical with an unpaired electron. This amphoteric molecule acts as an electron donor or acceptor, depending on its interaction with other molecules.

**Figure 2:**
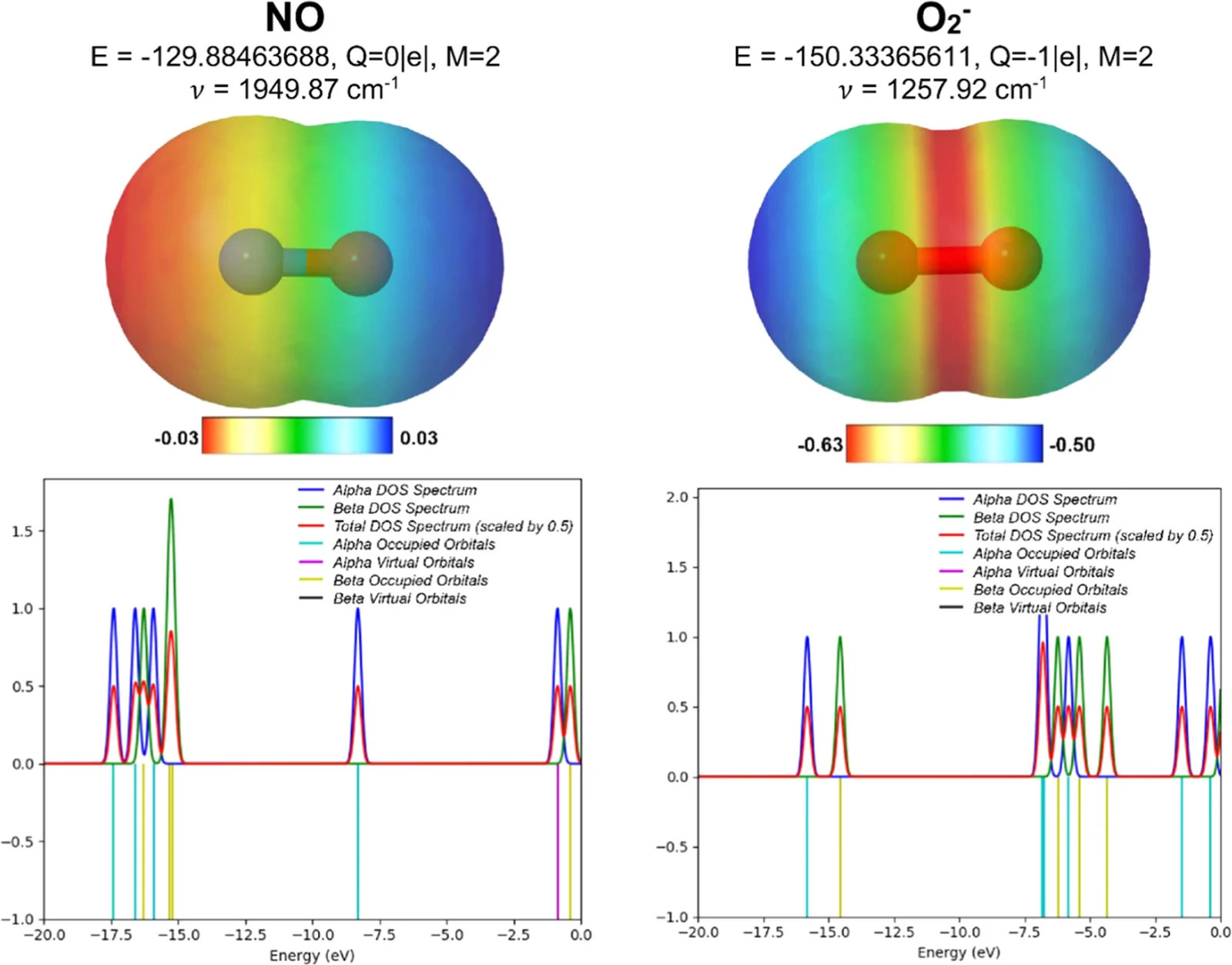
Thermochemical, electronic, and vibrational properties of NO and O_2_^–^ molecules. Included are the MEP maps of the optimized structures, minimum energy (*E*; Hartree), multiplicity (*M*), and global charge (*Q*), along with theoretical density of states (DOS) diagrams. N and O atoms are distinguished by blue and red colors, respectively.

### 3.2 Electronic Tuning via C-Doping

The open-shell character and distinct CT behavior of NO and O_2_^–^ suggest that adsorption can modify the electronic and spin distributions of the host BNF. The lowest-energy adsorption configurations and their corresponding spin states were examined. For the BN-based complexes, the values of *S* ^2^ after annihilation remained close to the ideal values for the assigned multiplicities, indicating negligible residual spin contamination for the electronic states considered.

The heteroatomic BNF and substitutional C defects promote the localized redistribution of spin density around adsorption sites (see Figures 3 and 4). C_24_ complexes exhibited greater residual spin contamination in unrestricted calculations, particularly C_24_–NO, suggesting that their UDFT wave functions are more susceptible to spin mixing. This observation alone does not establish multireference character or intrinsic electronic instability. Pure CFs support a more delocalized π-electron network,^69^ whereas C-doped BNFs introduce localized defect states that facilitate charge and spin redistribution upon adsorption. The combination of the *S* ^2^ diagnostics, spin-density distributions, CT results, and bonding analyses supports defect-mediated electronic coupling in the C-doped BNFs. Spin purity is interpreted here as a diagnostic of wave function quality, not as independent proof of stronger thermodynamic binding or biological performance.

**Figure 3:**
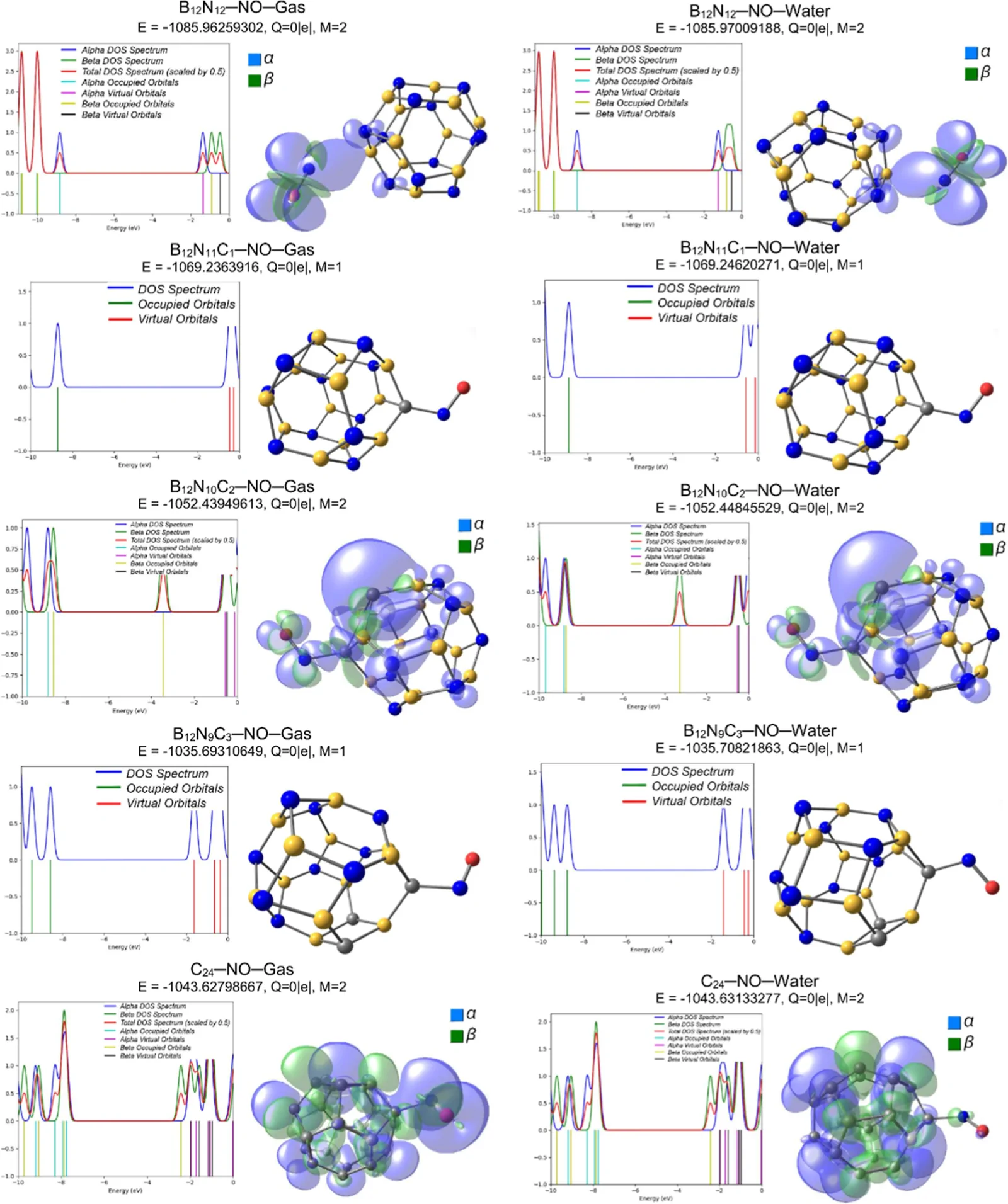
BNFs–NO interactions in gas and aqueous phases: density of states (DOS) profiles and spin density distributions for open-shell complexes (*M* > 1). The spin-density isosurfaces represent ρ_spin_(*r*) = ρ_α_(*r*) – ρ_β_(*r*), where ρ_α_(*r*) and ρ_β_(*r*) are the α- and β-electron densities. Blue regions indicate positive spin density, or local excess α-spin density. Green regions indicate negative spin density, or local excess β-spin density. B, C, N, and O atoms are shown in yellow, gray, blue, and red, respectively. Pristine B_12_N_12_–NO retains predominantly adsorbate-centered radical character, whereas the C-doped complexes exhibit defect-mediated spin delocalization involving the newly formed C–N contacts. Notably, C_24_ exhibits a pronounced change in spin polarization upon NO adsorption, evidenced by the asymmetry between the α- and β-spin contributions in the spin-resolved DOS. This behavior indicates electronic coupling between C_24_ and the unpaired electron of NO, accompanied by charge and spin redistribution and the emergence/modification of spin-dependent electronic states near the Fermi level, consistent with adsorption-induced modulation of the spin-polarized electronic structure.

**Figure 4:**
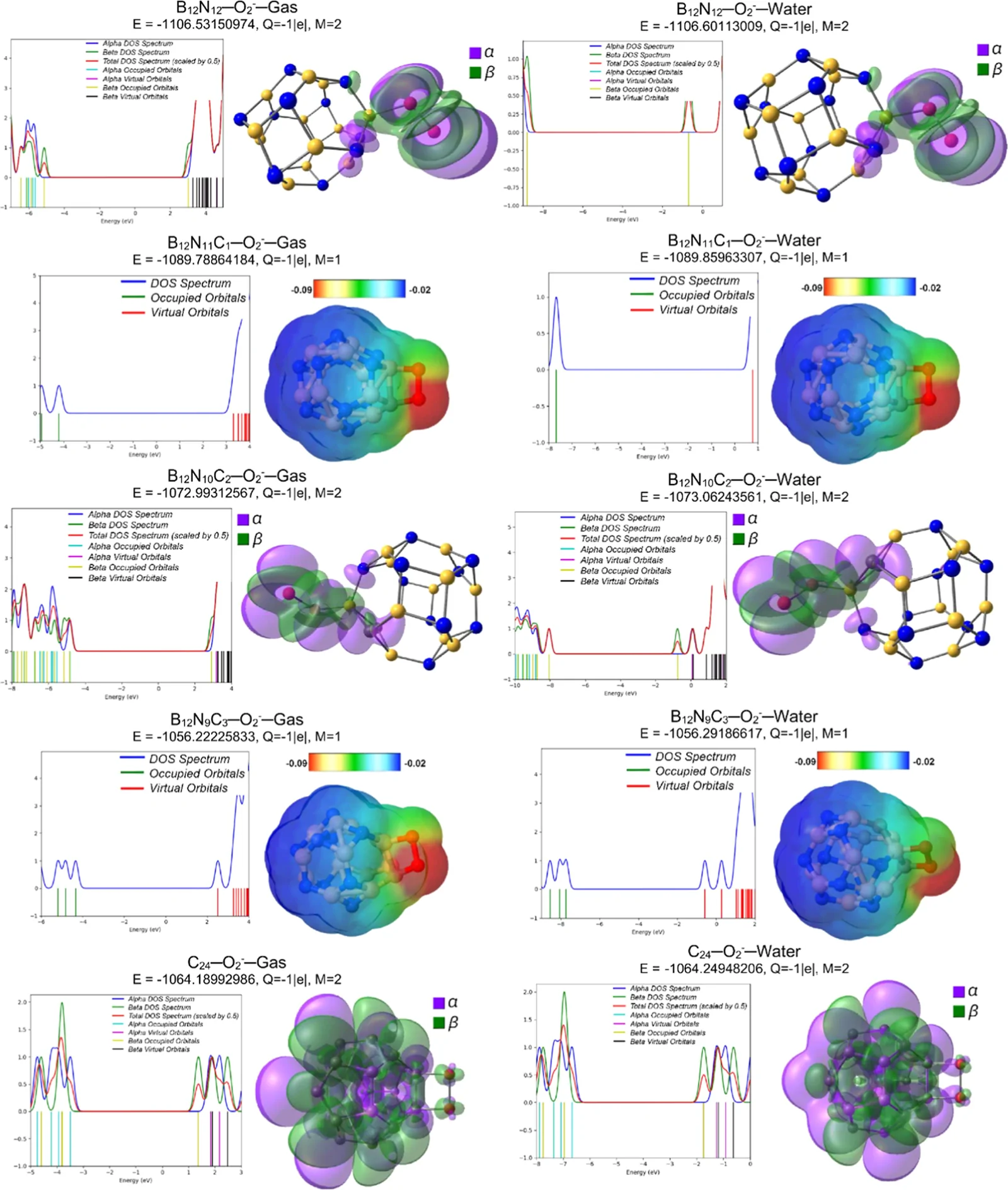
BNFs–O_2_^**–**^ interactions in gas and aqueous phases: density of states (DOS), molecular electrostatic potential (MEP), and spin density distributions of open-shell complexes (*M* > 1). The spin-density isosurfaces represent ρ_spin_(*r*) = ρ_α_(*r*) – ρ_β_(*r*), where ρ_α_(*r*) and ρ_β_(*r*) are the α- and β-electron densities. Violet regions indicate positive spin density, or local excess α-spin density. Green regions indicate negative spin density, or local excess β-spin density. The B, C, N, and O atoms are distinguished by the colors yellow, gray, blue, and red, respectively. **Note:** A change in the spin polarization of C_24_ is observed upon O_2_^**–**^ adsorption, as evidenced by the asymmetry between the α and β contributions in the spin density diagram.

Adsorption results indicate two distinct mechanisms governed by the presence of C dopants (see Tables 2 and 3). For the pristine B_12_N_12_ fullerene, the adsorption of neutral NO is characterized by a weak interaction energy (*E*_ad_ = −0.14 eV) and a large equilibrium distance of 2.48 Å (B–N). As illustrated in Figure 3, this is characteristic of physisorption driven by weak vdW forces, positioning the pristine structure as an ineffective scavenger for neutral NO. According to the QTAIM analysis in Table 4, the nature of the interaction is confirmed as a weak electrostatic type, as evidenced by the low ρ at the BCP and a positive Laplacian. The ELF and LOL analyses provide complementary insight into the bonding nature beyond QTAIM descriptors. For pristine B_12_N_12_–NO systems, the low ELF (0.12–0.17) and LOL (0.27–0.31) values indicate highly delocalized electron density at the BCP, consistent with weak electrostatic interactions as suggested by positive Laplacian and near-zero energy density.

**Table 2:** Adsorption Energy (*E*_ad_; eV), Bond Length (*l*; Å), HOMO–LUMO Gap (HL_gap_; eV), Dipole Moment (DM; Debye), Chemical Potential (μ; eV), Work Function (WF; eV), Polarizability (α; a. u.), Global Hardness (η; eV), Global Softness (*S*; eV^–1^), and Electrophilicity (ω; eV) in Gas and Aqueous Phases for BNFs + NO in Neutral State^a^

structure	B_12_N_12_–NO		B_12_N_11_C_1_–NO		B_12_N_10_C_2_–NO		B_12_N_9_C_3_–NO		C_24_–NO^b^	
quantum descriptor	gas	water	gas	water	gas	water	gas	water	gas	water
*E*_ad_	–0.14	–0.13	–1.88	–1.96	–1.04	–1.09	–1.82	–2.06	–0.85	–0.89
*L*	2.48 (B–N)	2.35 (B–N)	1.46 (C–N)	1.44 (C–N)	1.47 (C–N)	1.44 (C–N)	1.45 (C–N)	1.42 (C–N)	1.51 (C–N)	1.49 (C–N)
HL_gap_	8.28	8.35	8.26	8.30	6.68	6.84	6.96	7.36	4.25	4.20
DM	1.08	1.82	2.88	3.99	2.33	3.89	3.05	5.06	1.92	2.64
μ	–5.26	–5.19	–4.57	–4.72	–5.34	–5.34	–5.11	–5.09	–5.81	–5.66
WF	4.14	4.18	4.13	4.15	3.34	3.42	3.48	3.68	2.12	2.10
α	170.83	241.05	176.60	250.45	198.72	275.45	189.29	275.13	223.28	367.18
η	4.14	4.18	4.13	4.15	3.34	3.42	3.48	3.68	2.12	2.10
*S*	0.12	0.12	0.12	0.12	0.15	0.15	0.14	0.14	0.24	0.24
ω	3.35	3.22	2.53	2.69	4.26	4.16	3.75	3.52	7.95	7.64

a Reported at the ωB97X-D/6-311++G­(d,p) level.

b Open-shell calculations were screened using ⟨*S* ^2^⟩ diagnostics. RO-DFT values were used for the C_24_ complexes.

**Table 3:** Adsorption Energy (*E*_ad_; eV), Bond Length (*l*; Å), HOMO–LUMO Gap (HL_gap_; eV), Dipole Moment (DM; Debye), Chemical Potential (μ; eV), Work Function (WF; eV), Polarizability (α; a. u.), Global Hardness (η; eV), Global Softness (*S*; eV^–1^), and Electrophilicity (ω; eV) in Gas and Aqueous Phases for BNFs + O_2_^–^ ^a^

structure	B_12_N_12_–O_2_^–^		B_12_N_11_C_1_–O_2_^–^		B_12_N_10_C_2_–O_2_^–^		B_12_N_9_C_3_–O_2_^–^		C_24_–O_2_^–^ ^b^	
quantum descriptor	gas	water	gas	water	gas	water	gas	water	gas	water
*E*_ad_	–3.40	–1.98	–4.69	–3.32	–3.89	–2.48	–4.00	–2.62	–3.92	–2.39
*L*	1.49 (B–O)	1.51 (B–O)	1.43 (C–O)	1.43 (C–O)	1.47 (B–O)	1.49 (B–O)	1.42 (C–O)	1.43 (C–O)	1.44 (C–O)	1.44 (C–O)
			1.46 (B–O)	1.48 (B–O)			1.49 (B–O)	1.51 (B–O)	1.44 (C–O)	1.44 (C–O)
HL_gap_	8.54	9.12	7.50	8.45	7.88	7.72	6.87	7.15	2.71	3.78
DM	5.75	8.48	5.39	8.27	6.42	9.29	4.79	7.50	0.29	4.71
μ	–1.12	–4.38	–0.44	–3.45	–0.90	–4.20	–0.91	–4.15	–2.25	–4.88
WF	4.27	4.56	3.75	4.23	3.94	3.86	3.43	3.57	1.35	1.89
α	178.34	250.57	182.35	258.89	190.59	274.28	199.01	291.80	245.63	414.19
η	4.27	4.56	3.75	4.23	3.94	3.86	3.43	3.57	1.35	1.89
*S*	0.12	0.11	0.13	0.12	0.13	0.13	0.15	0.14	0.37	0.26
ω	0.15	2.10	0.03	1.41	0.10	2.28	0.12	2.41	1.87	6.29

a Reported at the ωB97X-D/6-311++G­(d,p) level.

b Open-shell calculations were screened using ⟨*S* ^2^⟩ diagnostics. RO–DFT values were used for the C_24_ complexes.

**Table 4:** QTAIM, ELF, and LOL Analysis of BNFs–NO Interactions in Both Gas and Aqueous Phase, Evaluated in BCPs (Atomic Units)

complex	bond	*H*(*r*)	∇^2^ρ	ρ	*G*(*r*)	V(r)	*G*(*r*)/|*V*(*r*)|	ELF	LOL	bond type
B_12_N_12_–NO (gas)	B–N	–0.0003	0.0450	0.0197	0.0115	–0.0118	0.9759	0.1229	0.2695	weak electrostatic interaction
B_12_N_12_–NO (water)	B–N	–0.0022	0.0495	0.0257	0.0146	–0.0168	0.8681	0.1754	0.3134	weak electrostatic interaction
B_12_N_11_C_1_–NO (gas)	C–N	–0.1191	–0.0072	0.1353	0.1173	–0.2364	0.4962	0.4323	0.4660	covalent bond
B_12_N_11_C_1_–NO (water)	C–N	–0.3166	–0.7137	0.2783	0.1382	–0.4548	0.3038	0.8586	0.7113	covalent bond
B_12_N_10_C_2_–NO (gas)	C–N	–0.2681	–0.5871	0.2591	0.1213	–0.3893	0.3115	0.8616	0.7138	covalent bond
B_12_N_10_C_2_–NO (water)	C–N	–0.3092	–0.6816	0.2739	0.1388	–0.4481	0.3099	0.8510	0.7050	covalent bond
B_12_N_9_C_3_–NO (gas)	C–N	–0.2972	–0.6907	0.2738	0.1245	–0.4217	0.2953	0.8763	0.7269	covalent bond
B_12_N_9_C_3_–NO (water)	C–N	–0.3652	–0.8066	0.2942	0.1636	–0.5288	0.3093	0.8392	0.6955	covalent bond
C_24_–NO (gas)	C–N	–0.2380	–0.5455	0.2463	0.1016	–0.3397	0.2992	0.8828	0.7329	covalent bond
C_24_–NO (water)	C–N	–0.2615	–0.4861	0.2293	0.1399	–0.4014	0.3486	0.7639	0.6419	covalent bond

C-doped systems show strong NO chemisorption. B_12_N_11_C_1_ shows the strongest gas-phase adsorption (−1.88 eV), and B_12_N_9_C_3_ shows the strongest aqueous adsorption (−2.06 eV). Both form short C–N bonds. The increase in binding strength is attributed to the high spin density localized on the C dopant. This facilitates covalent bond formation with the unpaired electron of the NO radical. The result is a closed-shell BNF–NO complex and the loss of its magnetic character.^70^ The CP2 correction slightly weakens the interaction energies without changing the adsorption ranking or bonding assignments. The pristine B_12_N_12_–NO shifts from approximately −0.14 to −0.11 eV, while the relative corrections for the C-doped NO and O_2_^–^ complexes are about 3–6% and 2–3%, respectively. All BSSE results are included in the Section S2.

The NBO/Fukui analysis shows that C-substitution modifies the direction and magnitude of charge redistribution upon adsorption. In pristine B_12_N_12_–NO, the NO fragment is positively charged (+0.102 e in the gas phase and +0.136 e in the aqueous phase), indicating a net donation of electrons from the NO fragment to the BNF. In contrast, C-doped BNFs exhibit negative NO fragment charges, indicating BNF-to-NO electron donation. The charges are −0.144/–0.189 *e* for B_12_N_11_C_1_–NO, −0.112/–0.191 *e* for B_12_N_10_C_2_–NO, and −0.158/–0.248 *e* for B_12_N_9_C_3_–NO in gas and water, respectively. Combined NBO and condensed Fukui analyses demonstrated that the radical-scavenging behavior of the five BNFs was controlled by local defect reactivity rather than by global HL_gap_ alone. C-doping reverses the CT direction of NO, resulting in BNF-to-NO electron donation in B_12_N_11_C_1_, B_12_N_10_C_2_, and B_12_N_9_C_3_. B_12_N_11_C_1_ exhibits the most localized defect-mediated response.

Topological descriptors such as ELF and LOL support the covalent nature of the bond, as evidenced by high ρ and negative local energy density (*H*(*r*) < 0), indicating shared-shell interactions (Table 4). C-doped systems exhibit higher ELF (0.43–0.86) and LOL (0.46–0.71) values, revealing pronounced electron localization and confirming the formation of covalent C–N bonds. The corresponding QTAIM, ELF, and LOL descriptors for O_2_^–^ adsorption are summarized in Table 5.

**Table 5:** QTAIM, ELF, and LOL Analysis of BNFs–O_2_^–^ Interactions in Both Gas and Aqueous Phase, Evaluated in BCPs (Atomic Units)

complex	bond	*H*(*r*)	∇^2^ρ	ρ	*G*(*r*)	*V*(*r*)	*G*(*r*)/|*V*(*r*)|	ELF	LOL	bond type
B_12_N_12_–O_2_^–^ (gas)	B–O	–0.1093	0.5119	0.1508	0.2373	–0.3467	0.6846	0.2109	0.3408	polar/partially covalent interaction
B_12_N_12_–O_2_^–^ (water)	B–O	–0.0993	0.4947	0.1423	0.2230	–0.3223	0.6918	0.1995	0.3330	polar/partially covalent interaction
B_12_N_11_C_1_–O_2_^–^ (gas)	C–O	–0.3263	–0.3193	0.2489	0.2465	–0.5728	0.4303	0.5682	0.5343	covalent bond
B_12_N_11_C_1_–O_2_^–^ (gas)	B–O	–0.0950	–0.0681	0.1103	0.0780	–0.1730	0.4507	0.4659	0.4830	covalent bond
B_12_N_11_C_1_–O_2_^–^ (water)	C–O	–0.3188	–0.2716	0.2445	0.2509	–0.5697	0.4404	0.5446	0.5224	covalent bond
B_12_N_11_C_1_–O_2_^–^ (water)	B–O	–0.1201	0.5071	0.1578	0.2469	–0.3671	0.6727	0.2231	0.3489	polar/partially covalent interaction
B_12_N_10_C_2_–O_2_^–^ (gas)	B–O	–0.1110	0.5870	0.1555	0.2578	–0.3688	0.6990	0.2006	0.3337	polar/partially covalent interaction
B_12_N_10_C_2_–O_2_^–^ (water)	B–O	–0.1021	0.5828	0.1485	0.2478	–0.3498	0.7083	0.1890	0.3256	polar/partially covalent interaction
B_12_N_9_C_3_–O_2_^–^ (gas)	B–O	–0.1187	0.4959	0.1556	0.2427	–0.3614	0.6715	0.2210	0.3475	polar/partially covalent interaction
B_12_N_9_C_3_–O_2_^–^ (gas)	C–O	–0.3368	–0.3602	0.2538	0.2467	–0.5835	0.4228	0.5837	0.5421	covalent bond
B_12_N_9_C_3_–O_2_^–^ (water)	B–O	–0.1089	0.4762	0.1474	0.2280	–0.3369	0.6767	0.2114	0.3412	polar/partially covalent interaction
B_12_N_9_C_3_–O_2_^–^ (water)	C–O	–0.3287	–0.3140	0.2491	0.2502	–0.5788	0.4322	0.5616	0.5309	covalent bond
C_24_–O_2_^–^ (gas)	C–O	–0.3232	–0.5325	0.2540	0.1901	–0.5132	0.3703	0.7030	0.6061	covalent bond
C_24_–O_2_^–^ (gas)	C–O	–0.3228	–0.5327	0.2539	0.1897	–0.5125	0.3701	0.7036	0.6064	covalent bond
C_24_–O_2_^–^ (water)	C–O	–0.3198	–0.5254	0.2526	0.1884	–0.5082	0.3708	0.7028	0.6060	covalent bond
C_24_–O_2_^–^ (water)	C–O	–0.3198	–0.5254	0.2526	0.1884	–0.5082	0.3708	0.7028	0.6060	covalent bond

NO adsorption on C_24_ relies on delocalized π interactions, resulting in reduced charge sensitivity and limited spin coupling modulation. Eliminating spin contamination from RO formalism in complexes with C_24_ corrected the overestimation of *E*_ad_. The value of −0.85 eV indicates weak chemisorption. The presence of NO introduces states that reduce the HL_gap_, increasing the reactivity of the system. The complex is more likely to accept electron density than isolated C_24_. The RO calculations result in a reduction in spin polarization, producing more symmetrical α/β DOS profiles and a delocalized spin density. By contrast, the original unrestricted-like results exhibit a clear spin asymmetry, suggesting that spin polarization is an inherent feature of the NO–C_24_ interaction. RO–DFT gives *E*_ad_ = −0.85 eV for C_24_–NO, consistent with weak chemisorption. UDFT spin-density maps are retained only qualitatively because residual spin contamination remains significant; they suggest a delocalized spin response but are not used to quantify magnetic coupling.^71^

Electronic heterogeneity in doped BN fullerenes offers superior tunability of NO binding compared to homonuclear CFs. As shown in Table 6, studies of ionic charge state that electronic charge influences reactivity. Anionic states enhance the binding affinity in B_12_N_12_ (*E*_ad_ = −1.65 eV) and B_12_N_10_C_2_ (*E*_ad_ = −2.14 eV) for NO adsorption, resulting in a physisorption-to-chemisorption transition. Back-donation from the electron-rich surface to the antibonding orbitals of the NO molecule is the driving force behind this process. Cationic complexes frequently display weaker or less stable bonding motifs, where the interaction is primarily driven by electrostatic polarization as opposed to covalent stabilization.^72^

**Table 6:** Adsorption Energy (*E*_ad_; eV), Bond Length (*l*; Å), HOMO–LUMO Gap (HL_gap_; eV), Dipole Moment (DM; Debye), Chemical Potential (μ; eV), Work Function (WF; eV), Polarizability (α; a. u.), Global Hardness (η; eV), Global Softness (*S*; eV^–1^), and Electrophilicity (ω; eV) in **Aqueous Phase** for BNFs + NO in Anionic and Cationic States^a^

structure	B_12_N_12_–NO		B_12_N_11_C_1_–NO		B_12_N_10_C_2_–NO		B_12_N_9_C_3_–NO		C_24_–NO^b^	
quantum descriptor	anion	cation	anion	cation	anion	cation	anion	cation	anion	cation
*E*_ad_	–1.65	–0.33^a^	–0.08	0.07	–2.14	–1.36	–0.38	–0.28	–1.31	–1.38
***L***	1.56 (B–N)	2.26 (N–N)	3.27 (C–N)	1.65 (C–N)	1.39 (C–N)	1.34 (C–N)	3.15 (N–N)	2.31 (N–N)	1.48 (C–N)	1.50 (C–N)
							3.48 (N–O)			
HL_gap_	8.84	6.77	7.28	7.30	8.49	7.17	7.00	5.78	5.55	1.13
DM	8.26	16.24	4.55	9.20	8.67	5.58	6.40	16.30	2.01	3.84
μ	–3.87	–6.94	–4.06	–6.78	–3.94	–6.86	–4.02	–6.53	–4.03	–3.57
WF	4.42	3.39	3.64	3.65	4.25	3.58	3.50	2.89	2.78	0.56
α	255.61	248.54	274.55	245.54	285.16	267.95	300.94	295.81	429.99	358.11
η	4.42	3.39	3.64	3.65	4.25	3.58	3.50	2.89	2.78	0.56
*S*	0.11	0.15	0.14	0.14	0.12	0.14	0.14	0.17	0.18	0.89
ω	1.70	7.12	2.26	6.29	1.83	6.57	2.30	7.36	2.93	11.27

a Reported at the ωB97X-D/6-311++G­(d,p) level.

b Open-shell calculations were screened using ⟨*S* ^2^⟩ diagnostics. RO-DFT values were used for the C_24_ complexes.

Adsorption of O_2_^–^ is stronger and more exothermic across all BNFs compared to NO (Table 3). B_12_N_11_C_1_–O_2_^–^ complex reached a maximum *E*_ad_ of −4.69 eV in the gas phase. Structural analysis indicates that O_2_^–^ prefers a “bridging” configuration (Figure 4), where it interacts simultaneously with two adjacent atoms of the BNF, maximizing orbital overlap.^73^ Cooperative stabilization through B–O and C–O motifs mimics the epoxy- or peroxo-intermediates observed in carbonaceous surfaces, effectively activating the oxygen species for further catalytic or sensing processes.^74^ For O_2_^–^ adsorption, the O_2_^–^ fragment remains negatively charged but is depleted relative to isolated O_2_^–^, confirming partial O_2_^–^-to-BNF charge transfer. The C dopants carry substantial negative NBO charge, and the closest adsorption contacts occur at or near the C-doped region. This supports the proposed electronic activation mechanism and the enhanced reactivity of the C-doped structures.

For BNFs–O_2_^–^ complexes, a dual bonding character is observed. B–O interactions exhibit moderate ELF and LOL values (≈0.19–0.34), suggesting partially localized density consistent with polar or partially covalent interactions. C–O bonds, on the other hand, display high ELF (up to 0.88) and LOL values, supporting strong covalent character. The results distinguish the polar or partially covalent B–O interactions that are already present in pristine B_12_N_12_–O_2_^–^ from the more strongly localized C–O shared-shell interactions that are introduced at selected C-doped sites.

Solvation effects in the aqueous phase generally weaken the adsorption strength. The polar medium stabilizes the isolated species and competes for interaction sites, reducing the net thermodynamic drive for complex formation.^75^ Systems such as the B_12_N_10_C_2_ anion display notable stability under solvation, maintaining high *E*_ad_ values. The solvent also increases the DM and α, indicating the dielectric response and the stabilization of charge separation. An increase in DM indicates electrostatic polarization rather than covalent stabilization. These results emphasize the critical importance of electronic charge state in modulating adsorption strength and bonding character, with anionic conditions proving highly effective for NO capture. The PCM/SMD comparison shows that chemisorption and physisorption behaviors are not controlled by the choice of implicit solvent model. For NO adsorption, C-doped BNFs interact more strongly than pristine B_12_N_12_ in both solvent descriptions. The corrected vertical comparison for B_12_N_10_C_2_–NO yields *E*_ad_ values of −1.09 to −1.27 eV in PCM and SMD. For O_2_^–^ adsorption, the solvent-model effect is larger. As expected for an anionic radical, the interactions remain strongly exothermic. B_12_N_10_C_2_–O_2_^–^ changes from −2.48 eV in PCM to −2.07 eV in SMD, and B_12_N_9_C_3_–O_2_^–^ changes from −2.62 to −2.25 eV. SMD generally weakens aqueous O_2_^–^ adsorption energies relative to PCM but maintains the conclusion that C-doped BNFs efficiently anchor O_2_^–^.

Comparing the continuum-only aqueous adsorption energies to those with five explicit first-shell waters made adsorption more favorable for all complexes. For O_2_^–^, the explicit-water correction ranged from −0.554 to −1.801 eV, with a mean of −1.019 eV. NO exhibited a more heterogeneous BNF dependence, with a range of −0.119 to −1.807 eV (mean −1.071 eV). These results indicate that directional hydrogen bonding and local polarization are not fully represented by the continuum model. A comparison with unsolvated 5H_2_O clusters showed that PCM weakens very large O_2_^–^ interactions, indicating that local microhydration and long-range dielectric screening act in opposite directions.^76^ The 10H_2_O calculations were used to check the sensitivity of the subset that converged with intact water networks. Six direct 5H_2_O-to-10H_2_O PCM comparisons were obtained. Five of these changed by no more than 0.213 eV; however, B_12_N_10_C_2_–O_2_^–^ became 1.309 eV more favorable with ten waters. Five waters thus capture the principal first-shell correction for most tested systems, while the B_12_N_10_C_2_–O_2_^–^ case remains sensitive to the extended hydration-network configuration.

Spin-state changes provide a complementary descriptor of radical adsorption. For the NO complexes, B_12_N_12_–NO and B_12_N_10_C_2_–NO retain open-shell doublet states, corresponding to approximately one unpaired electron, whereas B_12_N_11_C_1_–NO and B_12_N_9_C_3_–NO adopt singlet states, consistent with spin pairing between the NO radical and the C-defect region. The spin-density maps show predominantly adsorbate-centered radical character for weakly bound B_12_N_12_–NO and greater interfacial spin redistribution across the newly formed C–N contacts in the C-doped complexes. Because spin polarization may also originate from the unpaired electron of the adsorbate, these changes are interpreted together with adsorption energies, bond distances, NBO charge redistribution, and QTAIM/ELF/LOL descriptors, rather than as independent proof of electronic coupling. The corresponding composition- and solvent-dependent electronic descriptors are summarized in Figure 5. For C_24_, the residual spin contamination limits the interpretation of the spin-density maps to a qualitative comparison.

**Figure 5:**
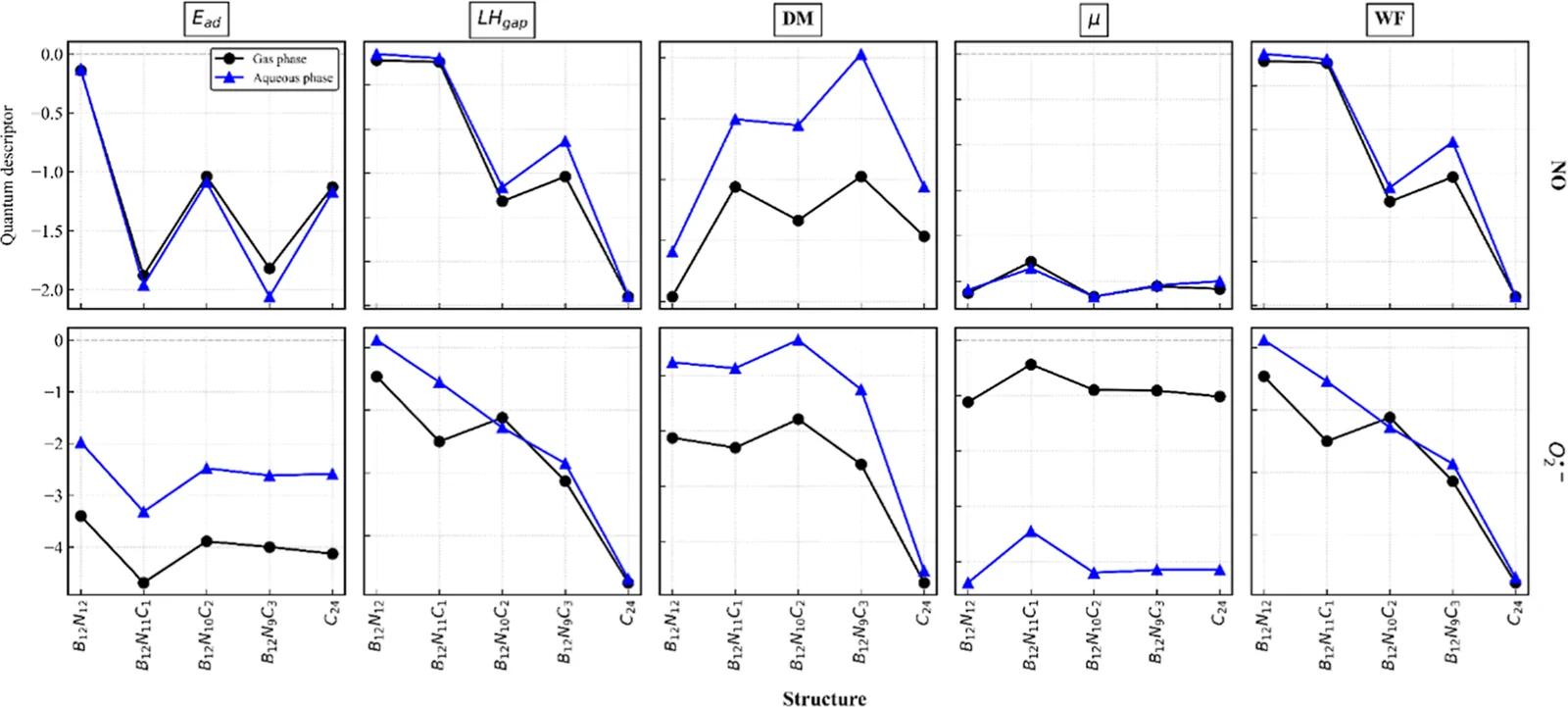
Comparative variation of quantum descriptors (adsorption energy, HL_gap_, dipole moment, chemical potential, and work function) for the B_12_N_12_, B_12_N_11_C_1_, B_12_N_10_C_2_, B_12_N_9_C_3_ and C_24_ structures in the gas and aqueous phases, in the adsorption of NO and O_2_^–^. Black: gas phase, blue: aqueous phase.

This study’s results provide evidence of mechanistic progression. C-doping activates the inert pristine BNF, enabling high-affinity chemisorption of NO and O_2_^–^ through covalent C–N and bridging C–O/B–O bonds. Localized electronic heterogeneity and environmental stabilization create a synergistic effect, positioning doped BNFs as promising platforms for gas sensing and environmental remediation. Overall, the results indicate a well-defined mechanistic progression: **(i)** C-doping activates BNFs electronically, **(ii)** NO adsorption evolved from weak electrostatic interaction to chemisorption via C–N bond formation, **(iii)** O_2_^–^ exhibits strong covalent binding enhanced by CT, and **(iv)** solvent effects stabilize adsorption without altering bonding nature. The synergy between structural modification and electronic tuning positions C-doped BNFs as highly promising nanostructures for gas sensing and/or catalytic redox processes.

### 3.3 Co-Adsorption of NO and O_2_^–^

Building upon the insights gained from the individual interactions, the coadsorption of NO and O_2_^–^ was modeled to assess whether coadsorption can yield a surface-bound ONOO-like structure. The optimized geometries of the anionic BNFs–NO + O_2_^–^ complexes are presented in Figure 6, and Table 7 summarizes the corresponding adsorption energies and global reactivity descriptors in both gas and aqueous phases.

**Figure 6:**
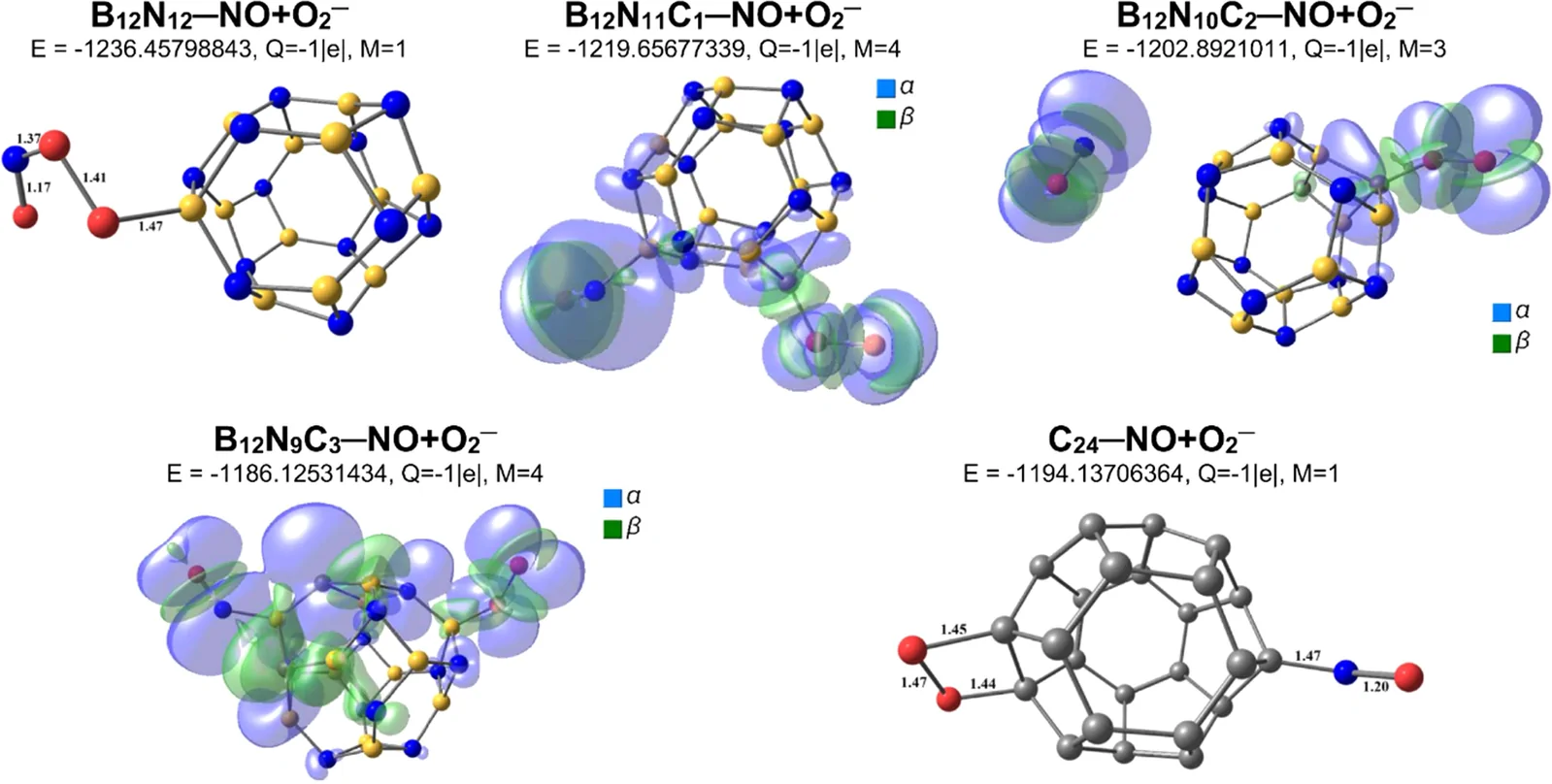
Graphical representation of optimized BNFs–NO/O_2_^–^ coadsorption complexes: ball-and-stick structures, total electronic energy (*E*, Hartree), charge (*Q*), and spin multiplicity (*M*), along with spin density isosurfaces for open-shell systems (*M* > 1). B, N, C, and O atoms are shown in yellow, blue, gray, and red, respectively. Positive (blue) and negative (green) regions denote excess α- and β-spin densities, highlighting polarization effects upon coadsorption.

**Table 7:** Adsorption Energy (*E*_ad_; eV), HOMO–LUMO Gap (HL_gap_; eV), Dipole Moment (DM; Debye), Chemical Potential (μ; eV), Work Function (WF; eV), Polarizability (α; a. u.), Global Hardness (η; eV), Global Softness (*S*; eV^–1^), and Electrophilicity (ω; eV) in Gas and Aqueous Phases for BNFs + NO + O_2_^–^ in Anionic State^a^

structure	B_12_N_12_–NO + O_2_^–^		B_12_N_11_C_1_–NO + O_2_^–^		B_12_N_10_C_2_–NO + O_2_^–^		B_12_N_9_C_3_–NO + O_2_^–^		C_24_–NO + O_2_^–^	
quantum descriptor	gas	water	gas	water	gas	water	gas	water	gas	water
*E*_ad, NO_	–1.38	–1.28	–4.16	–2.97	–0.10	–0.08	–4.41	–4.19	–1.72	–1.68
*E*_ad, O2–_	–6.01	–4.33	–6.89	–5.24	–5.19	–4.78	–6.45	–4.70	–5.69	–4.06
HL_gap_	9.05	8.95	7.57	7.83	6.93	7.37	6.58	6.84	5.58	5.66
DM	3.76	5.72	5.61	8.11	5.73	8.16	6.09	9.91	1.66	3.14
μ	–1.35	–4.57	–1.33	–4.43	–1.53	–4.52	–2.11	–5.05	–1.21	–4.19
WF	4.53	4.47	3.79	3.92	3.47	3.69	3.29	3.42	2.79	2.83
α	191.33	267.46	199.88	281.87	207.56	299.38	227.07	333.58	252.98	415.01
η	4.53	4.47	3.79	3.92	3.47	3.69	3.29	3.42	2.79	2.83
*S*	0.11	0.11	0.13	0.13	0.14	0.14	0.15	0.15	0.18	0.18
ω	0.20	2.33	0.23	2.51	0.34	2.77	0.67	3.73	0.26	3.10

a Reported at the ωB97X-D/6-311++G­(d,p) level.

As shown in Figure 6, the coadsorption of NO and O_2_^–^ on the B_12_N_12_ surface promotes the stabilization of the ONOO^–^. The coadsorbed configuration enables their coupling, leading to the formation of a bound peroxynitrite-like intermediate. For B_12_N_12_ in the gas phase, the ONOO-like product was confirmed as a true minimum, and a first-order TS was located. Forward and reverse IRC calculations were performed from this TS, with the full details provided in the Section S6. The forward IRC evolves toward an ONOO-like geometry with *d*_N–O_ = 1.381 Å, which is very close to the optimized product value of 1.375 Å. The reverse IRC elongates the N···O contact to 2.636 Å, consistent with the precursor side of the reaction coordinate. The dissociation barrier from the ONOO-like product to the TS is Δ*E* ^‡^ = 2.83 eV and Δ*G* ^‡^ = 2.74 eV, indicating that the B_12_N_12_-supported ONOO-like adduct is highly stable toward dissociation. The approximate barrier from the reverse IRC end point to the TS is 0.52 eV. This is used qualitatively because the reverse end point was not optimized as an independent minimum. The structural characteristics are consistent with available experimental data for peroxynitrite/peroxonitrite. The calculated O­(NO)–N­(NO), N­(NO)–O­(O_2_), and O–O bond distances of 1.173, 1.375, and 1.407 Å, respectively, closely match crystallographic values reported for the peroxynitrite anion, approximately 1.16, 1.35, and 1.41 Å.^77^ In PCM water, a candidate first-order saddle point with one imaginary frequency of −89.30 cm^–1^ and an N–O distance of 2.369 Å was located. Because bidirectional IRC connectivity was not established for the aqueous model, this structure is treated as provisional rather than as a validated reaction pathway. Its relative Δ*E* ^‡^ and Δ*G* ^‡^ with respect to the aqueous ONOO-like minimum are 2.07 and 1.98 eV, respectively.

Stabilization is supported by the *E*_ad_ associated with O_2_^–^ on pristine BNF (*E*_ad_ = −6.01 eV in the gas phase and −4.33 eV in water). The strong initial chemisorption of O_2_^–^ on the pristine BNF acts as an anchor for this reaction. This anchoring effect is indicative of the intrinsic role of the polar B–N bonds, which provide electrostatic stabilization for the newly formed anionic ONOO^–^ species, even in the absence of C-doping. Pristine B_12_N_12_ is better described as a surface-mediated radical-trapping platform that stabilizes the ONOO-like adduct through strong electrostatic and covalent interactions. B_12_N_12_ promotes and stabilizes ONOO-like formation under the explored conditions; this role is closer to irreversible or persistent radical sequestration than to a fully regenerable catalytic cycle.

Local structural rearrangements are exhibited by C-doped BNFs upon adsorption, primarily in B_12_N_11_C_1_ and B_12_N_9_C_3_, where NO binds directly to B-adjacent sites. The simultaneous coadsorption of NO and O_2_^–^ species induces complex spin polarization across the B_12_N_12–*x*_C_*x*_ hybrid complexes. The resulting spatial distribution of positive (blue) and negative (green) spin densities highlights the broad polarization driven by the dual interaction of the analytes with the BNF (Figure 6). C-substitution introduces localized electronic states that act as preferential adsorption centers. The interactions of O_2_^–^ over BNFs surfaces are stronger (chemisorptive) than for NO, consistent with the higher charge density and anionic character of O_2_^–^.

B_12_N_10_C_2_ displays strong coadsorption of O_2_^–^ (−5.19 and −4.78 eV in gas and water), whereas NO remains weakly bound (−0.10 and −0.08 eV). This selectivity reflects preferential stabilization of O_2_^–^ at the dominant reactive site. The large exothermicity supports persistent and potentially irreversible trapping within the modeled conditions but does not establish irreversibility in physiological media. B_12_N_10_C_2_ is therefore better described as a selective O_2_^–^-trapping candidate than as an intrinsically reusable sensor. Interaction between NO and the BNF is predominantly physisorptive, involving minimal CT or orbital hybridization (see Table S4 in the Supporting Information for topological analyses). This selective chemisorption of O_2_^–^ isolates NO from direct interaction with O_2_^–^, suppressing their coupling and hindering the formation of reactive N–O species such as ONOO^–^. Strong NO capture requires an accessible, localized defect state with favorable C–N orbital overlap and spin matching. In B_12_N_10_C_2_, the defect distribution stabilizes the anionic O_2_^–^ more efficiently than the neutral NO. This results in strong O_2_^–^ chemisorption and weak NO binding. From a mechanistic perspective, B_12_N_10_C_2_ acts as a potential NO-protective platform, where the BNF scavenges O_2_^–^ while maintaining NO in a quasi-free state. This is advantageous in applications requiring the preservation of NO bioavailability or controlled NO signaling, while simultaneously mitigating oxidative pathways driven by O_2_^–^. C_24_ displays high polarizability and a relatively small HL_gap_. Its adsorption performance remains inferior to that of C-doped BNFs, particularly for NO. The heteroatomic composition is relevant due to the intrinsic polarity of the B–N bonds, combined with the localized C states.

The Fukui analysis of B_12_N_9_C_3_ shows a distributed, C-centered reactivity pattern. C18 is the primary NO-active site, with *f*_0_ values of 0.229 in gas and 0.310 in water. In contrast, C20 dominates the O_2_^–^ response with *f*_0_ values of 0.191 and 0.258, respectively. This separation of active centers supports the proposed dual-scavenging role of B_12_N_9_C_3_. The tridoped BNF provides multiple chemically accessible C-centered regions that enable NO and O_2_^–^ to interact through distinct yet complementary local reactivity patterns. This complements the NBO results, which show adsorbate- and medium-dependent charge redistribution in the coadsorbed complexes. The NBO analysis reveals adsorbate- and medium-dependent charge redistribution involving both species (see Sections S4 and 5 for more details). While this supports strong radical stabilization, it does not prove regenerability. B_12_N_9_C_3_ is therefore better described as a highly reactive dual-capture platform, although O_2_^–^ binding can induce local BNF reconstruction.

C-doping decreases the HL_gap_ and makes the BNFs softer relative to pristine B_12_N_12_. The adsorption strength does not depend strictly monotonically on the number of C dopants. Radical binding is governed by a coupled global-local effect. Global descriptors, such as the HL_gap_, hardness, and softness, indicate the BNF’s electronic susceptibility. Local factors, such as defect geometry, spin localization, Fukui reactivity, and NBO charge redistribution, determine if a radical can form effective B–O, C–O, or C–N interactions. This explains why B_12_N_9_C_3_ can provide a favorable dual compromise for NO and O_2_^–^ adsorption while B_12_N_11_C_1_ can be particularly effective at capturing isolated O_2_^–^ despite having a lower C-doping content.

Controlled C-doping in BNFs enables the tuning of adsorption strength, electronic reactivity, and solvent stability. These characteristics position C-doped BNFs as promising materials for gas sensing and electrochemical applications involving ROS/RNS species.

### 3.4 Thermal Stability of BNFs–NO and BNFs–O_2_^–^ Complexes

While adsorption energies confirm the feasibility of radical capture, practical efficacy depends on kinetic retention. To address this, the thermal stability of the BNFs–NO and BNFs–O_2_^–^ complexes was assessed using 1D-PES analyses combined with Langevin dynamics. By linking electronic structure descriptors to effective electronic escape threshold and temperature-dependent lifetimes, this approach translates microscopic parameters into macroscopic stability metrics in both vacuum and aqueous media.

The relaxed PES shown in Figure 7 were used to determine the topology of the desorption pathways prior to assigning any kinetic parameters. Most chemically continuous profiles were characterized by a monotonic increase from the adsorbed minimum toward a nearly constant dissociation plateau, rather than by an internal stationary maximum. The plateau height was therefore defined as an effective electronic escape threshold (Δ*E*_eff_) and was not interpreted as a stationary-point activation free energy. The conventional desorption TS was not accepted because the requirement of one significant desorption-aligned imaginary frequency and bidirectional IRC connectivity between the adsorbed and dissociated states was not satisfied. This treatment agrees with the behavior described by Quapp, Kraka, and Cremer for quasi-flat dissociation pathways, where either a distant loose saddle point or a genuinely barrierless multidimensional pathway may be present.^78^ The mathematical nonexistence of a saddle point over the complete multidimensional PES was not claimed; rather, no TS suitable for conventional TST was validated under the applied search and connectivity criteria.

**Figure 7:**
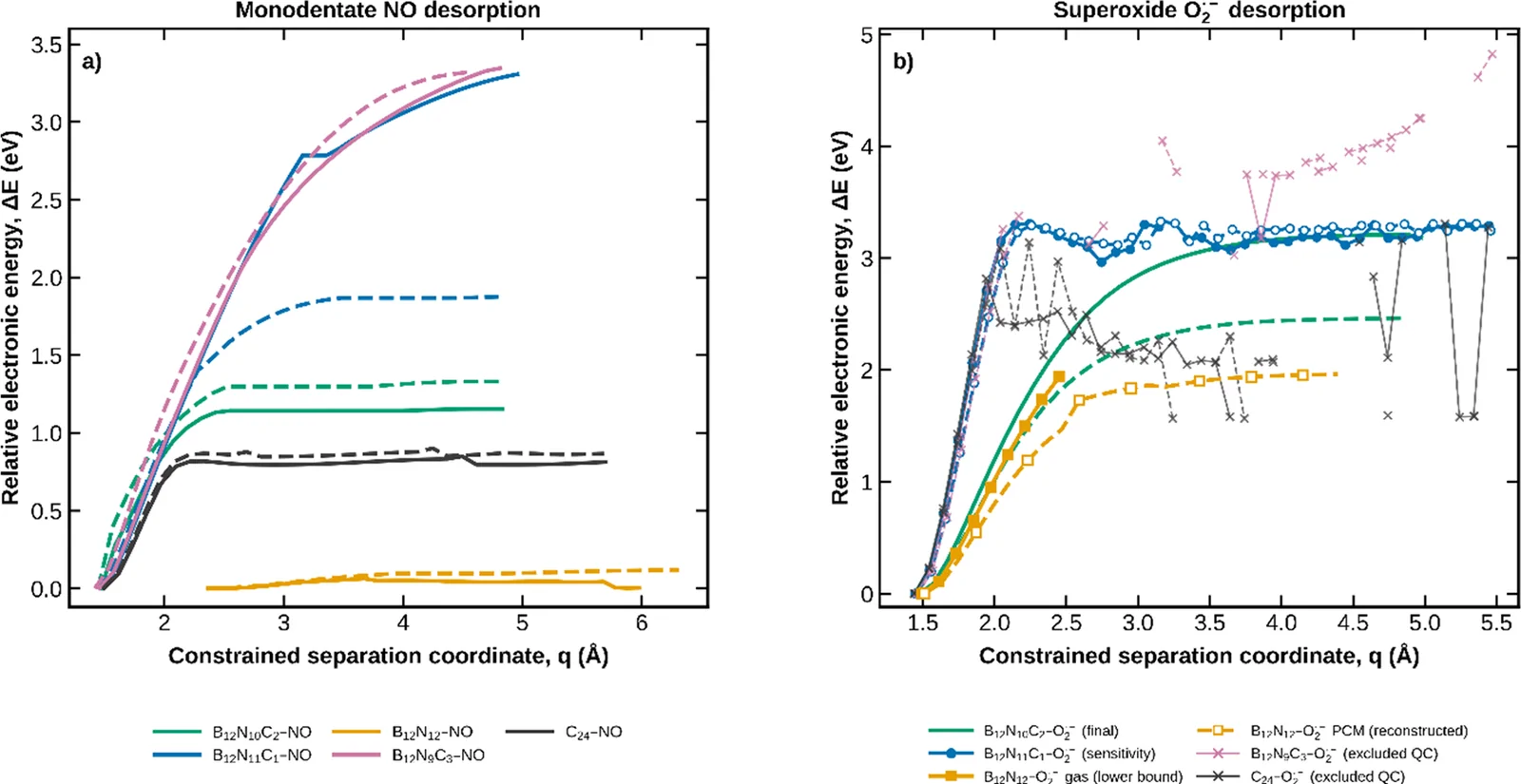
Relaxed PES for NO and O_2_^–^ desorption from C_24_ and B_12_N_12–*x*_C_*x*_ fullerenes (*x* = 0–3). (a) Monodentate NO desorption profiles. (b) O_2_^–^ desorption profiles. Solid and dashed lines represent gas-phase and PCM-water calculations, respectively. For B_12_N_12_–O_2_^–^, the gas-phase profile is reported as a 1.938 eV lower-bound branch ending before reanchoring, whereas the PCM-water profile corresponds to a cleaned reconstructed plateau of 1.961 eV. B_12_N_9_C_3_–O_2_^–^ and C_24_–O_2_^–^ fragments are shown only as excluded points that did not satisfy the structural and electronic consistency criteria. Relative electronic energies were calculated at the ωB97X-D/6-311++G­(d,p) level. The predominantly monotonic, plateau-like profiles define effective escape thresholds rather than formal transition-state barriers.

C-substitution produced a wide range of NO-retention profiles. The weakest surface was obtained for pristine B_12_N_12_–NO, for which effective thresholds of only 0.062 and 0.119 eV were obtained in the gas phase and PCM water, respectively. The small plateau heights were accompanied by the long B–N equilibrium separation of 2.48 Å and were indicative of a shallow adsorption well. Shorter C–N contacts of approximately 1.42–1.49 Å and markedly higher plateaus were obtained after C-substitution. These findings are consistent with earlier DFT studies, which demonstrated that NO adsorption is highly influenced by the chemical composition of the BNF, as well as the local electronic structure of BN and C-doped BNFs. An intermediary NO-desorption regime was obtained for C_24_ and B_12_N_10_C_2_. The effective thresholds of 0.850–0.901 eV were obtained for C_24_–NO, whereas values of 1.154 and 1.331 eV were obtained for B_12_N_10_C_2_–NO in gas and PCM water, respectively. According to the findings, the highest NO plateaus were obtained for B_12_N_11_C_1_ and B_12_N_9_C_3_, with values ranging from 1.875 to 3.346 eV. These latter profiles were assigned provisional or reconstructed evidence classes because root switching, branch truncation, or absolute-energy reconstruction was involved. The numerical values were therefore retained as evidence for high-retention regimes, but they were not assigned the same confidence as the final continuous C_24_ and primary B_12_N_10_C_2_ profiles. The complete NO data set and its evidence classification are summarized in Table 8. Retention was dictated by the specific defect arrangements and adsorption sites. Single and triple substitution produced the largest effective thresholds, whereas double substitution produced an intermediate profile. Similar sensitivity of BNF reactivity to the position and electronic character of C-substitution has been reported for reactions catalyzed by C-doped BNFs. This supports the interpretation that C acts as a local electronic defect rather than as a simple additive compositional variable.^79^

**Table 8:** Structural, Energetic, and Kinetic Descriptors for NO and O_2_^–^ Desorption from C_24_ and B_12_N_12–*x*_C_*x*_ Fullerenes (*x* = 0–3) in the Gas Phase and PCM Water^a^

complex	phase	bond	μ_eff_ (amu)	*q*_min_ (Å)	Δ*E*_eff_ (eV)	τ_MFPT_, 298.15 K	*P*_des_ 298.15 K, 100 ps	*P*_des_ 2000 K, 100 ps	evidence classification
NO Complexes									
B_12_N_12_–NO	gas	B–N	6.10	2.48	0.062	12.49 ps	1.000	1.000	final continuous PES
B_12_N_12_–NO	PCM water	B–N	6.10	2.35	0.119	106.23 ps	0.546	1.000	final continuous PES
B_12_N_11_C_1_–NO	gas	C–N	6.47	1.46	3.307	2.180 × 10^34^ yr	0.000	0.000	final reconstructed PES
B_12_N_11_C_1_–NO	PCM water	C–N	6.47	1.44	1.875	9.837 × 10^10^ yr	0.000	0.040	final reconstructed PES
B_12_N_10_C_2_–NO	gas	C–N	6.47	1.47	1.154	18.47 d	0.000	0.878	final continuous PES
B_12_N_10_C_2_–NO	PCM water	C–N	6.47	1.44	1.331	26.14 yr	0.000	0.706	final continuous PES
B_12_N_9_C_3_–NO	gas	C–N	6.47	1.45	3.346	8.911 × 10^34^ yr	0.000	0.000	final reconstructed PES
B_12_N_9_C_3_–NO	PCM water	C–N	6.47	1.42	3.320	4.106 × 10^34^ yr	0.000	0.000	final reconstructed PES
C_24_–NO	gas	C–N	6.47	1.51	0.850	9.893 s	0.000	1.000	final continuous PES
C_24_–NO	PCM water	C–N	6.47	1.49	0.901	74.97 s	0.000	0.990	final continuous PES
Superoxide Complexes									
B_12_N_12_–O_2_^–^	gas	B–O	6.45	1.49	1.938^b^	1.342 × 10^10^ yr^b^	—	—	sensitivity-only lower-bound PES^b^
B_12_N_12_–O_2_^–^	PCM water	B–O	6.45	1.51	1.961^c^	1.179 × 10^12^ yr^c^	—	—	provisional reconstructed PES^c^
B_12_N_11_C_1_–O_2_^–^	gas	C–O	6.86	1.43	3.282 ± 0.004^d^	—^d^	0.000	0.000	sensitivity-only dual-coordinate PES^d^
B_12_N_11_C_1_–O_2_^–^	PCM water	B–O	6.45	1.48	3.282 ± 0.034^d^	—^d^	0.000	0.000	sensitivity-only dual-coordinate PES^d^
B_12_N_10_C_2_–O_2_^–^	gas	B–O	6.45	1.47	3.211	1.832 × 10^33^ yr	0.000	0.000	final continuous bidentate PES
B_12_N_10_C_2_–O_2_^–^	PCM water	B–O	6.45	1.49	2.462	4.253 × 10^20^ yr	0.000	0.000	final continuous bidentate PES
B_12_N_9_C_3_–O_2_^–^	gas	C–O	6.86	1.42	—^e^	—^e^	—	—	excluded: reanchoring/contact switching^e^
B_12_N_9_C_3_–O_2_^–^	PCM water	B–O	6.45	1.43	—^e^	—^e^	—	—	excluded: reanchoring/contact switching^e^
C_24_–O_2_^–^	gas	C–O	6.86	1.44	—^e^	—^e^	—	—	excluded: reanchoring/O–O identity loss^e^
C_24_–O_2_^–^	PCM water	C–O	6.86	1.44	—^e^	—^e^	—	—	excluded: reanchoring/O–Oidentity loss^e^

a **Notes.** μ_eff_ is the reduced mass of the atom pair defining the BNF-adsorbate contact; *q*_min_ is the equilibrium separation of that contact. Δ*E*_eff_ = *U*(*q*_abs_) – *U*(*q*_min_). The τ_MFPT_ values are PES-based mean first-passage-time projections and are not formal TST lifetimes. *P*_des_ values are direct rupture fractions from 500 independent 100 ps Langevin trajectories wherever such trajectories were available. A value of 0.000 denotes a right-censored zero-event result; for 0/500 events, the 95% Wilson upper limit is 0.00762. An em dash indicates that no quantitative descriptor was assigned or that a direct trajectory result was unavailable.

b B_12_N_12_–O_2_^–^ gas: lower-bound monodentate branch ending at the last chemically continuous point before reanchoring; no direct 100 ps Langevin data set was available for this updated branch.

c B_12_N_12_–O_2_^–^ PCM water: cleaned reconstructed plateau obtained after incomplete and branch-switching windows were removed; no direct 100 ps Langevin data set was available for this updated branch.

d B_12_N_11_C_1_–O_2_^–^: corrected dual-coordinate plateau retained as sensitivity evidence only. O–O contraction indicates adiabatic redox-coupled reorganization. No quantitative MFPT was assigned; the direct probabilities correspond only to the conservative sensitivity model used in the completed Langevin calculations.

e No effective escape threshold, MFPT, or rupture probability was assigned because reanchoring, contact switching, or loss of O–O identity invalidated the one-dimensional desorption branch.

A restrictive interpretation was necessary for the O_2_^–^ profiles because separation could be accompanied by asymmetric contact breaking, reanchoring, O–O contraction, or a change in molecular identity. B_12_N_10_C_2_–O_2_^–^ demonstrated the strongest final and chemically continuous O_2_^–^ profiles, with effective thresholds of 3.211 eV in gas and 2.462 eV in PCM water. The surfaces retained the intended branch, making them suitable for use as the principal quantitative O_2_^–^ models. B_12_N_12_–O_2_^–^ scans were identified as monodentate B–O separation profiles. A lower-bound threshold of 1.938 eV was obtained from the last chemically continuous point before reanchoring, whereas a cleaned reconstructed PCM plateau of 1.961 eV was obtained after removing incomplete and branch-switching windows. These profiles were used to extend the O_2_^–^ comparison, but they remained classified as sensitivity-only and provisional, respectively. Corrected dual-coordinate plateaus of 3.282 ± 0.004 eV in gas and 3.282 ± 0.034 eV in PCM water were recovered for B_12_N_11_C_1_–O_2_^–^. This close agreement between the two phases confirms a high energetic scale for separation. Contraction of the O–O distance from approximately 1.47 to 1.19 Å indicates that substantial electronic and structural reorganization occurred along the selected branch. The profiles were retained solely as evidence of energetic sensitivity and were not considered fixed-identity O_2_^–^ desorption pathways. No final threshold was assigned to B_12_N_9_C_3_–O_2_^–^ or C_24_–O_2_^–^ because reanchoring, contact switching, or loss of O–O identity prevented a unique 1D desorption coordinate from being preserved.

The isolated C-doped cages retained their closed topology throughout the sampled 300 K ADMP windows, with no cage-opening or bond-breaking events and RMSD values ≤0.107 Å. The radical-bound trajectories reveal distinct composition- and medium-dependent finite-temperature behavior. Pristine B_12_N_12_–NO undergoes rapid molecular NO desorption in both gas-phase and PCM-water trajectories while preserving the integrity of the 24-atom cage, consistent with its shallow adsorption well and ultralabile first-passage behavior. In contrast, molecular O_2_^–^ remains adsorbed on B_12_N_10_C_2_ in the single-adsorbate trajectories without loss of O–O identity. B_12_N_11_C_1_–O_2_^–^ undergoes rapid O–O dissociation accompanied by defect-localized reconstruction. For B_12_N_9_C_3_–O_2_^–^, early reanchoring and local cage reconstruction are followed by O–O dissociation and reactive trapping in both gas and PCM-water trajectories. Thus, strong adsorption does not necessarily imply preservation of the original molecular adsorption motif. The B_12_N_10_C_2_–NO/O_2_^–^ coadsorption trajectories show a medium-dependent response: in the gas phase, NO separates from the cage while molecular O_2_^–^ remains adsorbed, whereas in PCM water NO also separates but the O–O bond subsequently enters a dissociative regime accompanied by local cage reconstruction and reactive oxygen trapping. The B_12_N_9_C_3_ gas-phase coadsorption trajectory retains both adsorbates while undergoing persistent defect-region reconstruction; the corresponding PCM-water trajectory also retains both adsorbates over the analyzed contiguous observation window. The preformed B_12_N_12_ coadsorption trajectories preserve a surface-bound ONOO^–^-like product motif in both gas and PCM water. In addition, a gas-phase trajectory initiated from the precursor-like reverse-IRC end point shows spontaneous transient ONOO^–^-like coupling through N26–O28 bond formation, reaching a minimum N26–O28 separation of 1.396 Å at approximately 0.475 ps while the cage remains structurally preserved. This dynamically accessed connectivity differs from the N26–O27 static IRC pathway and demonstrates an alternative finite-temperature oxygen-switching coordinate on the B_12_N_12_ surface.

As shown in Figure 8, the temperature-dependent PES-MFPT lifetimes translate the energetic hierarchy of the PES into distinct kinetic-retention regimes. For each accepted branch, lifetimes were calculated from the corresponding audited potential profile. The lifetimes thus incorporated not only the terminal energy difference but also the width and shape of the escape region. This treatment follows the diffusion-controlled escape framework introduced by Kramers and subsequently generalized for friction-dependent reaction dynamics and first-passage processes.^55^ The Langevin analysis provides evidence for kinetic retention along the modeled desorption coordinate. This analysis does not represent full biological conditions. High-temperature simulations were used to expedite the analysis of rare events and derive kinetic trends, rather than to replicate experimental conditions.

**Figure 8:**
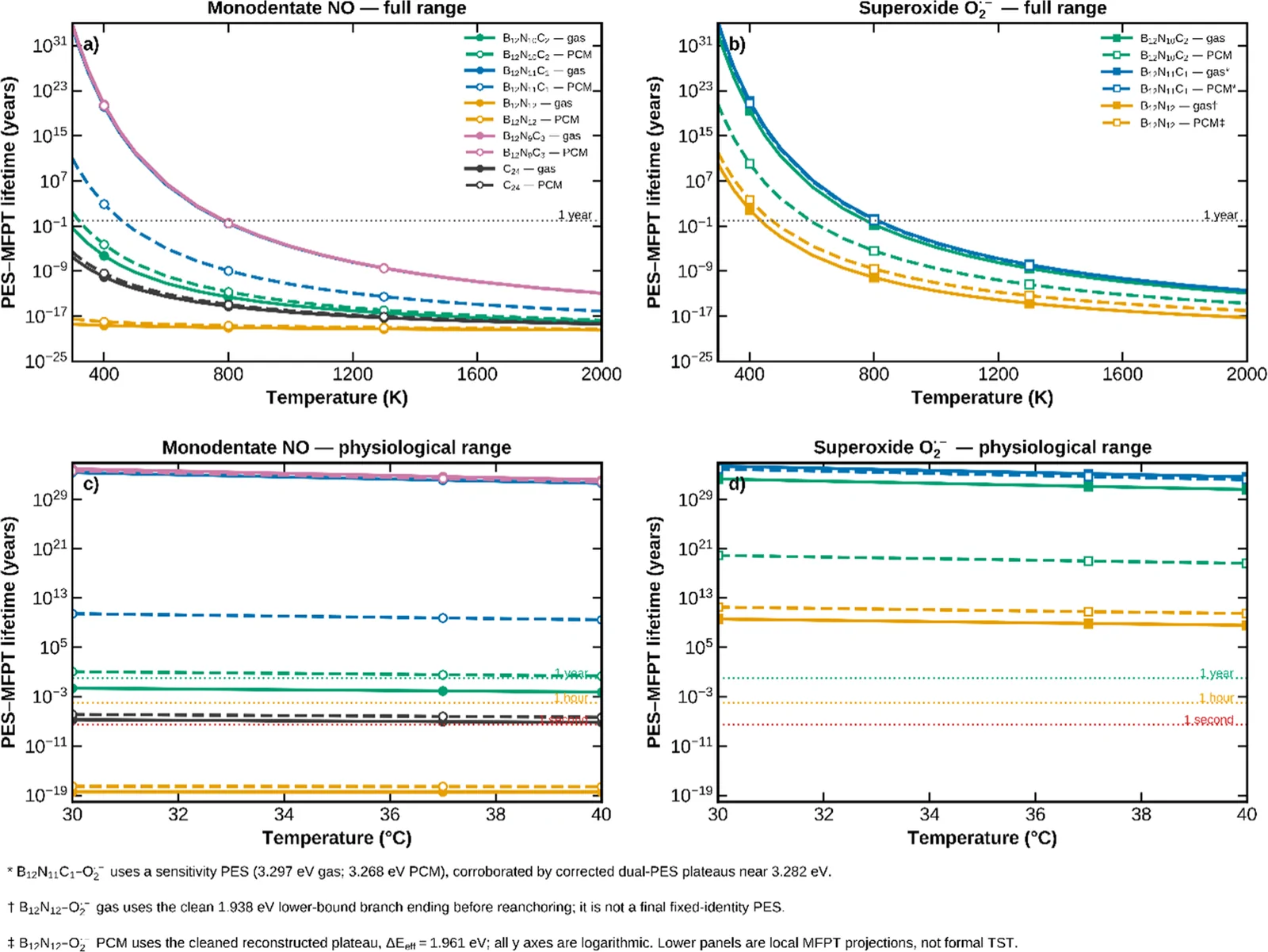
Temperature-dependent PES-MFPT desorption lifetimes for NO and O_2_^–^ on C_24_ and B_12_N_12–*x*_C_*x*_ fullerenes (*x* = 0–3). Panels (a,b) show the projected desorption lifetimes over the full temperature range of 298.15–2000 K, whereas panels (c,d) show the corresponding local projections over the physiological range of 30–40 °C. Solid and dashed curves represent gas-phase and PCM-water results, respectively. The horizontal dotted line in the upper panels denotes a one-year lifetime, while the lower panels additionally include 1 h and one-second reference thresholds. Lifetimes were obtained from mean first-passage-time calculations on the ωB97X-D/6-311++G­(d,p) PES and therefore represent PES-MFPT projections rather than formal TST lifetimes or direct long-time trajectories. The B_12_N_11_C_1_–O_2_^–^ curves are included as sensitivity models. For B_12_N_12_–O_2_^–^, the gas-phase curve is based on the 1.938 eV lower-bound branch before reanchoring, whereas the PCM-water curve uses the cleaned reconstructed plateau of 1.961 eV.

The selected temperatures reflect the high thermal stability of BNFs while remaining below their thermodynamic limits. C sublimates near 3900 K and BN frameworks decompose at approximately 3000–3300 K. Temperatures above the near-ambient range were used exclusively for accelerated computational stress testing, which allowed rare escape events to be simulated and compared with kinetic-retention trends. These temperatures are not proposed operating temperatures and do not establish the thermal integrity of the complete BNF. Practical interpretation is limited to the modeled near-ambient range of approximately 298.15–313.15 K. The higher-temperature results are only used for comparative mechanistic analysis.

The resulting PES-MFPT lifetimes classified the complexes into different retention regimes. The complete structural, energetic, and kinetic values are reported in Table 8. Pristine B_12_N_12_–NO was classified as ultralabile; C_24_–NO exhibited reversible retention within seconds to minutes; B_12_N_10_C_2_–NO occupied an intermediate regime; and B_12_N_11_C_1_–NO and B_12_N_9_C_3_–NO were classified as having persistent retention, though their branch classifications were provisional or reconstructed. The strongest final quantitative evidence for O_2_^–^ was obtained for B_12_N_10_C_2_–O_2_^–^. B_12_N_12_–O_2_^–^ was represented by lower-bound and reconstructed profiles. B_12_N_11_C_1_–O_2_^–^ was retained only as sensitivity evidence. No quantitative threshold was assigned to the reanchored B_12_N_9_C_3_–O_2_^–^ and C_24_–O_2_^–^ branches. These classifications are consistent with the branch-preservation hierarchy established from the PES analysis. Several systems demonstrate lifetimes that are projected to occupy persistent-retention regimes at 298–313 K, subject to the sensitivity of the reconstructed PES.

The temperature range over which functional stability is retained differs among materials, emphasizing the critical role of C content and bonding motifs. Systems such as B_12_N_11_C_1_ and B_12_N_9_C_3_ are well-suited for permanently capturing and stabilizing NO because their large effective electronic escape threshold ensures long-lived adsorption, even under solvated conditions. This is consistent with the strong chemisorptive character observed in static DFT analysis. B_12_N_10_C_2_ has a unique thermal signature. O_2_^–^ is stabilized with extended lifetimes, but NO remains weakly bound and thermally labile. This selective stability creates a regime where O_2_^–^ is efficiently trapped while NO remains mobile and available, protecting NO. This behavior is important for applications requiring the preservation of NO activity while blocking oxidative pathways driven by O_2_^–^.

Direct Langevin probabilities and sensitivity analyses, as shown in Figure 9, were found to reproduce the kinetic ordering derived from the PES-MFPT curves and summarized in Table 8. Rupture was directly observed within 100 ps at 298.15 K for the weakest NO complex, B_12_N_12_–NO, with probabilities of 1.000 in the gas phase and 0.546 in PCM water. No room-temperature rupture was detected for the remaining NO systems within the same observation window. At 2000 K, thermally activated rupture entered the direct simulation window for the intermediate-retention systems. Probabilities reached 1.000 and 0.990 for C_24_–NO and 0.878 and 0.706 for B_12_N_10_C_2_–NO in the gas phase and in PCM water, respectively. B_12_N_11_C_1_–NO and B_12_N_9_C_3_–NO remained predominantly right-censored, except for a probability of 0.040 for B_12_N_11_C_1_–NO in PCM water.

**Figure 9:**
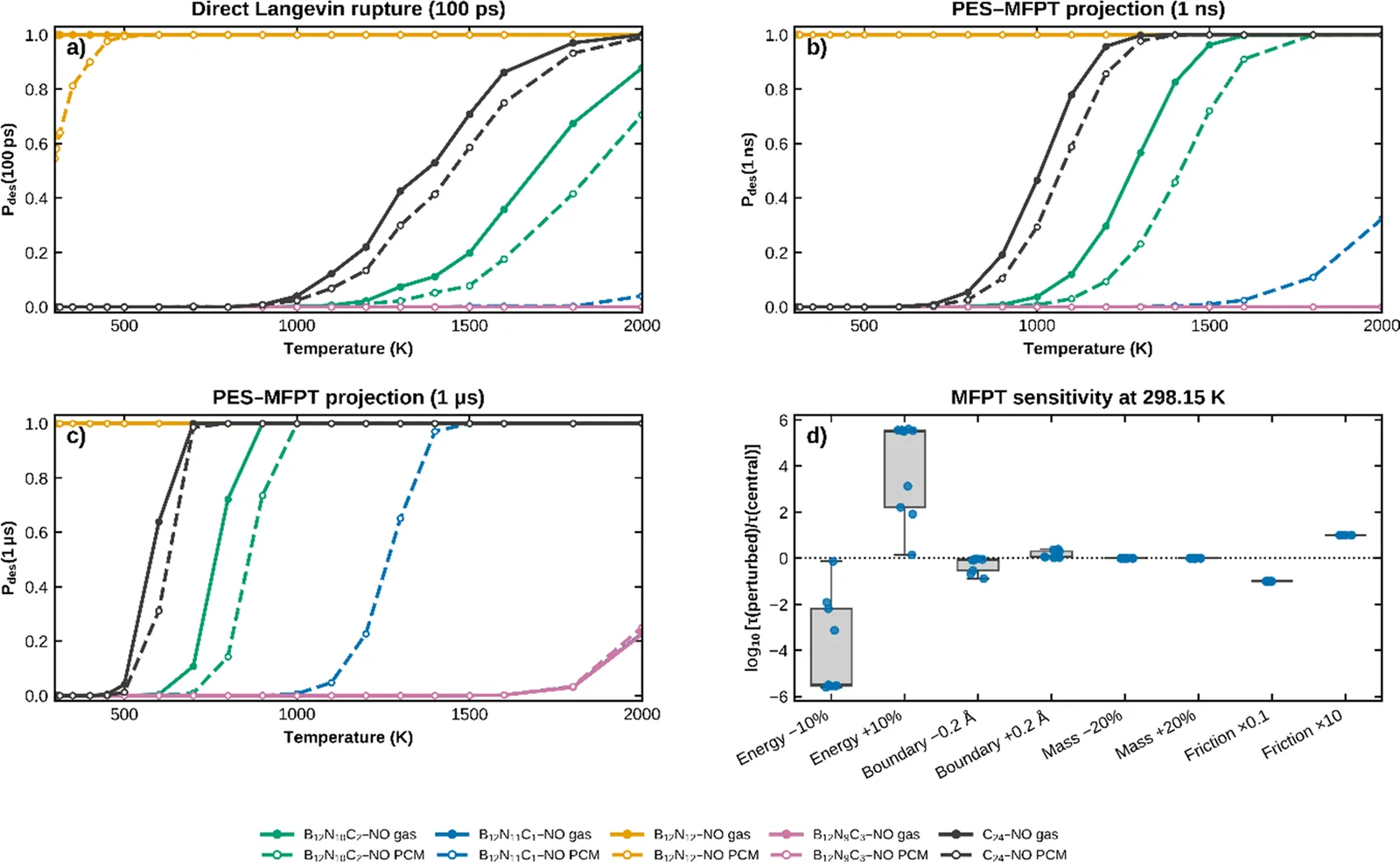
Direct Langevin rupture probabilities, PES–MFPT time projections, and model sensitivity for NO desorption from C_24_ and B_12_N_12‑x_C_*x*_ fullerenes (*x* = 0–3). (a) Direct first-passage rupture probability within 100 ps, obtained from 500 independent Langevin trajectories at each temperature. (b,c) Probabilities at 1 ns, 1 μs, and longer times were obtained from the PES–MFPT projections using *P*_des_(*t*;*T*) = 1 – exp­[−*t*/τ­(*T*)]. These panels are MFPT-based projections rather than explicit trajectories of the corresponding duration. Solid and dashed curves denote gas-phase and PCM-water results, respectively. (d) Sensitivity of the 298.15 K MFPT to ±10% PES-energy scaling, ±0.2 Å displacement of the absorbing boundary, ±20% effective-mass variation, and 10-fold changes in friction. The ordinate reports *log*_10_[τ_perturbed_/τ_central_]; positive and negative values indicate increased and decreased lifetimes relative to the central model, respectively. Energy perturbations dominate the uncertainty, whereas mass has negligible influence in the overdamped MFPT treatment and friction produces an approximately linear response.

For the NO branches, the direct 100 ps rupture probabilities were qualitatively consistent with the retention ordering obtained from the PES-MFPT projections. Stable stochastic propagation was provided by the BAOAB integration scheme. Sensitivity analysis showed that electronic PES was the dominant source of uncertainty. A ± 10% energy perturbation changed the low-temperature MFPT by factors ranging from 3 × 10^–6^ to 4 × 10^5^, whereas displacement of the absorbing boundary by ±0.2 Å produced smaller changes of 0.132–2.42. Overdamped MFPT was modified by friction approximately linearly, while variation of the reduced mass had no effect on the MFPT integral. These results are consistent with the narrow 6.10–6.86 amu range listed in Table 8. Combined structural, energetic, and kinetic evidence established the NO-retention order B_12_N_12_ < C_24_ < B_12_N_10_C_2_ ≪ B_12_N_11_C_1_ ≈ B_12_N_9_C_3_, corresponding to ultrafast release, reversible retention in the seconds-to-minutes range, intermediate storage, and kinetically persistent sequestration, respectively. For O_2_^–^, the strongest final quantitative support for persistent capture was obtained for B_12_N_10_C_2_. B_12_N_12_ was represented by lower-bound and reconstructed models; B_12_N_11_C_1_ was represented by sensitivity evidence; and B_12_N_9_C_3_ and C_24_ were excluded because a continuous fixed-identity branch was not preserved. The preferred BNF depended on the intended application: weak/rapid-release was favored by B_12_N_12_; intermediate or potentially reversible retention by C_24_; intermediate storage, by B_12_N_10_C_2_; and persistent retention within the model, by B_12_N_11_C_1_ or B_12_N_9_C_3_. Strong O_2_^–^ capture was most convincingly supported by B_12_N_10_C_2_. Strong retention should not be equated with regenerability because the high escape threshold that favors sequestration may hinder radical release and active-site recovery.

Thermodynamic viability of the radical scavenging processes was assessed at standard conditions (298 K and 1 atm of pressure) by computing the Gibbs free energies of adsorption (Δ*G*_ad_) in both gas and aqueous phases (see Table 9). Incorporating entropic and thermal corrections indicates a crucial limitation of the pristine BNF: the interaction between B_12_N_12_ and NO yields positive values for the Δ*G*_ad_ (+18.27 and +24.55 kJ mol^–1^ in gas and water, respectively). This indicates that the entropic penalty of association overcomes the weak vdW forces, rendering the physisorption process nonspontaneous at room temperature.

**Table 9:** Thermodynamic Adsorption Parameters for BNF–NO and BNFs–O_2_^–^ Complexes in the Gas Phase and Continuum Water^a^

complex	phase	Δ*H*_ad_ (kJ mol^–1^)	Δ*S*_ad_ (kJ mol^–1^ K^–1^)	Δ*G*_ad_ (kJ mol^–1^)	*T*_eq_ (K)
B_12_N_12_–NO	gas	–7.52	–0.09	18.27	–
B_12_N_12_–NO	water	–11.25	–0.12	24.55	–
B_12_N_11_C_1_–NO	gas	–172.54	–0.16	–125.04	1082.91
B_12_N_11_C_1_–NO	water	–179.72	–0.16	–132.00	1122.91
B_12_N_10_C_2_–NO	gas	–95.15	–0.14	–54.19	692.58
B_12_N_10_C_2_–NO	water	–99.08	–0.14	–57.54	711.11
B_12_N_9_C_3_–NO	gas	–167.28	–0.16	–119.39	1041.51
B_12_N_9_C_3_–NO	water	–188.74	–0.16	–140.62	1169.24
C_24_–NO	gas	–79.68	–0.14	–37.66	565.36
C_24_–NO	water	–83.78	–0.14	–41.39	589.27
B_12_N_12_–O_2_^–^	gas	–322.31	–0.14	–279.33	2235.87
B_12_N_12_–O_2_^–^	water	–185.91	–0.14	–143.05	1293.32
B_12_N_11_C_1_–O_2_^–^	gas	–444.83	–0.17	–395.42	2684.27
B_12_N_11_C_1_–O_2_^–^	water	–312.93	–0.17	–262.43	1847.34
B_12_N_10_C_2_–O_2_^–^	gas	–368.27	–0.14	–327.72	2707.94
B_12_N_10_C_2_–O_2_^–^	water	–232.05	–0.14	–191.19	1693.31
B_12_N_9_C_3_–O_2_^–^	gas	–380.78	–0.17	–331.41	2299.24
B_12_N_9_C_3_–O_2_^–^	water	–246.52	–0.17	–196.59	1472.21
C_24_–O_2_^–^	gas	–380.75	–0.16	–332.43	2349.23
C_24_–O_2_^–^	water	–232.55	–0.17	–181.80	1366.18

a The Van’t Hoff representation assumes temperature-independent Δ*H*_ad_ and Δ*S*_ad_ over 298.15–700 K. *T*_eq_ values outside this interval are reported only as qualitative extrapolations.

The introduction of C defects serves as a thermodynamic switch. C-doped systems, notably B_12_N_11_C_1_ and B_12_N_9_C_3_, demonstrate highly negative Δ*G*_ad_ values (up to −125.04 kJ mol^–1^ for NO), indicating that localized defect states provide sufficient enthalpic driving force to overcome the −*T*Δ*S* barrier, leading to spontaneous chemisorption. Solvation effects play a crucial role in O_2_^–^ capture. While gas-phase values of Δ*G*_ad_ are known to be extremely exothermic, these values are attenuated in the aqueous phase (Figure 10). This is indicative of the desolvation energy required to displace the dielectric medium around the highly polarizable O_2_^–^ anion prior to adsorption. Despite this attenuation, the aqueous free energies remain favorable for the strongly bound complexes under the modeled continuum conditions.

**Figure 10:**
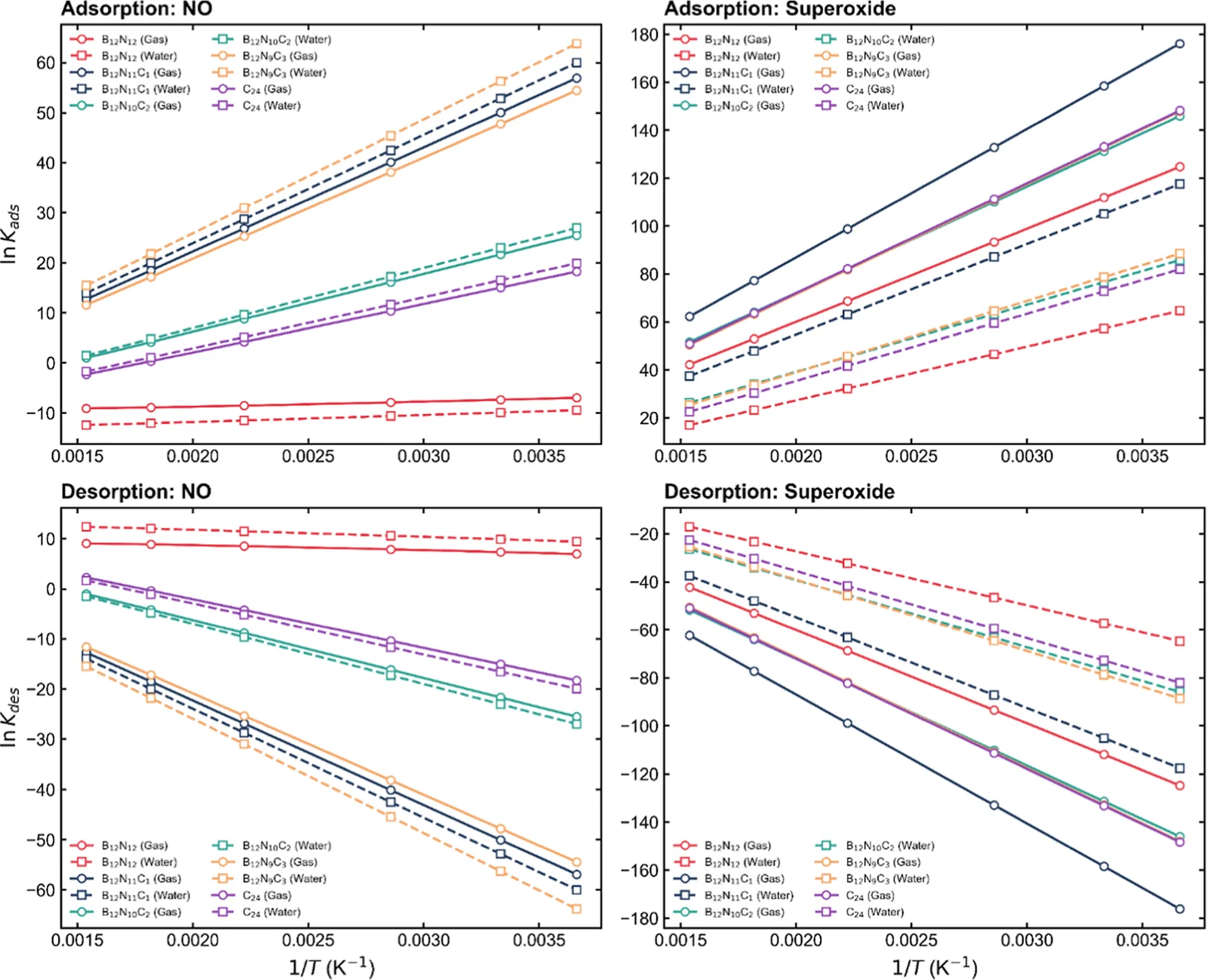
Van’t Hoff plots for the adsorption equilibria of NO and O_2_^–^ analytes on B_12_N_12_ and C-doped BNFs. **Note:** The linear correlation between ln *K* and 1/*T* across gas (solid lines) and aqueous (dashed lines) phases illustrates the temperature dependence of the equilibrium constants, providing the basis for calculating the corresponding enthalpic (Δ*H*_ad_) and entropic changes (Δ*S*_ad_).

The calculated thermodynamic functions describe adsorption favorability within 298.15–700 K under the harmonic, continuum-solvation, and adopted standard-state approximations. Equilibrium temperatures above 700 K are model-dependent extrapolations and should not be interpreted as practical operating limits. While the results support persistent adsorption for strongly bound complexes under modeled near-ambient conditions, they do not establish reversibility, regeneration, kinetic accessibility, or physiological performance.

### 3.5 Optical Analysis

After characterizing the thermodynamic favorability and modeling the kinetic retention of the adsorbed radicals, TD-DFT was used to evaluate the optical properties of the minimum–energy complexes in gas and aqueous phases. These evaluations were performed to determine whether adsorption produces potentially detectable spectral changes. The calculated excitations span 180–800 nm, with predominant deep-ultraviolet (UV) transitions and spectral redistribution dependent on the system, induced by C-doping, radical adsorption, and solvation. The long-range-corrected ωB97X-D functional and the diffuse and polarized 6-311++G­(d,p) basis set support the description of localized and CT excitations, including states involving anionic or partially charge-separated complexes.

Theoretical UV–vis absorption spectra (Figure 11) show notable changes in the optical response of the pure and C-doped BNF systems when they interact with NO and O_2_^–^. The TD-DFT spectra reveal spectral redistribution dependent on the adsorbate, composition, and solvent rather than a universal bathochromic or hyperchromic response. Some complexes develop new near-UV or visible features, while others exhibit blue shifts or negligible changes in the 300–600 nm region.

**Figure 11:**
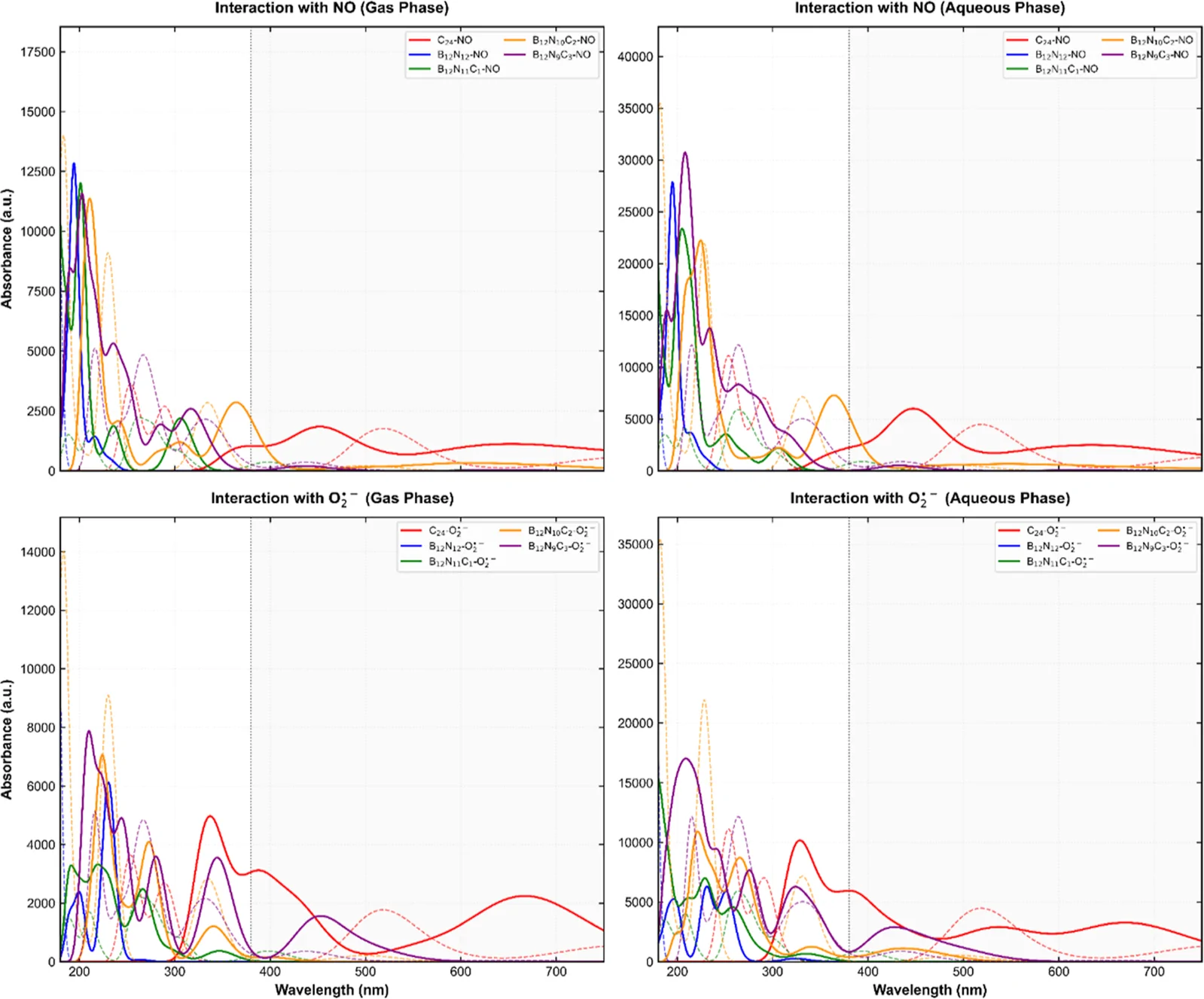
Theoretical UV–Vis absorption spectra obtained from TD-DFT calculations for pristine and C-doped BNF-based complexes and their NO and O_2_^–^ adsorbates, evaluated in the gas phase and in aqueous environment, illustrating solvent-induced effects, electronic transitions, and spectral shifts across the ultraviolet and visible regions. The spectral range begins at 180 nm, corresponding to the water cutoff, to avoid solvent polarization artifacts. All calculations were performed at the ωB97X-D/6-311++G­(d,p) level of theory.

C-doped systems exhibit mixed or CT transitions, indicating electronic redistribution induced by the presence of C and interaction with NO and O_2_^–^. C-doping promotes CT character by introducing defect-localized states that facilitate electronic polarization. These transitions involve occupied π-type orbitals, distributed over the B–N and B–C bonds, to π* orbitals extended over the BNF. B_12_N_12_ maintains π → π* transitions, which are typical of this structure, even after adsorption of the molecules. Upon adsorption of NO or O_2_^–^, additional absorption features emerge in the near-UV and visible regions. This is consistent with the formation of donor–acceptor complexes, where CT excitations contribute to the low-energy optical response.

The results of the TD-DFT indicate that the optical response of the BNFs is modified by radical adsorption, which occurs through adsorbate–substrate electronic coupling and solvent-induced polarization. The comparison of PCM/SMD shows that these optical changes are not described by a uniform red-shift or intensity-enhancement pattern. The magnitude and direction of the spectral shift depend on the adsorbate and the C-doping configuration. Pristine B_12_N_12_–NO and B_12_N_11_C_1_–NO exhibit no significant transitions in the 300–600 nm range under either solvent model. By contrast, C-doped systems, such as B_12_N_10_C_2_–NO and B_12_N_9_C_3_–NO, exhibit solvent-sensitive bands within this range. The strongest transition for B_12_N_10_C_2_–NO is relatively robust, shifting from 457.57 nm in PCM to 463.70 nm in SMD with a similar oscillator strength. B_12_N_9_C_3_–NO shows much greater dependence on the solvent model, shifting from 302.12 nm in PCM to 419.26 nm in SMD and exhibiting a decrease in oscillator strength. O_2_^–^ adsorption generates optical features in C-doped systems as well, but its response is highly system-dependent. B_12_N_10_C_2_–O_2_^–^ shows a red shift from 402.91 to 428.66 nm, while B_12_N_11_C_1_–O_2_^–^ and B_12_N_9_C_3_–O_2_^–^ exhibit moderate blue shifts. B_12_N_9_C_3_–O_2_^–^ has the highest oscillator strength of the analyzed O_2_^–^ complexes, increasing from 0.0612 in PCM to 0.0700 in SMD. This suggests strong adsorbate–substrate electronic coupling. The predicted UV–vis bands are solvent-dependent optical fingerprints of radical adsorption rather than fixed, universal sensing peaks. This behavior is consistent with NBO and electronic descriptor analyses. While HL_gap_ and charge transfer trends remain qualitatively stable, TD-DFT peak positions and oscillator strengths are more sensitive to continuum solvent models, especially in the 300–600 nm region.

The calculated optical response indicates that radical adsorption on C-doped BNFs could be probed by UV–vis measurements. The predicted transitions should therefore be regarded as proposed optical fingerprints rather than as standalone sensing signals. To ensure the accuracy of experimental results, it is essential to compare the pristine BNF, isolated radicals, adsorbed complexes, solvent background, and possible interfering ROS/RNS species. Aggregation, explicit solvation, and surface heterogeneity may also broaden or shift the bands. Therefore, UV–vis detection is plausible, but practical sensing applicability requires experimental validation.

The CT and mixed-character transitions predicted by TD-DFT provide complementary evidence of adsorption-induced electronic redistribution in the C-doped BNF complexes. These excited-state features should not be interpreted as direct measures of ground-state binding strength or kinetic stability. A comparison with desorption profiles reveals a qualitative, system-dependent correspondence: complexes exhibiting pronounced orbital mixing and low-energy optical transitions also display large effective electronic escape thresholds and long PES-MFPT residence-time projections. This trend is consistent with strong adsorbate–BNF interactions, but it does not establish a universal quantitative correlation between spectral shifts and desorption kinetics. Solvation modifies both the optical response and the desorption profiles, although these effects arise from distinct excited- and ground-state contributions. The predicted spectral changes are therefore best regarded as complementary optical fingerprints of adsorption rather than independent predictors of thermal retention.

### 3.6 Current Limitations and Future Validation

The present results provide a detailed quantum-chemical description of the adsorption of NO and O_2_^–^ on pristine and C-doped BNFs. The proposed ROS/RNS scavenging behavior should be interpreted as a theoretical prediction rather than direct evidence of biomedical applicability. The calculations were performed on isolated BNF molecular models that considered a finite number of C-doping substitution patterns in idealized gas-phase or aqueous environments. The effects of full explicit solvation, ionic strength, pH and other biomolecular parameters were not evaluated. The strong adsorption energies predicted for some radical species suggest efficient radical trapping but raise questions about spontaneous regeneration and long-term reusability. Due to these factors, the present systems should be considered theoretical candidates or design leads for ROS/RNS capture rather than validated biomedical antioxidants.

Future validation should combine the experimental synthesis and characterization of C-doped BNFs with radical-quenching and biocompatibility assays. XPS, Raman, FTIR, EPR, and UV–vis spectroscopy could confirm C incorporation, defect formation, radical binding, and adsorption-induced optical changes. EPR spin-trapping experiments and colorimetric or fluorescent ROS/RNS assays would be useful for evaluating NO, O_2_^–^, and ONOO^–^ scavenging activity under controlled conditions. Electrochemical or thermal desorption studies would also be necessary to determine if the strongly bound radical complexes can be regenerated. Cell-compatible assays, cytotoxicity tests, oxidative-stress models, and protein/biomolecule interaction studies would also be necessary before proposing biomedical applications.

## 4 Conclusions

First-principles calculations demonstrate that substitutional C-doping provides a composition-dependent route for tuning the electronic structure and radical-binding behavior of BNFs. The incorporation of carbon has been shown to narrow the HL_gap_, introduce localized defect states, and modify the local charge and spin distributions. Pristine B_12_N_12_ exhibits a weak interaction with NO, while the C-doped cages form shorter C–N contacts and generate substantially stronger adsorption complexes. The O_2_^–^ ion exhibits a high degree of binding affinity, even to the pristine B_12_N_12_ surface. The C-doping process modulates the adsorption strength and bonding motif of the O_2_^–^ ion, rather than inducing new chemisorption processes. The combined adsorption energies, bond distances, charge-transfer results, spin-density distributions, and QTAIM, ELF, and LOL descriptors support defect-mediated electronic coupling and the formation of localized C–N, C–O, and B–O interactions. The redistribution of spin density from the adsorbate to the C-doped BNF suggests that the observed polarization does not originate exclusively from the unpaired electron of the isolated radical.

The calculated thermodynamic parameters predict favorable adsorption under the modeled standard-state conditions. The highly exothermic adsorption of O_2_^–^ should be interpreted as efficient and potentially persistent radical trapping, which may favor capture applications but could restrict reversibility and material regeneration. The high-temperature Langevin simulations were utilized as accelerated stability tests and do not represent the proposed operating conditions. The PES-derived effective escape thresholds, direct Langevin trajectories, and MFPT analysis serve to distinguish weak, short-lived adsorption from more persistent chemisorption. Given that several dissociation profiles were found to be monotonic or plateau-like, it was determined that a conventional TS could not be applied to every system in this study. As a result, the long-time MFPT values are interpreted as model-dependent retention regimes rather than as exact macroscopic lifetimes.

The isolated C-doped BNFs remained structurally intact during 300 K ADMP, indicating that the rearrangements observed in selected complexes are adsorbate-induced rather than intrinsic cage instabilities. The radical-bound trajectories distinguish molecular desorption, molecular retention, defect reconstruction, O–O dissociation, and reactive trapping as composition- and medium-dependent finite-temperature regimes. A precursor-side B_12_N_12_ trajectory additionally exhibits spontaneous transient ONOO^–^-like coupling through an N26–O28 interaction distinct from the N26–O27 connectivity followed by the static IRC. TD-DFT calculations reveal adsorbate- and composition-dependent spectral shifts, in addition to CT and mixed excitations, suggesting the potential for observable optical responses. These optical changes provide additional evidence of electronic coupling. The comparative results preclude a single, universal ranking: B_12_N_11_C_1_ provides the strongest static O_2_^–^ adsorption but undergoes dissociative adsorbate-induced reconstruction, B_12_N_9_C_3_ shows strong dual reactivity accompanied by pronounced structural reorganization and reactive trapping, and B_12_N_10_C_2_ shows preferential retention of oxygen-derived species over NO, with molecular O_2_^–^ preserved in the gas-phase coadsorption trajectory and reactive oxygen trapping observed in PCM water.

## Supplementary Material



## Notes

Quantum descriptors; BSSE analysis; spin-contamination diagnostics; NBO charge-transfer analysis; condensed Fukui-function analysis; coadsorption and ONOO^–^ TS/IRC analysis; explicit-water microhydration models; PCM/SMD solvent-model sensitivity analysis; finite-temperature ADMP molecular-dynamics analysis; supplementary tables and figures (PDF)
