# Supplementary material for: Point Defect-Mediated Electronic Tuning and Thermal Stability of Carbon-Doped Boron-Nitride Fullerenes for Radical Scavenging
Source: ACS Omega. 2026 Oct 21;11(43):66499–524. doi: 10.1021/acsomega.6c05661 (PMC13736976; doi:10.1021/acsomega.6c05661)
Supplement: Supplementary file 1 [file ao6c05661_si_001.pdf]

Supporting Information

Point defect-mediated electronic tuning and thermal stability of carbon-doped boron-nitride fullerenes for radical scavenging

Gabriel García-Laiton[a,*], Edith Corona-Jiménez[a], Kerry J. Wrighton Araneda[b] and Ernesto Chigo-Anota[a,*]

[a]*Benemérita Universidad Autónoma de Puebla, Facultad de Ingeniería Química, Ciudad Universitaria, San Manuel, Puebla, Código Postal 72570, México.*

[b]*Escuela de Kinesiología, Facultad de Ciencias de la Salud, Centro Integrativo de Biología y Química Aplicada (CIBQA), Universidad Bernardo O'Higgins, General Gana 1702, Santiago, 8370854, Chile.*

[*] Email: gl225570103@alm.buap.mx

[*] Email: ernesto.chigo@correo.buap.mx

Section 1. Quantum descriptors

Table S1. Bond length (l; Å), HOMO-LUMO gap (HL_{gap}; eV), Dipole moment (DM; Debye), Chemical potential (μ; eV), Work function (WF; eV), Polarizability (α; a. u.), Global Hardness (η; eV), Global Softness (S; eV^{-1}), and Electrophilicity (ω; eV) for C-doped BNFs and test molecules in anionic charge. Reported at the level of ωB97X-D/6-311++G (d,p).

Structure	$B_{12}N_{12}$		$B_{12}N_{11}C_1$		$B_{12}N_{10}C_2$		$B_{12}N_9C_3$		C_{24}*		NO		O_2^-	
Property	Gas	Water	Gas	Water	Gas	Water	Gas	Water	Gas	Water	Gas	Water	Gas	Water
L	1.40, 1.60 (B-B)<br>1.86, 1.91 (B-N)		1.90, 1.96 (B-B)<br>1.49, 1.52 (B-C)<br>1.41, 1.53 (B-N)		1.89, 2.00 (B-B)<br>1.52, 1.55 (B-C)<br>1.43, 1.50 (B-N)		1.85, 1.89 (B-B)<br>1.52, 1.58 (B-C)<br>1.42, 1.51 (B-N)<br>1.88 (C-C)		1.39, 1.53 (C-C)		1.26 (N-O)		1.33 (O-O)	
$\mathbf{HL_{gap}}$	7.13	8.19	7.62	8.76	7.17	7.67	6.88	7.68	3.00	3.74	10.52	11.16	8.69	9.16
**DM**	4.93	7.52	4.12	6.53	3.39	5.15	4.25	5.37	0.18	2.64	0.92	0.85	0.00	0.00
**μ**	-0.16	-3.12	-0.38	-3.34	-0.85	-4.01	-0.59	-4.15	-2.06	-4.76	3.09	-2.59	4.22	-1.74
**WF**	3.56	4.09	3.81	4.38	3.59	3.83	3.44	3.84	1.50	1.87	5.26	5.58	4.35	4.58
α	187.85	263.50	178.25	257.39	190.65	286.79	194.40	301.37	243.05	417.05	19.16	20.78	14.62	16.67
η	3.56	4.09	3.81	4.38	3.59	3.83	3.44	3.84	1.50	1.87	5.26	5.58	4.35	4.58
**S**	0.14	0.12	0.13	0.11	0.14	0.13	0.15	0.13	0.33	0.27	0.10	0.09	0.12	0.11
ω	0.00	1.19	0.02	1.27	0.10	2.09	0.05	2.24	1.41	6.04	0.91	0.60	2.05	0.33

* Calculations were performed using the Restricted Open-shell DFT (RO-DFT) formalism to ensure the absence of spin contamination.

Table S2. Bond length (l; Å), HOMO-LUMO gap (HL_{gap}; eV), Dipole moment (DM; Debye), Chemical potential (μ; eV), Work function (WF; eV), Polarizability (α; a. u.), Global Hardness (η; eV), Global Softness (S; eV^{-1}), and Electrophilicity (ω; eV) for C-doped BNFs and test molecules in cationic charge. Reported at the level of ωB97X-D/6-311++G (d,p).

Structure	$B_{12}N_{12}$		$B_{12}N_{11}C_1$		$B_{12}N_{10}C_2$		$B_{12}N_9C_3$		C_{24}*		NO		O_2	
Property	Gas	Water	Gas	Water	Gas	Water	Gas	Water	Gas	Water	Gas	Water	Gas	Water
L	1.88, 2.06 (B-B)<br>1.41, 1.50 (B-N)		1.86, 1.92 (B-B)<br>1.57, 1.59 (B-C)<br>1.41, 1.51 (B-N)		1.86, 1.92 (B-B)<br>1.53, 1.62 (B-C)<br>1.40, 1.50 (B-N)<br>1.64 (C-C)		1.86, 1.99 (B-B)<br>1.51, 1.63 (B-C)<br>1.39, 1.50 (B-N)<br>1.65 (C-C)		1.39, 1.53 (C-C)		1.06 (N-O)		1.10 (O-O)	
$\mathbf{HL_{gap}}$	7.76	7.95	6.10	6.32	7.79	7.88	7.77	7.88	5.30	5.30	16.42	16.49	11.62	11.69
**DM**	3.04	4.84	3.93	5.99	2.97	4.41	3.04	4.45	0.48	0.77	0.42	0.41	0.00	0.00
**μ**	-10.28	-6.80	-10.98	-7.51	-9.96	-6.49	-9.90	-6.47	-9.59	-6.08	-19.18	-12.69	-19.69	-13.06
**WF**	3.88	3.97	3.05	3.16	3.90	3.94	3.89	3.94	2.65	2.65	8.21	8.25	5.81	5.84
α	165.11	237.07	159.53	227.37	159.72	229.11	166.81	242.10	199.44	331.99	6.16	6.77	5.46	5.99
η	3.88	3.97	3.05	3.16	3.90	3.94	3.89	3.94	2.65	2.65	8.21	8.25	5.81	5.84
**S**	0.13	0.13	0.16	0.16	0.13	0.13	0.13	0.13	0.19	0.19	0.06	0.06	0.09	0.09
ω	13.61	5.83	19.74	8.91	12.73	5.34	12.61	5.31	17.37	6.98	22.41	9.76	33.36	14.60

* Calculations were performed using the Restricted Open-shell DFT (RO-DFT) formalism to ensure the absence of spin contamination.

Table S3. Energy adsorption (E_{ad}; eV), Bond length (l; Å), HOMO-LUMO gap (HL_{gap}; eV), Dipole moment (DM; Debye), Chemical potential (μ; eV), Work function (WF; eV), Polarizability (α; a. u.), Global Hardness (η; eV), Global Softness (S; eV^{-1}), and Electrophilicity (ω; eV) in **gas phase** for BNFs + NO in anionic and cationic states. Reported at the level of ωB97X-D/6-311++G (d,p).

Structure	$B_{12}N_{12}$-NO		$B_{12}N_{11}C_1$-NO		$B_{12}N_{10}C_2$-NO		$B_{12}N_9C_3$-NO		C_{24}-NO	
Quantum descriptor	Anion	Cation	Anion	Cation	Anion	Cation	Anion	Cation	Anion	Cation
$\mathbf{E_{ad}}$	-2.86	-1.51	0.17	-2.89	-2.14	-1.43	-0.13	-0.38	-1.38	-0.83
L	1.54 (B-N)	1.94 (N-N)	1.54 (B-N)	1.34 (C-N)	1.40 (C-N)	1.33 (C-N)	3.12 (C-N)<br>3.46 (O-N)	1.78 (B-N)	1.48 (C-N)	1.52 (C-N)
$\mathbf{HL_{gap}}$	8.16	6.54	7.26	7.30	8.00	7.08	6.44	7.08	5.46	5.29
**DM**	5.73	7.91	5.73	4.09	5.81	3.05	3.09	3.46	1.14	2.61
**μ**	-0.78	-10.30	-1.17	-10.22	-0.73	-10.24	-0.87	-9.83	-0.91	-9.67
**WF**	4.08	3.27	3.63	3.65	4.00	3.54	3.22	3.54	2.73	2.65
α	181.73	175.23	188.55	174.20	196.65	185.55	206.20	185.92	254.22	216.99
η	4.08	3.27	3.63	3.65	4.00	3.54	3.22	3.54	2.73	2.65
**S**	0.12	0.15	0.14	0.14	0.13	0.14	0.16	0.14	0.18	0.19
ω	0.08	16.22	0.19	14.31	0.07	14.81	0.12	13.66	0.15	17.67

Table S4. QTAIM, ELF, and LOL analysis of BNFs–NO+O_2^- interactions in both gas and aqueous phase, evaluated in BCPs (atomic units).

Complex	Bond	H(r)	$\nabla^2\rho$	ρ	G(r)	V(r)	G(r)/|V(r)|	ELF	LOL	Bond Type
$B_{12}N_{12}$-NO+O_2^- (Gas)	B-O	-0.1094	0.5894	0.1547	0.2568	-0.3662	0.7012	0.1990	0.3327	Covalent Bond
$B_{12}N_{12}$-NO+O_2^- (Water)	B-O	-0.1039	0.5775	0.1500	0.2483	-0.3522	0.7050	0.1935	0.3288	Covalent Bond
$B_{12}N_{11}C_1$-NO+O_2^- (Gas)	B-N	-0.1207	0.3077	0.1476	0.1976	-0.3183	0.6208	0.2684	0.3770	Covalent Bond
$B_{12}N_{11}C_1$-NO+O_2^- (Gas)	C-O	-0.3148	-0.1864	0.2399	0.2682	-0.5829	0.4600	0.4958	0.4979	Covalent Bond
$B_{12}N_{11}C_1$-NO+O_2^- (Water)	B-N	-0.1110	0.3049	0.1400	0.1873	-0.2983	0.6278	0.2555	0.3692	Covalent Bond
$B_{12}N_{11}C_1$-NO+O_2^- (Water)	C-O	-0.3155	-0.1660	0.2400	0.2740	-0.5894	0.4648	0.4854	0.4927	Covalent Bond
$B_{12}N_{10}C_2$-NO+O_2^- (Gas)	N-N	0.0013	0.0258	0.0075	0.0052	-0.0039	1.3310	0.0245	0.1370	Very Weak Interaction
$B_{12}N_{10}C_2$-NO+O_2^- (Gas)	C-O	-0.3167	-0.1135	0.2401	0.2883	-0.6051	0.4766	0.4602	0.4801	Covalent Bond
$B_{12}N_{10}C_2$-NO+O_2^- (Water)	N-N	0.0008	0.0205	0.0068	0.0044	-0.0036	1.2153	0.0279	0.1413	Very Weak Interaction
$B_{12}N_{10}C_2$-NO+O_2^- (Water)	C-O	-0.3187	-0.0736	0.2408	0.3003	-0.6190	0.4851	0.4426	0.4712	Covalent Bond
$B_{12}N_9C_3$-NO+O_2^- (Gas)	B-N	-0.1213	0.6648	0.1611	0.2875	-0.4088	0.7033	0.1870	0.3240	Covalent Bond
$B_{12}N_9C_3$-NO+O_2^- (Gas)	B-O	-0.1103	0.5121	0.1515	0.2383	-0.3485	0.6837	0.2119	0.3415	Covalent Bond
$B_{12}N_9C_3$-NO+O_2^- (Water)	B-N	-0.1159	0.6714	0.1574	0.2837	-0.3996	0.7100	0.1795	0.3185	Covalent Bond
$B_{12}N_9C_3$-NO+O_2^- (Water)	B-O	-0.0999	0.4977	0.1428	0.2243	-0.3242	0.6919	0.1995	0.3330	Covalent Bond
C_{24}-NO+O_2^- (Gas)	C-N	-0.2810	-0.7019	0.2705	0.1055	-0.3864	0.2730	0.9046	0.7549	Covalent Bond

Complex	Bond	H(r)	$\nabla^2\rho$	ρ	G(r)	V(r)	G(r)/|V(r)|	ELF	LOL	Bond Type
C_{24}-NO+O_2^- (Gas)	C-O	-0.3242	-0.5081	0.2539	0.1971	-0.5213	0.3782	0.6872	0.5972	Covalent Bond
C_{24}-NO+O_2^- (Gas)	C-O	-0.3017	-0.4903	0.2456	0.1792	-0.4809	0.3726	0.7042	0.6068	Covalent Bond
C_{24}-NO+O_2^- (Water)	C-N	-0.2823	-0.7062	0.2712	0.1057	-0.3880	0.2725	0.9049	0.7552	Covalent Bond
C_{24}-NO+O_2^- (Water)	C-O	-0.3202	-0.4926	0.2516	0.1970	-0.5172	0.3810	0.6810	0.5937	Covalent Bond
C_{24}-NO+O_2^- (Water)	C-O	-0.2966	-0.4737	0.2428	0.1781	-0.4747	0.3753	0.6987	0.6036	Covalent Bond

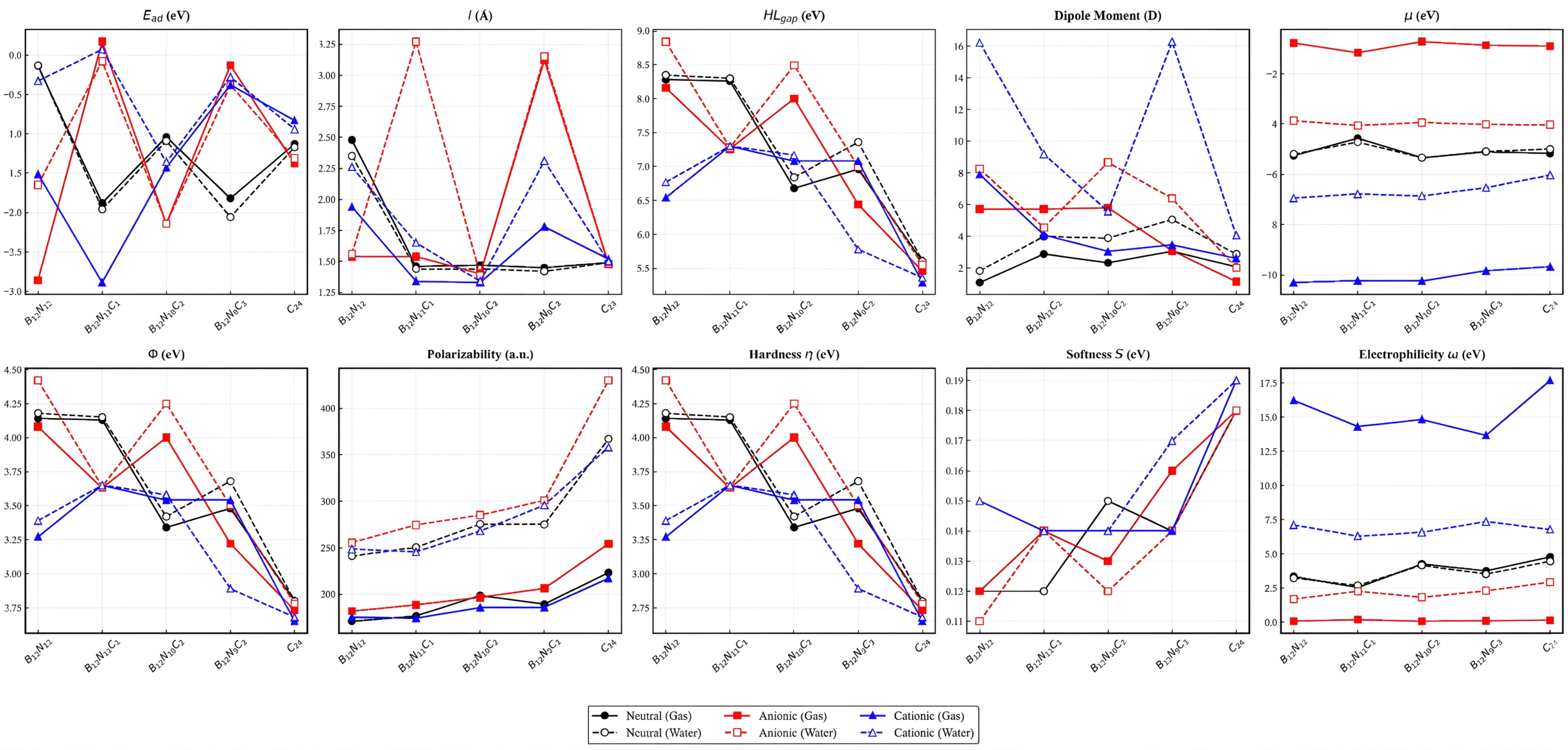

Figure S1: Comparison of quantum descriptors for NO adsorption on the studied BNFs under different charge states (anionic, neutral, and cationic).

Section 2. BSSE analysis

Gas-phase counterpoise calculations were performed using the Boys–Bernardi CP2 scheme. In this scheme, the BNF and the adsorbed radical were treated as two separate fragments at the same ωB97X-D/6-311++G(d,p) level that was used for the adsorption calculations. These calculations were restricted to the gas phase because Gaussian 16 does not permit counterpoise fragments in SCRF calculations. The CP2 analysis was used as a gas-phase sensitivity test, and the aqueous adsorption energies were discussed at the uncorrected level.

Table S5. Gas-phase Boys–Bernardi counterpoise (CP2) estimates of BSSE corrections for BNF–radical interaction energies. Corrections were evaluated at the ωB97X-D/6-311++G(d,p) level.

System	Adsorbate	E_{raw} / eV	E_{CP} / eV	BSSE correction / eV	Change / %
$B_{12}N_{12}$	NO	-0.153	-0.119	0.034	22.10
$B_{12}N_{12}$	O_2^-	-4.716	-4.623	0.093	1.98
$B_{12}N_{11}C_1$	NO	-2.173	-2.107	0.065	2.99
$B_{12}N_{11}C_1$	O_2^-	-5.937	-5.825	0.113	1.90
$B_{12}N_{10}C_2$	NO	-1.922	-1.857	0.065	3.41
$B_{12}N_{10}C_2$	O_2^-	-4.890	-4.810	0.088	1.64
$B_{12}N_9C_3$	NO	-2.157	-2.093	0.064	2.96
$B_{12}N_9C_3$	O_2^-	-6.031	-5.922	0.109	1.80
C_{24}	NO	-0.943	-0.862	0.082	8.64
C_{24}	O_2^-	-6.331	-6.212	0.119	1.88

System	Adsorbate	$E_{ad}^{reported}$ / eV	BSSE correction / eV	E_{ad}^{BSSE} / eV	Change / %
$B_{12}N_{12}$	NO	-0.14	0.034	-0.11	24.3
$B_{12}N_{12}$	O_2^-	-3.40	0.093	-3.31	2.7
$B_{12}N_{11}C_1$	NO	-1.88	0.065	-1.81	3.5
$B_{12}N_{11}C_1$	O_2^-	-4.69	0.113	-4.58	2.4
$B_{12}N_{10}C_2$	NO	-1.04	0.065	-0.97	6.3
$B_{12}N_{10}C_2$	O_2^-	-3.89	0.088	-3.80	2.2
$B_{12}N_9C_3$	NO	-1.82	0.064	-1.76	3.5
$B_{12}N_9C_3$	O_2^-	-4.00	0.109	-3.89	2.7
C_{24}	NO	-0.85	0.082	-0.77	9.6
C_{24}	O_2^-	-3.92	0.119	-3.80	3.0

CP2 results show that BSSE does not alter main adsorption trends. The largest relative BSSE effect occurs with the weakest interaction: pristine $B_{12}N_{12}$–NO. After counterpoise correction, the interaction energy changes from -0.153 to -0.119 eV. While this corresponds to a significant percentage because the initial interaction energy is small, the system remains weakly physisorbed. For the C-doped NO complexes, the correction is approximately 0.064–0.065 eV, which is about 3% of the raw interaction energy. For the O_2^- complexes, the absolute corrections are slightly larger (0.093–0.119 eV) but represent less than 2% of their very strong interaction energies. Thus, as expected, BSSE makes the adsorption energies slightly less exothermic but does not modify the physisorption/chemisorption assignment or the conclusion that C-doping enhances radical anchoring.

Section 3. Spin contamination analysis

Spin contamination was evaluated for all unrestricted open-shell calculations by comparing the computed values of $\langle S^2 \rangle$ before and after spin annihilation with the expected value of S(S+1). Most BN-based open-shell systems showed negligible residual spin contamination after annihilation. Doublet BN systems converged to values close to 0.750; triplet systems, to approximately 2.000; and quartet coadsorption systems, to approximately 3.750. Therefore, spin contamination does not significantly affect the electronic structures used to support the BN adsorption trends. The only exceptions are the C_{24}–NO doublet systems, which have a residual value of around 0.939 after annihilation, and the C_{24}–O_2^- systems, which have a residual value of around 0.790. Only the charge states used in the main single- and co-adsorption data sets are included.

Table S6. Spin contamination analysis evaluated for all unrestricted open-shell systems.

**Category**	**BNF**	**Adsorbate**	**Phase**	**Charge**	**M**	$\langle S^2 \rangle_{expected}$	$\langle S^2 \rangle_{before}$	$\langle S^2 \rangle_{after}$	$\Delta \langle S^2 \rangle_{expected}$	**Diagnostic**
Single adsorption	$B_{12}N_{12}$	NO	Gas	0	2	0.7500	0.7528	0.7500	0.0000	Negligible
Single adsorption	$B_{12}N_{12}$	NO	Water	0	2	0.7500	0.7526	0.7500	0.0000	Negligible
Single adsorption	$B_{12}N_{12}$	O_2^-	Gas	-1	2	0.7500	0.7543	0.7500	0.0000	Negligible
Single adsorption	$B_{12}N_{12}$	O_2^-	Water	-1	2	0.7500	0.7543	0.7500	0.0000	Negligible
Single adsorption	$B_{12}N_{11}C_1$	NO	Gas	0	1	0.0000	—	—	—	No S^2 diagnostic printed
Single adsorption	$B_{12}N_{11}C_1$	NO	Water	0	1	0.0000	—	—	—	No S^2 diagnostic printed
Single adsorption	$B_{12}N_{11}C_1$	O_2^-	Gas	-1	1	0.0000	—	—	—	No S^2 diagnostic printed
Single adsorption	$B_{12}N_{11}C_1$	O_2^-	Water	-1	1	0.0000	—	—	—	No S^2 diagnostic printed
Single adsorption	$B_{12}N_{10}C_2$	NO	Gas	0	2	0.7500	0.7785	0.7503	0.0003	Negligible
Single adsorption	$B_{12}N_{10}C_2$	NO	Water	0	2	0.7500	0.7650	0.7501	0.0001	Negligible
Single adsorption	$B_{12}N_{10}C_2$	O_2^-	Gas	-1	2	0.7500	0.7544	0.7500	0.0000	Negligible
Single adsorption	$B_{12}N_{10}C_2$	O_2^-	Water	-1	2	0.7500	0.7545	0.7500	0.0000	Negligible
Single adsorption	$B_{12}N_9C_3$	NO	Gas	0	1	0.0000	—	—	—	No S^2 diagnostic printed
Single adsorption	$B_{12}N_9C_3$	NO	Water	0	1	0.0000	—	—	—	No S^2 diagnostic printed
Single adsorption	$B_{12}N_9C_3$	O_2^-	Gas	-1	1	0.0000	—	—	—	No S^2 diagnostic printed
Single adsorption	$B_{12}N_9C_3$	O_2^-	Water	-1	1	0.0000	—	—	—	No S^2 diagnostic printed
Single adsorption	C_{24}	NO	Gas	0	2	0.7500	1.1958	0.9389	0.1889	Significant
Single adsorption	C_{24}	NO	Water	0	2	0.7500	1.1953	0.9387	0.1887	Significant
Single adsorption	C_{24}	O_2^-	Gas	-1	2	0.7500	0.9829	0.7910	0.0410	Minor
Single adsorption	C_{24}	O_2^-	Water	-1	2	0.7500	0.9808	0.7902	0.0402	Minor
Co-adsorption	$B_{12}N_{12}$	NO + O_2^-	Gas	-1	1	0.0000	—	—	—	No S^2 diagnostic printed
Co-adsorption	$B_{12}N_{12}$	NO + O_2^-	Water	-1	1	0.0000	—	—	—	No S^2 diagnostic printed
Co-adsorption	$B_{12}N_{11}C_1$	NO + O_2^-	Gas	-1	4	3.7500	3.7639	3.7501	0.0001	Negligible
Co-adsorption	$B_{12}N_{11}C_1$	NO + O_2^-	Water	-1	4	3.7500	3.7639	3.7501	0.0001	Negligible
Co-adsorption	$B_{12}N_{10}C_2$	NO + O_2^-	Gas	-1	3	2.0000	2.0080	2.0000	0.0000	Negligible
Co-adsorption	$B_{12}N_{10}C_2$	NO + O_2^-	Water	-1	3	2.0000	2.0080	2.0000	0.0000	Negligible
Co-adsorption	$B_{12}N_9C_3$	NO + O_2^-	Gas	-1	4	3.7500	3.9742	3.7557	0.0057	Negligible
Co-adsorption	$B_{12}N_9C_3$	NO + O_2^-	Water	-1	4	3.7500	3.8631	3.7523	0.0023	Negligible
Co-adsorption	C_{24}	NO + O_2^-	Gas	-1	1	0.0000	—	—	—	No S^2 diagnostic printed
Co-adsorption	C_{24}	NO + O_2^-	Water	-1	1	0.0000	—	—	—	No S^2 diagnostic printed

Notes:

- S^2 values close to 0.75, 2.00, or 3.75 indicate pure spin states (doublet, triplet, quartet).
- Deviations greater than 10% were treated as evidence of appreciable spin contamination and the corresponding results were interpreted cautiously.
- Files marked with * correspond to calculations where spin contamination correction was applied (SP or RO suffixes in original names).

Section 4. Charge-transfer analysis from NBO natural charges

This supplementary note provides quantitative support for the proposed charge-transfer mechanism and enhanced reactivity of C-doped BNF structures. The analysis was performed on 30 optimized complexes (adsorption of one molecule of NO and O_2^-, as well as co-adsorption of both). Natural Population Analysis (NBO) was performed on every complex and is therefore used as the primary charge descriptor. Mulliken and APT fragment charges were extracted for secondary, method-dependent verification. For each complex, the BNF was assigned to atoms 1 through 24. For single-adsorbate systems, atoms 25 and higher were assigned to either NO or O_2^-. For NO+O_2^- co-adsorption, the NO was assigned to the N atom and the closest O atom. The remaining two O atoms were assigned to the O_2^- fragment. Reported fragment charges are sums of atomic NBO charges. For neutral NO, $q(NO) < 0$ indicates BNF-to-NO electron donation, and $q(NO) > 0$ indicates NO-to-BNF donation. For adsorbed O_2^-, the estimated charge depletion of O_2^- toward the cage is $q(O_2) - (-1) = q(O_2) + 1$. Positive values indicate O_2^- to BNF charge transfer.

Pristine $B_{12}N_{12}$ retains a positive NO fragment charge (+0.102 *e* in gas and +0.136 *e* in water) for NO adsorption, indicating NO-to-BNF donation. The C-doped BNFs, however, reverse the direction of charge transfer. $q(NO)$ is negative for $B_{12}N_{11}C_1$, $B_{12}N_{10}C_2$, and $B_{12}N_9C_3$, with BNF-to-NO donation ranging from 0.112 to 0.248 *e*. The largest NO reduction occurs for $B_{12}N_9C_3$ in water: $q(NO) = -0.248\ e$. C substitution creates localized, negatively charged dopant centers that strongly polarize neighboring B/N sites. The adsorbates bind at or near these polarized, C-containing regions of the doped BNFs. This provides a quantitative basis for enhanced reactivity relative to pristine $B_{12}N_{12}$. This effect is most evident for NO, where the direction of charge transfer changes with C doping.

Table S7. NBO fragment charges for NO complexes.

System	Medium	q(cage)	q(NO)	Direction	|CT| / e	Nearest contact	avg q(Cdop)
$B_{12}N_{12}$	gas	-0.102	0.102	NO→cage	0.102	B9 (2.477 Å)	—
$B_{12}N_{12}$	water	-0.136	0.136	NO→cage	0.136	B9 (2.349 Å)	—
$B_{12}N_{11}C_1$	gas	0.144	-0.144	cage→NO	0.144	C1 (1.458 Å)	-0.915
$B_{12}N_{11}C_1$	water	0.189	-0.189	cage→NO	0.189	C1 (1.441 Å)	-0.900
$B_{12}N_{10}C_2$	gas	0.112	-0.112	cage→NO	0.112	C10 (1.469 Å)	-0.841
$B_{12}N_{10}C_2$	water	0.191	-0.191	cage→NO	0.191	C10 (1.444 Å)	-0.823
$B_{12}N_9C_3$	gas	0.158	-0.158	cage→NO	0.158	C18 (1.455 Å)	-0.822
$B_{12}N_9C_3$	water	0.248	-0.248	cage→NO	0.248	C18 (1.416 Å)	-0.812
C_{24}	gas	0.092	-0.092	cage→NO	0.092	C16 (1.494 Å)	0.004
C_{24}	water	0.112	-0.112	cage→NO	0.112	C16 (1.488 Å)	0.005

Table S8. NBO fragment charges for O_2^- complexes.

System	Medium	q(cage)	q(O_2)	Direction	O_2^- depletion / e	Nearest contact	avg q(Cdop)
$B_{12}N_{12}$	gas	-0.229	-0.771	O_2^-→cage	0.229	B5 (1.492 Å)	—
$B_{12}N_{12}$	water	-0.217	-0.783	O_2^-→cage	0.217	B5 (1.510 Å)	—
$B_{12}N_{11}C_1$	gas	-0.207	-0.793	O_2^-→cage	0.207	C1 (1.425 Å)	-0.684
$B_{12}N_{11}C_1$	water	-0.164	-0.836	O_2^-→cage	0.164	C1 (1.429 Å)	-0.692
$B_{12}N_{10}C_2$	gas	-0.234	-0.766	O_2^-→cage	0.234	B9 (1.474 Å)	-0.427
$B_{12}N_{10}C_2$	water	-0.224	-0.776	O_2^-→cage	0.224	B9 (1.486 Å)	-0.436
$B_{12}N_9C_3$	gas	-0.215	-0.785	O_2^-→cage	0.215	C20 (1.422 Å)	-0.759
$B_{12}N_9C_3$	water	-0.171	-0.829	O_2^-→cage	0.171	C20 (1.426 Å)	-0.767
C_{24}	gas	-0.692	-0.308	O_2^-→cage	0.692	C23 (1.439 Å)	-0.029
C_{24}	water	-0.685	-0.315	O_2^-→cage	0.685	C23 (1.441 Å)	-0.029

Table S9. NBO fragment charges for anionic NO+O_2^- coadsorbed complexes.

System	Medium	q(cage)	q(NO)	q(O_2)	q(ads,total)	NO contact	O_2 contact	avg q(Cdop)
$B_{12}N_{12}$	gas	-0.463	0.048	-0.585	-0.537	B1 (3.251 Å)	B1 (1.471 Å)	—
$B_{12}N_{12}$	water	-0.447	0.061	-0.614	-0.553	B1 (3.261 Å)	B1 (1.480 Å)	—
$B_{12}N_{11}C_1$	gas	-0.238	-0.414	-0.349	-0.762	B4 (1.537 Å)	C1 (1.428 Å)	-0.676
$B_{12}N_{11}C_1$	water	-0.202	-0.441	-0.357	-0.798	B4 (1.555 Å)	C1 (1.426 Å)	-0.676
$B_{12}N_{10}C_2$	gas	-0.632	0.004	-0.372	-0.368	N12 (3.060 Å)	C20 (1.423 Å)	-0.912
$B_{12}N_{10}C_2$	water	-0.611	0.008	-0.397	-0.389	N12 (3.202 Å)	C20 (1.419 Å)	-0.925
$B_{12}N_9C_3$	gas	-0.381	-0.072	-0.547	-0.619	B19 (1.457 Å)	B2 (1.492 Å)	-0.694
$B_{12}N_9C_3$	water	-0.468	0.039	-0.571	-0.532	B19 (1.464 Å)	B2 (1.510 Å)	-0.702
C_{24}	gas	-0.242	-0.150	-0.608	-0.758	C19 (1.478 Å)	C1 (1.437 Å)	-0.010
C_{24}	water	-0.226	-0.148	-0.626	-0.774	C19 (1.477 Å)	C1 (1.440 Å)	-0.009

Section 5. Condensed Fukui function analysis and connection with NBO charge transfer

To complement the NBO charge-transfer analysis and clarify the local origin of the radical-scavenging behavior, a condensed Fukui function analysis was performed on all optimized, single-molecule adsorption systems. The adsorbate was removed from the final optimized geometry of each complex, and single-point calculations were carried out on the extracted BNF using the N, N+1, and N-1 electron states without further structural relaxation. Hirshfeld atomic charges were used in all cases to avoid mixing population schemes.

The condensed descriptors were calculated using the following definitions: $f_k^+ = q_k(N) - q_k(N+1)$; $f_k^- = q_k(N-1) - q_k(N)$; $f_k^0 = \frac{q_k(N-1)-q_k(N+1)}{2}$; and $\Delta f_k = f_k^+ - f_k^-$. In this framework, f_k^+ identifies sites prone to nucleophilic attack, f_k^- identifies sites prone to electrophilic attack, f_k^0 highlights sites most susceptible to radical attack, and the dual descriptor distinguishes between locally electrophilic ($\Delta f > 0$) and nucleophilic ($\Delta f < 0$) regions.

The Fukui descriptors demonstrate that C incorporation not only modifies the global HL_{gap}, but also creates localized, chemically active centers that explain the NBO charge-transfer trends. Pristine $B_{12}N_{12}$ exhibits a positive NBO charge on NO of +0.102 e in gas and +0.136 e in water for NO adsorption, indicating charge transfer from NO to BNF. In contrast, the C-doped BNFs show negative q values, indicating BNF-to-NO charge transfer. The Fukui analysis explains this inversion. **The doped BNFs' largest radical-response sites are located at C defects or centers directly polarized by those defects**. The NBO charge-transfer reversal upon C doping is therefore not a purely global effect but rather originates from localized defect reactivity.

For $B_{12}N_{11}C_1$, the C1 dopant is the dominant radical-active site for both NO and O_2^- in both gas and water. The $f_0(C1)$ values increase from 0.249 to 0.332 for NO and from 0.232 to 0.316 for O_2^- when transitioning from gas to water. These results are consistent with the NBO analysis, which shows that **NO receives electron density from the BNF while O_2^- donates part of its excess charge to the BNF**. Therefore, $B_{12}N_{11}C_1$ behaves as a highly localized, defect-mediated scavenger; the same C center concentrates the radical response and governs interfacial polarization.

For $B_{12}N_{10}C_2$, the Fukui pattern is intermediate. In the NO complexes, the radical response is weaker and C-centered, with C10 as the leading f_0 site in both phases. For O_2^-, however, the largest f_0 values are located at B9, increasing from 0.229 in the gas phase to 0.299 in the liquid phase. This indicates that nearby C substitutions polarize a B atom into an electrophilic O_2^--binding center. The NBO data support this interpretation because the O_2^- ion transfers charge to the BNF in both media. Therefore, **$B_{12}N_{10}C_2$'s reactivity toward O_2^- is better described as C-defect-assisted B-site activation, whereas its NO response is less strongly localized.**

The Fukui analysis of $B_{12}N_9C_3$ reveals a distributed, C-centered reactivity pattern. C18 is the leading radical-active site for NO, with f_0 values of 0.229 in gas and 0.310 in water. In contrast, C20 dominates the response toward O_2^- with f_0 values of 0.191 and 0.258, respectively. **This separation of active centers supports the proposed dual-scavenging role of $B_{12}N_9C_3$**. The tri-doped BNF provides multiple chemically accessible, C-centered regions that allow NO and O_2^- to interact through distinct, yet complementary, local reactivity patterns. This complements the NBO results, which show BNF-to-NO charge transfer for NO and O_2^--to-cage charge transfer.

Pristine $B_{12}N_{12}$ exhibits the polarity expected for a BNF. The largest f^+ and f_0 values are primarily on B atoms, while the strongest f^- response is on N atoms. For NO, B9 dominates the radical response, with f_0 values of 0.192 in gas and 0.262 in water. For O_2^-, B5 dominates, with f_0 values of 0.224 and 0.282. This intrinsic B/N polarity does not produce the C-induced defect states required for robust covalent radical stabilization. This is why **pristine $B_{12}N_{12}$ can have local electrophilic B sites but lacks the charge-transfer pattern and C-centered anchoring seen in doped systems**.

The C_{24} reference cage exhibits a lower, more delocalized Fukui response. The largest f_0 values are distributed among different C atoms and are smaller than the values found for the active sites of the doped BNFs: 0.065/0.099 for NO and 0.053/0.086 for O_2^- in gas/water. While NBO suggests interfacial charge redistribution in C_{24}, Fukui descriptors indicate that this response is diffuse rather than defect-localized. Therefore, **C-doped BNFs provide more chemically defined adsorption centers than C_{24}, which is important for controlling radical capture and selectivity**.

Table S10. Integrated NBO-Fukui mechanistic summary for one-molecule adsorption systems. The table illustrates the quantum-chemical parameters obtained from NBO fragment charges, including the Hirshfeld charge-based condensed Fukui descriptor for radical attack on the extracted cage geometry, represented by f_0.

Cage	Ads.	Phase	NBO adsorbate charge / CT	Fukui radical site, f_0	Dual-descriptor extrema	Mechanistic reading
$B_{12}N_{12}$	NO	Gas	q(NO)=0.102 e; NO→cage, |CT|=0.102 e	B9=0.192	Δf+ B9=0.315; Δf- N18=-0.066	Intrinsic BN polarity gives B-centered response but no C-defect anchoring.
$B_{12}N_{12}$	NO	Water	q(NO)=0.136 e; NO→cage, |CT|=0.136 e	B9=0.262	Δf+ B9=0.439; Δf- N21=-0.079	Intrinsic BN polarity gives B-centered response but no C-defect anchoring.
$B_{12}N_{12}$	O_2^-	Gas	q(O_2^-)=-0.771 e; O2-→cage, |CT|=0.229 e	B5=0.224	Δf+ B5=0.350; Δf- N13=-0.106	Intrinsic BN polarity gives B-centered response but no C-defect anchoring.
$B_{12}N_{12}$	O_2^-	Water	q(O_2^-)=-0.783 e; O2-→cage, |CT|=0.217 e	B5=0.282	Δf+ B5=0.441; Δf- N15=-0.087	Intrinsic BN polarity gives B-centered response but no C-defect anchoring.
$B_{12}N_{11}C_1$	NO	Gas	q(NO)=-0.144 e; cage→NO, |CT|=0.144 e	C1=0.249	Δf+ B6=0.049; Δf- N14=-0.023	Single C-defect concentrates the radical response and matches NBO interfacial polarization.
$B_{12}N_{11}C_1$	NO	Water	q(NO)=-0.189 e; cage→NO, |CT|=0.189 e	C1=0.332	Δf+ B6=0.044; Δf- N14=-0.026	Single C-defect concentrates the radical response and matches NBO interfacial polarization.
$B_{12}N_{11}C_1$	O_2^-	Gas	q(O_2^-)=-0.793 e; O2-→cage, |CT|=0.207 e	C1=0.232	Δf+ B6=0.055; Δf- N3=-0.021	Single C-defect concentrates the radical response and matches NBO interfacial polarization.
$B_{12}N_{11}C_1$	O_2^-	Water	q(O_2^-)=-0.836 e; O2-→cage, |CT|=0.164 e	C1=0.316	Δf+ B6=0.050; Δf- N14=-0.023	Single C-defect concentrates the radical response and matches NBO interfacial polarization.
$B_{12}N_{10}C_2$	NO	Gas	q(NO)=-0.112 e; cage→NO, |CT|=0.112 e	C10=0.136	Δf+ C20=0.071; Δf- N18=-0.067	NO response is moderate and split over C centers, consistent with weaker/specific C-N anchoring.

Cage	Ads.	Phase	NBO adsorbate charge / CT	Fukui radical site, f_0	Dual-descriptor extrema	Mechanistic reading
$B_{12}N_{10}C_2$	NO	Water	q(NO)=-0.191 e; cage→NO, |CT|=0.191 e	C10=0.169	Δf+ C20=0.081; Δf- N18=-0.063	NO response is moderate and split over C centers, consistent with weaker/specific C-N anchoring.
$B_{12}N_{10}C_2$	O_2^-	Gas	q(O_2^-)=-0.766 e; O2-→cage, |CT|=0.234 e	B9=0.229	Δf+ B9=0.270; Δf- C20=-0.073	Nearby C defects activate B9 as an electrophilic O_2^--capture center.
$B_{12}N_{10}C_2$	O_2^-	Water	q(O_2^-)=-0.776 e; O2-→cage, |CT|=0.224 e	B9=0.299	Δf+ B9=0.361; Δf- C20=-0.085	Nearby C defects activate B9 as an electrophilic O_2^--capture center.
$B_{12}N_9C_3$	NO	Gas	q(NO)=-0.158 e; cage→NO, |CT|=0.158 e	C18=0.229	Δf+ B19=0.043; Δf- C20=-0.029	Tri-doped cage localizes NO radical response mainly at C18.
$B_{12}N_9C_3$	NO	Water	q(NO)=-0.248 e; cage→NO, |CT|=0.248 e	C18=0.310	Δf+ B19=0.037; Δf- C20=-0.026	Tri-doped cage localizes NO radical response mainly at C18.
$B_{12}N_9C_3$	O_2^-	Gas	q(O_2^-)=-0.785 e; O2-→cage, |CT|=0.215 e	C20=0.191	Δf+ B9=0.051; Δf- C10=-0.078	A different C-centered site, C20, dominates superoxide response, supporting dual-site scavenging.
$B_{12}N_9C_3$	O_2^-	Water	q(O_2^-)=-0.829 e; O2-→cage, |CT|=0.171 e	C20=0.258	Δf+ C20=0.063; Δf- C10=-0.082	A different C-centered site, C20, dominates superoxide response, supporting dual-site scavenging.
C_{24}	NO	Gas	q(NO)=-0.092 e; cage→NO, |CT|=0.092 e	C16=0.065	Δf+ C15=0.076; Δf- C16=-0.069	Lower and more distributed f_0 values indicate delocalized carbon-cage reactivity.
C_{24}	NO	Water	q(NO)=-0.112 e; cage→NO, |CT|=0.112 e	C15=0.099	Δf+ C20=0.056; Δf- C8=-0.050	Lower and more distributed f_0 values indicate delocalized carbon-cage reactivity.
C_{24}	O_2^-	Gas	q(O_2^-)=-0.308 e; O2-→cage, |CT|=0.692 e	C9=0.053	Δf+ C6=0.075; Δf- C9=-0.049	Lower and more distributed f_0 values indicate delocalized carbon-cage reactivity.
C_{24}	O_2^-	Water	q(O_2^-)=-0.315 e; O2-→cage, |CT|=0.685 e	C6=0.086	Δf+ C16=0.112; Δf- C15=-0.061	Lower and more distributed f_0 values indicate delocalized carbon-cage reactivity.

Section 6. Geometric analysis of co-adsorbed systems

Table S11. Geometrical diagnosis of possible ONOO⁻ formation in NO/O_2^- co-adsorption complexes.

System	Phase	$d_{N(NO)-O(O_2)}$ shortest / Å	Final arrangement	ONOO-like formation?
$B_{12}N_{12}$	Gas	~1.37	O–N–O–O motif	Yes
$B_{12}N_{12}$	Water	~1.38	O–N–O–O motif	Yes
$B_{12}N_{11}C_1$	Gas	~5.05	separated NO and O_2^-	No
$B_{12}N_{11}C_1$	Water	~5.04	separated NO and O_2^-	No
$B_{12}N_{10}C_2$	Gas	~8.20	separated NO and O_2^-	No
$B_{12}N_{10}C_2$	Water	~8.30	separated NO and O_2^-	No
$B_{12}N_9C_3$	Gas	~7.48	separated NO and O_2^-	No
$B_{12}N_9C_3$	Water	~7.49	separated NO and O_2^-	No
C_{24}	Gas	~7.65	separated NO and O_2^-	No
C_{24}	Water	~7.65	separated NO and O_2^-	No

Only $B_{12}N_{12}$ shows a short N(NO)–O(O_2^-) contact compatible with an ONOO-like adduct. The remaining systems show long interfragment N···O distances, indicating that NO and O_2^- remain independently adsorbed rather than recombining into peroxynitrite. Frequency calculations confirmed that the $B_{12}N_{12}$-supported ONOO-like structures are true minima on the potential-energy surface. After geometry optimization, no ONOO-like product was obtained for the C-doped BNFs and C_{24}. The NO and O_2^- fragments remained spatially separated and interacted with different regions of the BNFs. Therefore, transition-state (TS) searches for ONOO- formation were not pursued as the main mechanistic pathway for these systems. Their activity is instead discussed in terms of independent radical stabilization and co-adsorptive trapping.

The key distances are:

Phase	O(NO)–N(NO) / Å	new N(NO)–O(O_2) / Å	O–O / Å	B(BNF)–O(ONOO) / Å
gas	1.173	1.375	1.407	1.471
water	1.171	1.379	1.407	1.480

Electronic evidence of the new N–O bond in the ONOO-like product was obtained by calculating the Wiberg index.

Phase	Bond	Distance / Å	WBI
gas	N(NO)–O(O_2)	1.375	1.097
water	N(NO)–O(O_2)	1.379	1.087

The explicit NBO orbital for the new bond is:

Phase	NBO bond orbital	Occupation / e
gas	BD(1) N26–O27	1.98954
water	BD(1) N26–O27	1.98964

Because the optimized $B_{12}N_{12}$ co-adsorption structure already corresponds to an $ONOO^-$-like geometry, the static TS/IRC analysis was formulated from the product side through the reverse process. A complementary precursor-side finite-temperature ADMP trajectory is analyzed in Section 9.4.

$$B_{12}N_{12} - ONOO^- \rightarrow B_{12}N_{12} - NO \cdots O_2^-$$

Finding a TS for:

$$NO + O_2^- \rightleftharpoons ONOO^-$$

over $B_{12}N_{12}$.

The TS in the gas phase $\left(\nu_{imag} = -411.55\ cm^{-1}\right)$ is confirmed with the following distances:

Parameter	Value / Å
O(NO)–N(NO)	1.115
N(NO)–O(O_2)	2.878
O–O	1.343
B(BNF)–O	1.474

The imaginary mode primarily involves the NO/O_2 fragment, particularly atoms 25–28. This corresponds to the rearrangement of radicals towards or away from the ONOO-like adduct.

Gas-phase reaction-coordinate

Table S12. Gas-phase TS/IRC diagnostics for ONOO-like formation on $B_{12}N_{12}$

Analysis	Result	d_{N-O}/ Å	Relative energy	Interpretation
Optimized ONOO-like product	True minimum	1.375	0.00 eV	ONOO-like product confirmed as a minimum
Gas-phase TS	One imaginary frequency	2.878	+2.83 eV vs. product	First-order saddle point associated with NO/O_2 rearrangement
Forward IRC endpoint	Normal termination	1.381	−2.70 eV vs. TS	Connects toward the ONOO-like product side
Reverse IRC endpoint	Normal termination	2.636	−0.523 eV vs. TS	Connects toward the elongated precursor side

$$B_{12}N_{12} \cdots NO \cdots O_2^- \rightleftharpoons TS \rightleftharpoons B_{12}N_{12} - ONOO^-$$

The forward IRC reaches an ONOO-like geometry with:

$$d_{N-O} = 1.381 \text{ A}$$

which is very close to the optimized ONOO-like product value:

$$d_{N-O} = 1.375 \text{ A}$$

The reverse IRC elongates the N···O contact to:

$$d_{N-O} = 2.636 \text{ A}$$

which is consistent with the precursor side of the reaction coordinate.

Table S13. Gas-phase energetics for the $B_{12}N_{12}$-supported ONOO-like pathway

Quantity	Definition	Value	Description
$\Delta E^{\ddagger}_{dissociation}$	$E_{TS} - E_{ONOO}$	2.83 eV	Electronic barrier from the ONOO-like product toward dissociation
$\Delta G^{\ddagger}_{gas}$	$G_{TS} - G_{ONOO}$	2.74 eV	Gibbs free-energy barrier from the product toward the TS
$\Delta E_{TS-reverse\ endpoint}$	$E_{TS} - E_{reverse\ endpoint}$	≈ 0.52eV	Qualitative estimate from the IRC precursor side

The value of 2.83 eV should not be described as the formation barrier of free radicals. This value corresponds to the dissociation barrier from the ONOO-like product to the transition state.

$$\Delta E^{\ddagger}_{dissociation} = E_{TS} - E_{ONOO} = 2.83 \text{ eV}$$

The approximate barrier from the reverse IRC endpoint to the TS is much smaller:

$$\Delta E_{TS-reverse\ endpoint} \approx 0.52 \text{ eV}$$

This value should only be used qualitatively because the reverse IRC endpoint was not optimized as an independent minimum.

Table S14. Aqueous-phase energetics for the $B_{12}N_{12}$-supported ONOO-like pathway

Quantity	Definition	Value	Comment
$\Delta E^{\ddagger}_{water}$	$E_{TS} - E_{ONOO}$	2.07eV	Electronic barrier relative to the aqueous ONOO-like product
$\Delta G^{\ddagger}_{water}$	$G_{TS} - G_{ONOO}$	1.98eV	Gibbs free-energy barrier relative to the aqueous ONOO-like product

Section 7. Explicit-water microhydration models: $5H_2O$ and $10H_2O$

Five or ten water molecules were placed around the BNF-adsorbate binding region to represent a first hydration shell and the beginning of a second-shell network. The initial water orientations were selected to permit hydrogen bonding to the adsorbate, the adsorption atom(s), neighboring heteroatoms, and other water molecules. The complete BNF-adsorbate-water assembly was subsequently optimized in PCM water. The explicit-cluster calculations, excluding PCM, were maintained as a diagnostic tool for distinguishing short-range microhydration from long-range dielectric screening. For each complex, the adsorbate was removed without relaxing the remaining BNF-water coordinates. The energy was then evaluated for this frozen BNF-water reference in the same environment using a single-point method. The reported electronic interaction was

$$E_{ads,FH}(n, PCM) = E\left[(BNF - X)\,(H_2O)_n\right]_{PCM} - E\left[BNF\,(H_2O)_n\right]_{frozen,PCM} - E[X]_{PCM}$$

Where X is NO or O_2^- and n is 5 or 10.

The frozen-hydration vertical adsorption energy preserves the hydration arrangement of the optimized complex, avoiding the mixing of adsorption energy with a separate reorganization of the isolated, hydrated BNF. No zero-point, thermal, entropic, or standard-state corrections were included in the reported values. BSSE was evaluated as a separate sensitivity analysis using gas-phase and explicit-cluster counterpoise calculations.

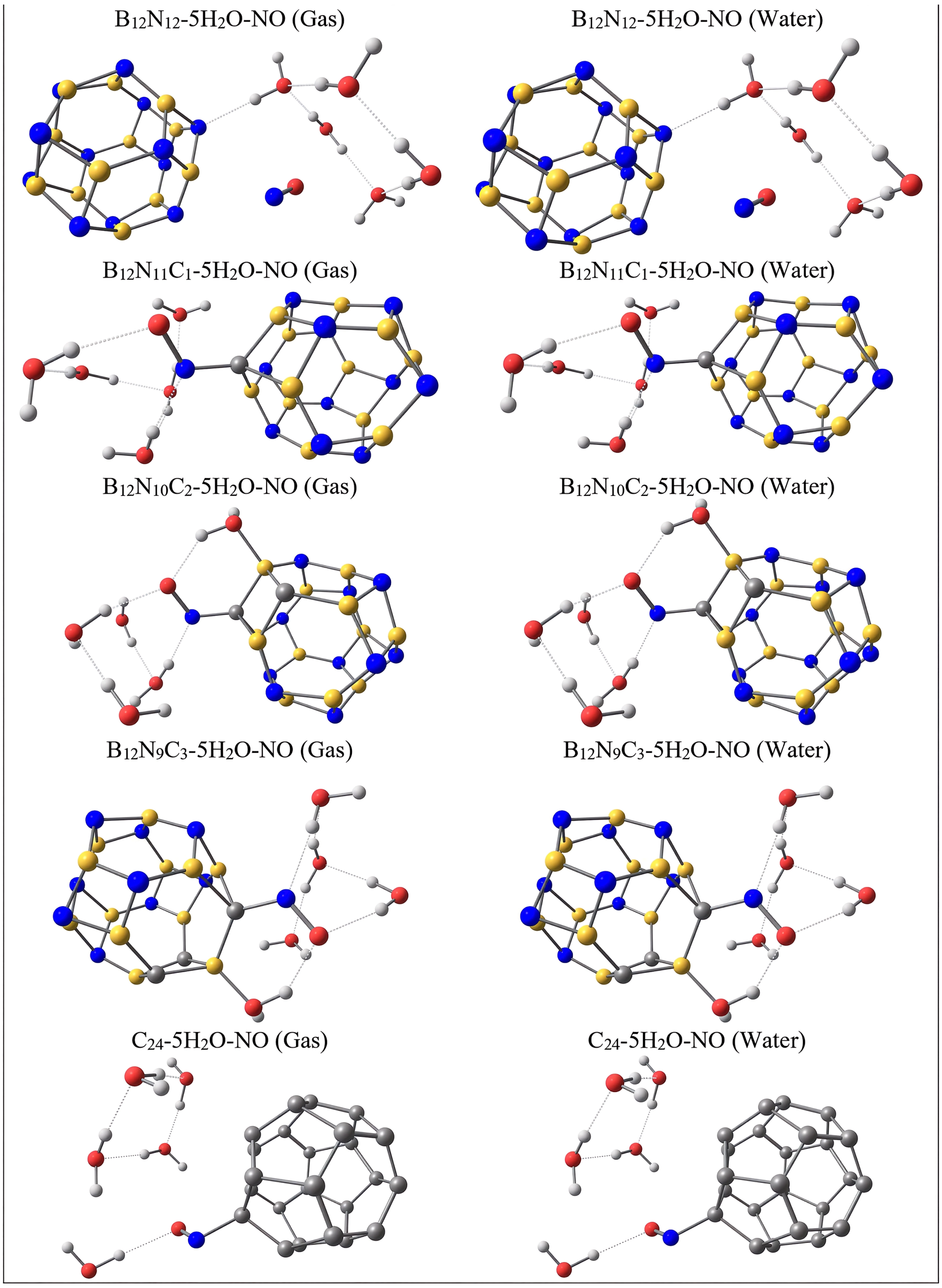

Figure S2: Microhydrated models constructed by placing five water molecules around the NO adsorption site. The $5H_2O$ cluster-continuum model was used for the primary local hydration comparison.

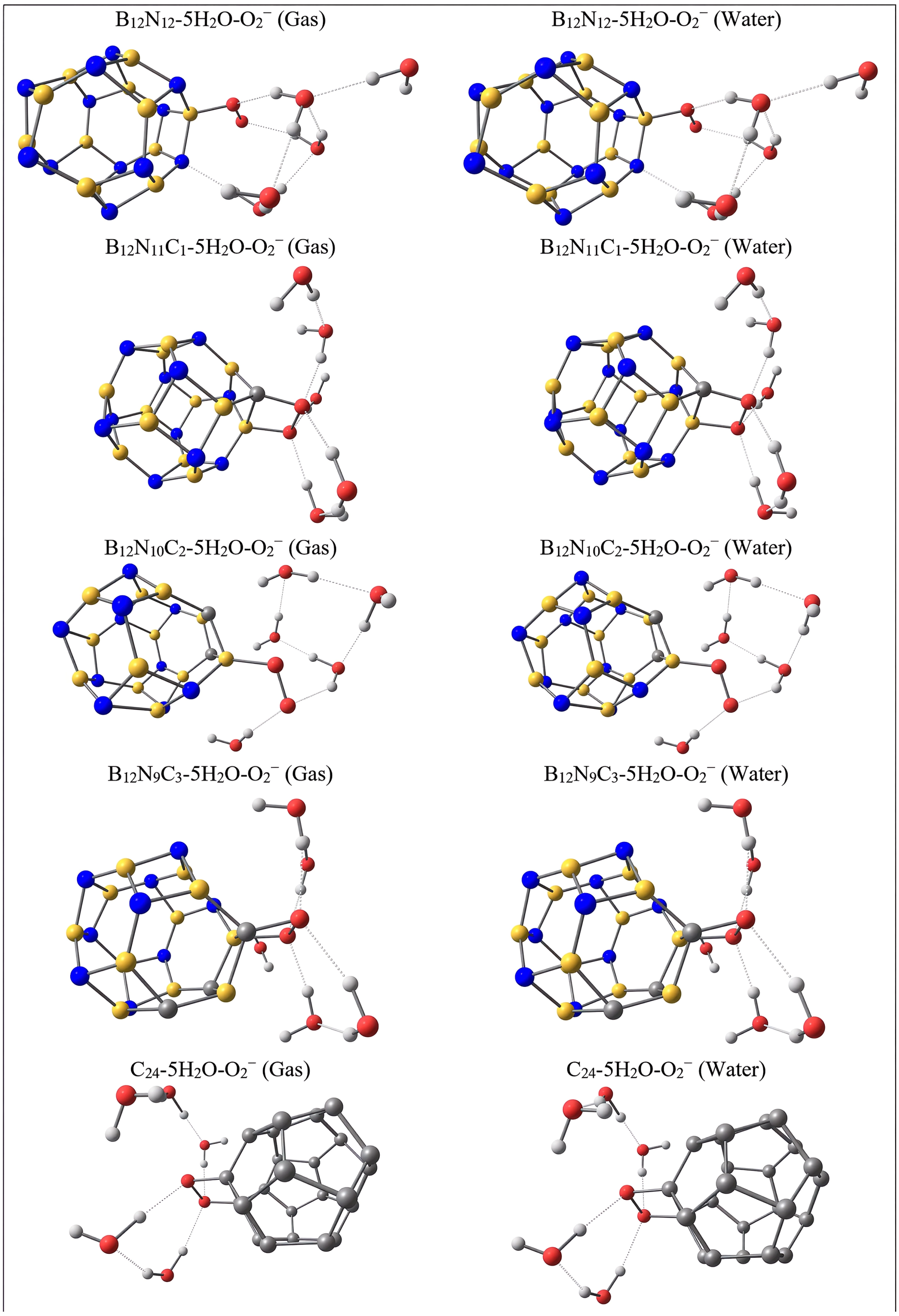

Figure S3: Microhydrated models constructed by placing five water molecules around the O_2^- adsorption site. The $5H_2O$ cluster-continuum model was used for the primary local hydration comparison.

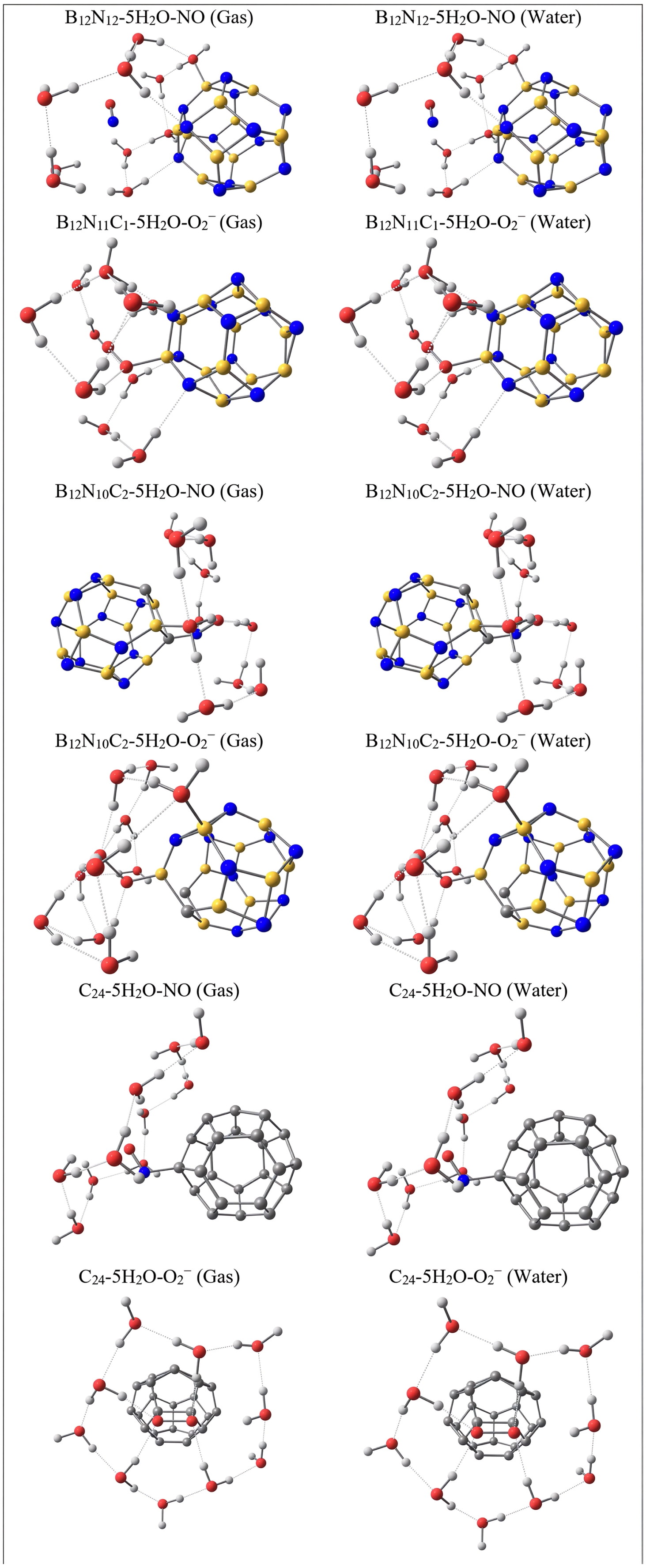

Figure S4: Microhydrated models constructed by placing ten water molecules around the NO/O_2^- adsorption site.

Table S15. Primary $5H_2O$/PCM frozen-hydration vertical adsorption-energy set compared with the continuum-only aqueous PCM values.

BNF	Adsorbate	Implicit PCM / eV	$5H_2O$ + PCM / eV	Explicit - implicit / eV
$B_{12}N_{12}$	NO	-0.130	-0.249	-0.119
$B_{12}N_{11}C_1$	NO	-1.960	-2.610	-0.650
$B_{12}N_{10}C_2$	NO	-1.090	-2.897	-1.807

BNF	Adsorbate	Implicit PCM / eV	$5H_2O$ + PCM / eV	Explicit - implicit / eV
$B_{12}N_9C_3$	NO	-2.060	-3.071	-1.011
C_{24}	NO	-0.890	-2.657	-1.767
$B_{12}N_{12}$	O_2^-	-1.980	-2.617	-0.637
$B_{12}N_{11}C_1$	O_2^-	-3.320	-3.934	-0.614
$B_{12}N_{10}C_2$	O_2^-	-2.480	-3.034	-0.554
$B_{12}N_9C_3$	O_2^-	-2.620	-4.109	-1.489
C_{24}	O_2^-	-2.390	-4.191	-1.801

Favorable results were obtained for all ten $5H_2O$/PCM cases compared to their continuum-only PCM counterparts. For O_2^-, the explicit water correction ranges from -0.554 to -1.801 eV, with an average of -1.019 eV. For NO, the sign of the correction is consistent and stabilizing, but the magnitude varies by BNF. The mean correction for the five fullerenes is -1.071 eV, ranging from -0.119 eV for $B_{12}N_{12}$ to -1.807 eV for $B_{12}N_{10}C_2$. The small change obtained for $B_{12}N_{12}$–NO contrasts with the larger changes observed for some C-doped BNFs, demonstrating a strong BNF dependence.

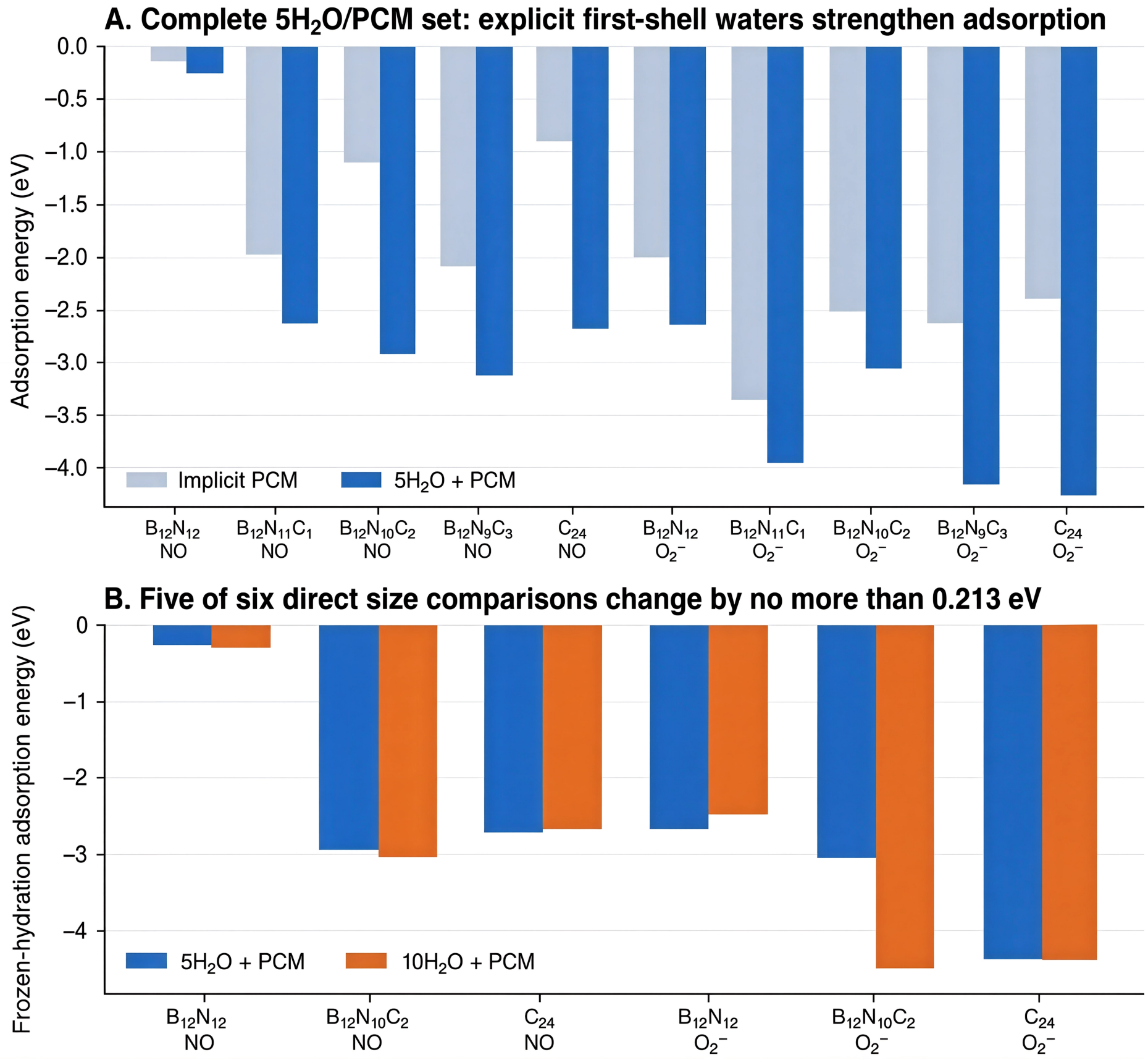

Figure S5: **(A)** Continuum-only aqueous PCM adsorption energies from manuscript Tables 2 and 3 and $5H_2O$/PCM frozen-hydration adsorption energies for the complete ten-case primary set. **(B)** All six direct $5H_2O$-to-$10H_2O$ PCM comparisons. More negative values indicate stronger adsorption.

Table S16. Converged $10H_2O$/PCM sensitivity energies. All six rows now have a direct $5H_2O$/PCM match.

BNF	Adsorbate	$10H_2O$ + PCM / eV	$5H_2O$ + PCM / eV	$10H_2O$ - $5H_2O$ / eV
$B_{12}N_{12}$	NO	-0.273	-0.249	-0.024
$B_{12}N_{10}C_2$	NO	-2.950	-2.897	-0.053
C_{24}	NO	-2.592	-2.657	+0.066
$B_{12}N_{12}$	O_2^-	-2.404	-2.617	+0.213
$B_{12}N_{10}C_2$	O_2^-	-4.344	-3.034	-1.309
C_{24}	O_2^-	-4.215	-4.191	-0.024

Median change from five to ten waters over the six matched pairs is -0.024 eV. $B_{12}N_{12}$-NO, $B_{12}N_{10}C_2$-NO, C_{24}-NO, $B_{12}N_{12}$-O_2^-, and C_{24}-O_2^- all remain within 0.213 eV of their $5H_2O$ values. Together, these results indicate that the qualitative microhydration correction is already represented by the five-water model for these systems. $B_{12}N_{10}C_2$-O_2^- is the only strong, size-sensitive case, becoming 1.309 eV more favorable with ten waters. Given this, the most plausible interpretation is a particularly favorable, larger hydrogen-bond/polarization network around the charged adsorbate. This point should be described as configuration- and size-sensitive rather than as evidence that every system requires ten waters.

Calculations of the $5H_2O$ cluster without PCM help separate local and long-range solvent effects. For O_2^-, the unscreened $5H_2O$ cluster interactions are exceptionally strong (-5.562 to -7.593 eV). The addition of the PCM continuum weakens these interactions by 2.660 to 3.402 eV (mean 2.964 eV); however, the resulting $5H_2O$/PCM values remain more favorable than those of the continuum-only PCM. Discrete hydrogen bonding/local polarization and bulk dielectric screening act in opposite directions. The explicit first shell stabilizes the adsorbed state locally, and the continuum screens the very large electrostatic interaction of the isolated charged cluster. O_2^- adsorption is more favorable in each $5H_2O$/PCM BNF than in the reported PCM-only

BNF, with shifts ranging from 0.554 to 1.801 eV (mean 1.019 eV). For $B_{12}N_{12}$, $B_{12}N_{11}C_1$, and $B_{12}N_{10}C_2$, the effect is smaller (0.554–0.637 eV), while for $B_{12}N_9C_3$ and C_{24}, it is larger (1.489 and 1.801 eV), revealing a pronounced BNF dependence. This is consistent with **directional hydrogen bonding and the local polarization of the anionic adsorbate**, which cannot be completely represented by a continuum. The $10H_2O$ subset corroborates this interpretation, offering an important qualification. The energies of $B_{12}N_{12}$-O_2^- and C_{24}-O_2^- vary by only +0.213 and -0.024 eV, respectively, when the cluster grows from five to ten water molecules. $B_{12}N_{10}C_2$-O_2^- varies by -1.309 eV. The latter system is therefore sensitive to the extended water arrangement, and its exact magnitude should not be generalized. Nevertheless, its direction is consistent with, rather than contradictory to, the first-shell stabilization observed at $5H_2O$.

NO's response is heterogeneous because it is neutral, and its hydration depends strongly on the local BNF composition and adsorption geometry. The correction is small for $B_{12}N_{12}$, moderate for $B_{12}N_{11}C_1$, and greater than 1.00 eV for $B_{12}N_9C_3$, $B_{12}N_{10}C_2$, and C_{24}. Comparisons of the available $10H_2O$ for $B_{12}N_{12}$-NO, $B_{12}N_{10}C_2$-NO, and C_{24}-NO differ from the $5H_2O$ by only -0.024, -0.053, and +0.066 eV, respectively. These results support the idea that the difference among BNFs is a local chemical effect rather than an artifact of using five waters instead of ten and the cross-size agreement supports **weak NO adsorption on pristine $B_{12}N_{12}$ under aqueous conditions**. **Explicit hydration strengthens adsorption relative to the reported implicit PCM reference for both adsorbates**. The mean shifts are comparable for NO and O_2^-, but the BNF dependence differs. The bulk PCM also screens the extreme binding found in unsolvated, charged $5H_2O$ clusters. Five water molecules capture most of the first-shell effects, while ten water molecules expose the $B_{12}N_{10}C_2$-O_2^- sensitivity.

Section 8. Sensitivity of adsorption and electronic descriptors

To evaluate the sensitivity of the aqueous-phase results to the implicit-solvent description, additional vertical calculations were performed using PCM and SMD water models at the same ωB97X-D/6-311++G(d,p) level. These calculations were performed on the same optimized aqueous geometries used in the main manuscript. This protocol isolates the effect of the continuum-solvent model without introducing changes to the geometry, functional, or basis set. Vertical single-point calculations with NBO and polarizability analysis were performed for each BNF-radical complex. We also analyzed TD-DFT calculations to compare the predicted optical transitions in the 300–600 nm region. The values in the tables are reported as PCM/SMD. The PCM values correspond to the optimized PCM aqueous values reported in the main manuscript, and the SMD values correspond to vertical sensitivity estimates. Thus, SMD values should be used to evaluate model dependence rather than to replace the optimized adsorption energies reported in the main manuscript.

Table S17. Solvent-model sensitivity of aqueous descriptors for BNF-NO complexes. Values are reported as PCM / SMD.

Quantum descriptor	$B_{12}N_{12}$–NO	$B_{12}N_{11}C_1$–NO	$B_{12}N_{10}C_2$–NO	$B_{12}N_9C_3$–NO
E_{ad} / eV	-0.13 / -0.09	-1.96 / -2.15	-1.09 / -1.27	-2.06 / -2.25
E_{ad} SMD–PCM / eV	+0.04	-0.19	-0.18	-0.20
Bond length / Å	2.35 (B–N) / 2.35 (B–N)	1.44 (C–N) / 1.44 (C–N)	1.44 (C–N) / 1.44 (C–N)	1.42 (C–N) / 1.42 (C–N)
q_{ads} NBO / e	+0.136 / +0.132	-0.188 / -0.215	-0.077 / -0.100	-0.248 / -0.278
HL_{gap} / eV	8.35 / 8.30	8.30 / 8.34	6.84 / 6.99	7.36 / 7.66
DM / Debye	1.82 / 1.71	3.99 / 4.33	3.89 / 4.39	5.06 / 5.59
α / a.u.	241.05 / 259.42	250.45 / 271.22	275.45 / 296.20	275.13 / 301.79
λ strongest 300–600 nm / nm	— / —	— / —	457.57 / 463.70	302.12 / 419.26
f strongest 300–600 nm	— / —	— / —	0.0187 / 0.0199	0.0095 / 0.0033

Table S18. Solvent-model sensitivity of aqueous descriptors for BNF-O_2^- complexes. Values are reported as PCM / SMD.

Quantum descriptor	$B_{12}N_{12}$–O_2^-	$B_{12}N_{11}C_1$–O_2^-	$B_{12}N_{10}C_2$–O_2^-	$B_{12}N_9C_3$–O_2^-
E_{ad} / eV	-1.98 / -1.48	-3.32 / -2.94	-2.48 / -2.07	-2.62 / -2.25
E_{ad} SMD–PCM / eV	+0.50	+0.38	+0.41	+0.37
Bond length / Å	1.51 (B–O) / 1.51 (B–O)	1.43 (C–O); 1.48 (B–O) / 1.43 (C–O); 1.48 (B–O)	1.49 (B–O) / 1.49 (B–O)	1.43 (C–O); 1.51 (B–O) / 1.43 (C–O); 1.51 (B–O)
q_{ads} NBO / e	-0.783 / -0.788	-0.836 / -0.867	-0.776 / -0.573	-0.829 / -0.860
HL_{gap} / eV	9.12 / 9.09	8.45 / 8.61	7.72 / 7.61	7.15 / 7.30
DM / Debye	8.48 / 9.13	8.27 / 9.08	9.29 / 10.11	7.50 / 8.43
α / a.u.	250.57 / 268.58	258.89 / 278.37	274.28 / 297.63	291.80 / 317.90
λ strongest 300–600 nm / nm	— / 317.74	330.25 / 322.01	402.91 / 428.66	331.17 / 318.90
f strongest 300–600 nm	— / 0.0028	0.0046 / 0.0049	0.0129 / 0.0154	0.0612 / 0.0700

Table S19. TD-DFT solvent-model sensitivity for the strongest transitions in the 300-600 nm region.

System	Adsorbate	PCM states 300–600	SMD states 300–600	λ strongest PCM / nm	λ strongest SMD / nm	$\Delta\lambda$ / nm	f PCM	f SMD	Delta f
$B_{12}N_{12}$	NO	0	0	—	—	—	—	—	—
$B_{12}N_{12}$	O_2^-	0	1	—	317.74	—	—	0.0028	—
$B_{12}N_{11}C_1$	NO	0	0	—	—	—	—	—	—
$B_{12}N_{11}C_1$	O_2^-	1	1	330.25	322.01	-8.24	0.0046	0.0049	0.0003
$B_{12}N_{10}C_2$	NO	13	13	457.57	463.70	+6.13	0.0187	0.0199	+0.0012
$B_{12}N_{10}C_2$	O_2^-	5	6	402.91	428.66	+25.75	0.0129	0.0154	+0.0025
$B_{12}N_9C_3$	NO	5	3	302.12	419.26	+117.14	0.0095	0.0033	-0.0062
$B_{12}N_9C_3$	O_2^-	5	5	331.17	318.90	-12.27	0.0612	0.0700	+0.0088

The PCM/SMD comparison shows that qualitative charge-transfer and radical-anchoring trends are not only governed by the choice of implicit solvent model. For NO adsorption, the change in adsorption energy is low to moderate for C-doped BNFs. $B_{12}N_{12}$-NO remains weakly adsorbed under both models, while $B_{12}N_{11}C_1$-, $B_{12}N_{10}C_2$-, and $B_{12}N_9C_3$-NO are more stable than pristine $B_{12}N_{12}$-NO. These results support the conclusion that C-doping enhances NO anchoring. The largest solvent-model effect is observed in anionic O_2^- complexes. In all BNF-O_2^- systems, SMD makes the vertical adsorption energies less exothermic than PCM by approximately 0.37–0.50 eV. This behavior is expected since **anionic adsorbates and charge-separated structures are sensitive to the continuum solvation model's details**. Notably, all O_2^- complexes remain attractively bound in both PCM and SMD. Thus, the qualitative radical-trapping conclusion is preserved, even though the absolute aqueous adsorption energies are model-dependent.

NBO charge-transfer descriptors are less sensitive than adsorption energies for most systems. The direction of charge redistribution remains consistent for pristine and C-doped BNFs, which supports the mechanistic interpretation that radical anchoring is associated with local charge redistribution at the B/C/N active sites. The most pronounced variation in the quadrupoles occurs in the $B_{12}N_{10}C_2$-O_2^- complex, demonstrating that charge localization in some anionic complexes is more sensitive to the solvent model. HL_{gap} values are generally robust at the qualitative level. C-doped systems remain distinct from pristine $B_{12}N_{12}$, and the solvent model does not invert the main electronic trends. Optical properties are more sensitive. Several TD-DFT transitions in the 300–600 nm region persist under both models, but the position and oscillator strength of individual peaks can shift considerably. The most notable example is $B_{12}N_9C_3$-NO, for which the strongest transition in the 300–600 nm region shifts from 302.12 nm in PCM to 419.26 nm in SMD. Therefore, predicted **UV-vis bands should be interpreted as solvent-dependent optical fingerprints rather than single, universal sensing bands**. C-doped complexes are more robust due to their support by multiple independent indicators. These indicators include short bond distances, NBO charge redistribution, and topological descriptors, which are reported elsewhere in the Supporting Information.

Section 9. Finite-temperature ADMP molecular dynamics

Finite-temperature structural behavior was examined using density-functional-theory-based atomic density matrix propagation (ADMP) trajectories in Gaussian 16 at the ωB97X-D/6-311++G(d,p) level. The trajectories were initiated from the corresponding optimized structures while retaining the charge and spin multiplicity used in the static calculations. Nuclear motion was propagated with a 0.5 fs time step using a nominal 300 K thermostat. Radical-bound systems were examined in the gas phase and with PCM water. The isolated $B_{12}N_{12}$, $B_{12}N_{11}C_1$, $B_{12}N_{10}C_2$, and $B_{12}N_9C_3$ cages were analyzed separately to distinguish intrinsic framework stability from adsorbate-induced chemical reconstruction.

Framework deformation was quantified using the Kabsch-aligned root-mean-square deviation (RMSD) of the 24 cage atoms relative to the first ADMP frame. For adsorbate-containing systems, the minimum adsorbate–cage distance and the internal N–O or O–O distance were monitored together with defect-localized cage contacts when required. Cage-only RMSD was used instead of whole-system RMSD because translational separation of a desorbing fragment can otherwise dominate the metric without implying disruption of the fullerene framework. The analyzed trajectory length for every system is reported in Table S20. Accordingly, events observed within these windows are treated as direct dynamical evidence, whereas the absence of an event is not converted into a long-time residence time or kinetic rate. PCM trajectories represent implicit aqueous solvation and therefore do not include explicit solvent collisions, hydrogen-bond rearrangement, or proton-transfer events.

9.1 Intrinsic stability of the isolated cages

All four isolated cages retained a closed fullerene topology over the analyzed 300 K trajectories (Figure S6). The current observation windows extend to 4.044 ps for $B_{12}N_{12}$, 2.769 ps for $B_{12}N_{11}C_1$, 3.423 ps for $B_{12}N_{10}C_2$, and 2.411 ps for $B_{12}N_9C_3$. The maximum Kabsch-aligned cage RMSD was 0.084 Å, 0.088 Å, 0.085 Å, and 0.107 Å, respectively. The maximum elongation of an initial cage bond remained below 0.18 Å. Thus, the longer finite-temperature windows continue to support intrinsic preservation of the closed cages, and the larger structural changes observed for selected radical-bound systems arise from adsorbate-induced chemistry rather than spontaneous thermal opening of the parent framework.

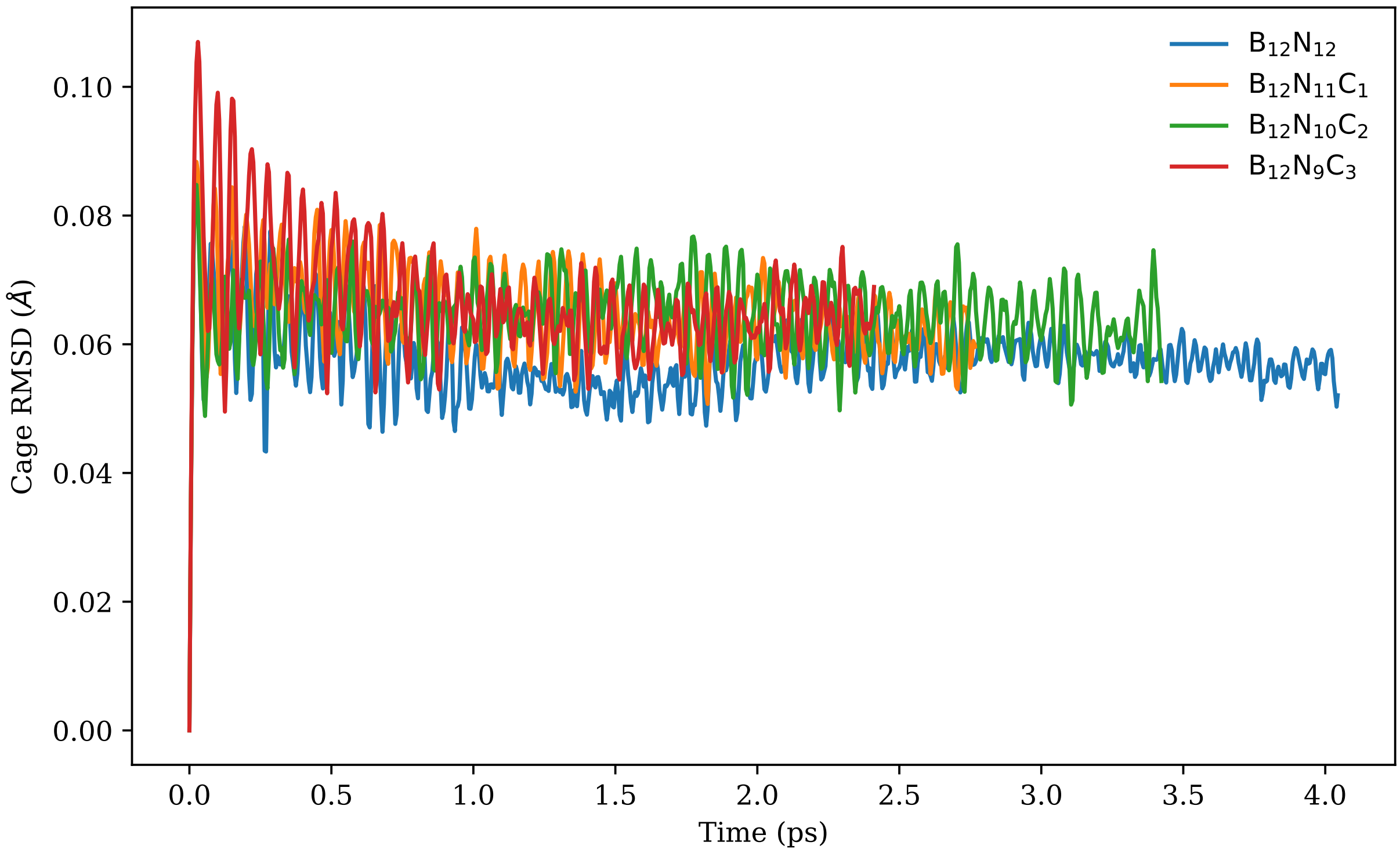

Figure S6: Kabsch-aligned RMSD of the 24 cage atoms for the isolated pristine and C-doped boron nitride fullerenes during 300 K ADMP. The small deviations and absence of sustained RMSD growth indicate preservation of the closed cage topology over the analyzed windows.

9.2 Radical-bound trajectories and relation to the PES analysis

The weakly bound pristine $B_{12}N_{12}$–NO complex exhibits direct NO separation in both media. The minimum NO–cage distance exceeds 3 Å at approximately 0.080 ps in the gas phase and 0.110 ps in PCM water and reaches 18.59 and 13.82 Å, respectively, by the end of the available trajectories (Figure S7). In contrast, the cage-only RMSD remains below 0.10 Å. The event therefore corresponds to molecular desorption rather than cage destabilization and is consistent with the shallow adsorption well and ultralabile first-passage behavior obtained from the static/PES analysis.

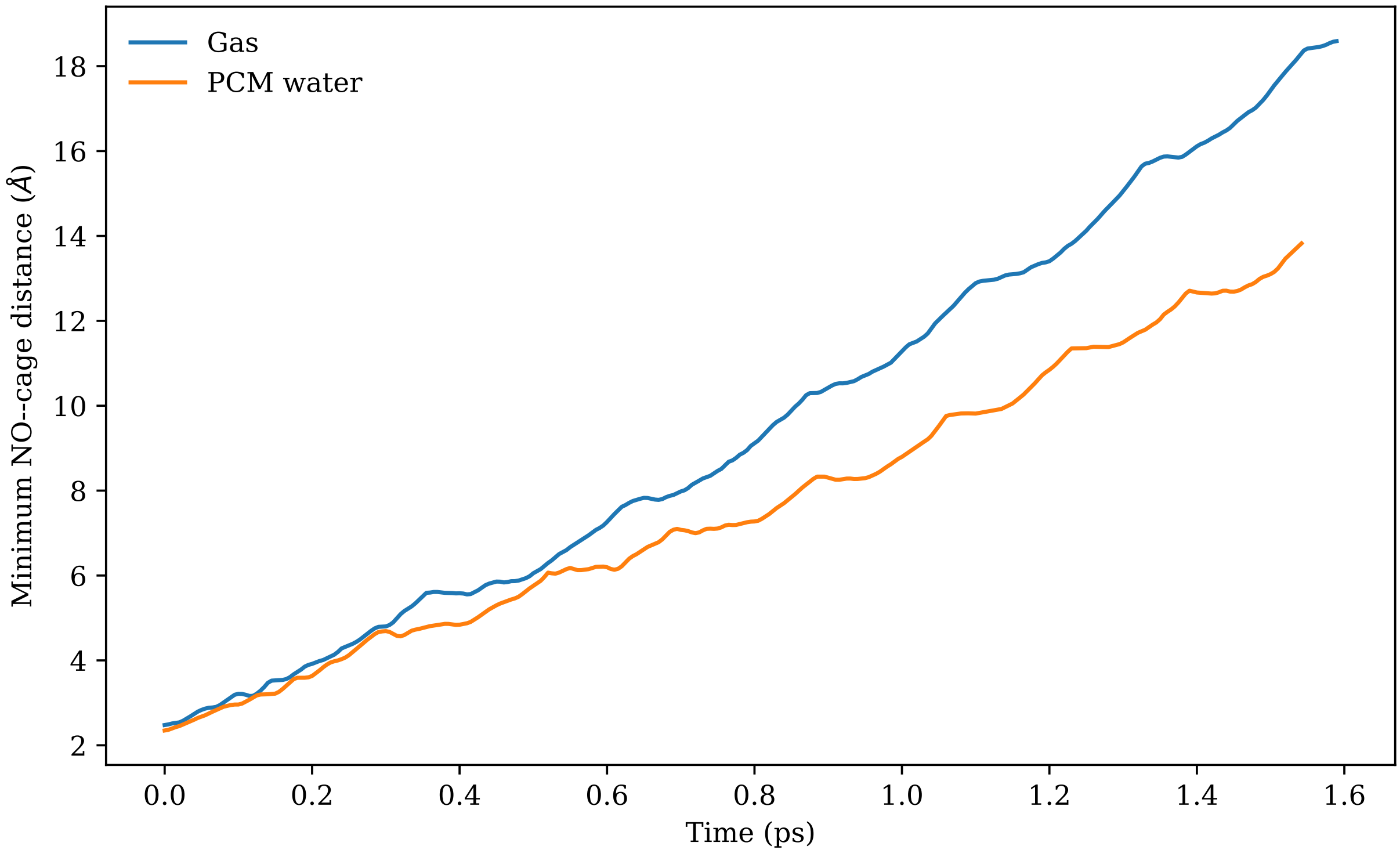

Figure S7: Minimum NO–cage distance for $B_{12}N_{12}$–NO in the gas phase and PCM water. Rapid growth of the separation coordinate provides direct finite-temperature evidence of NO desorption from the pristine cage.

For $B_{12}N_{10}C_2$–O_2^-, the isolated molecular superoxide adsorption motif remains surface-bound in both media through 2.016 ps in the gas phase and 1.854 ps in PCM water. The final O–O distances are 1.300 and 1.339 Å, while the final minimum O_2^-–cage contacts are 1.493 and 1.515 Å, respectively (Figure S8). The maximum cage RMSD remains approximately 0.10 Å. Thus, in the absence of coadsorbed NO, this composition continues to provide the clearest finite-temperature evidence for preservation of molecularly adsorbed O_2^-.

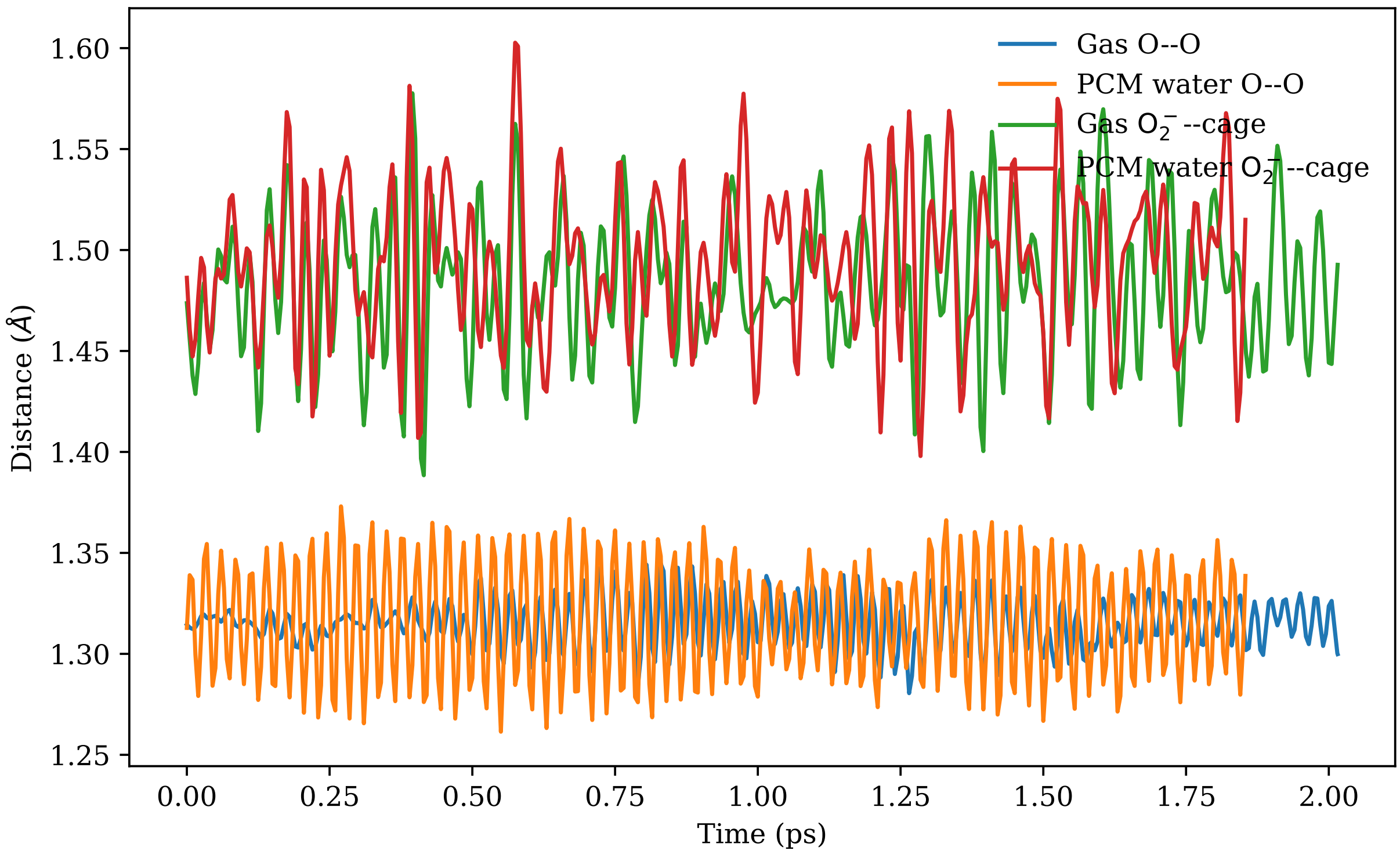

Figure S8: O–O bond length and minimum O_2^-–cage distance for $B_{12}N_{10}C_2$–O_2^- in the gas phase and PCM water. Both coordinates remain within the molecularly adsorbed regime over the analyzed windows.

A different response is observed for $B_{12}N_{11}C_1$–O_2^-. The O–O distance exceeds 2.0 Å after approximately 0.085 ps in the gas phase and 0.095 ps in PCM water and remains in the dissociated regime over the extended trajectories, which now reach 2.953 and 2.733 ps. The final O–O separations are 4.730 and 3.179 Å, respectively. Dissociation is coupled to substantial opening of the local C1–B13 defect contact, which starts at 1.787 and 1.756 Å and reaches maxima of 3.846 and 3.790 Å (Figure S9). The

oxygen atoms remain associated with the defect region, so the trajectory is better described as dissociative/reactive trapping than as molecular superoxide desorption.

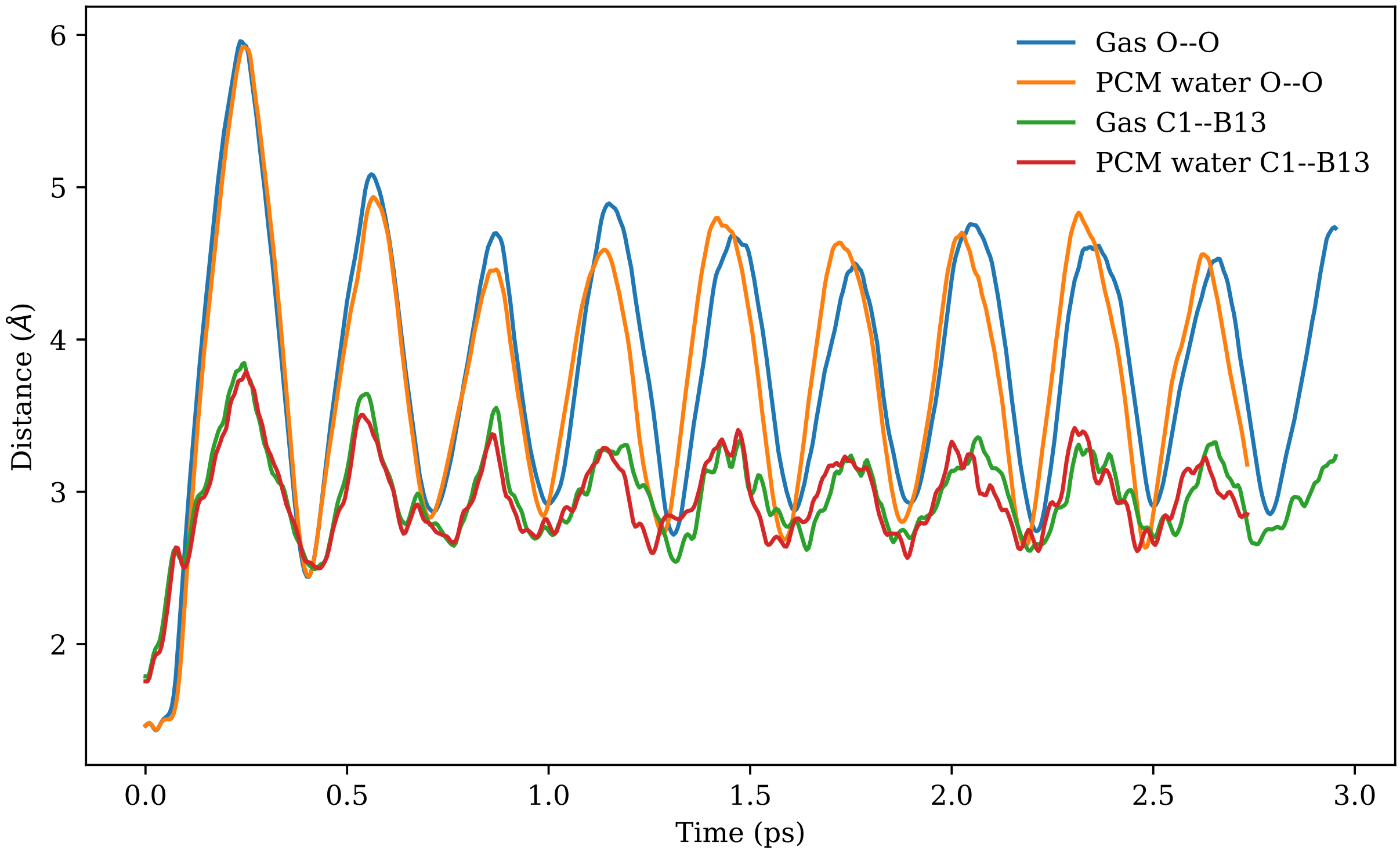

Figure S9: O–O and defect C1–B13 distances for $B_{12}N_{11}C_1–O_2^-$. Rapid O–O elongation is accompanied by local opening of the C-doped cage in both gas and PCM-water trajectories, demonstrating adsorbate-induced dissociative reconstruction.

The longer $B_{12}N_9C_3–O_2^-$ trajectories refine the earlier short-time interpretation. The initial event remains rapid defect-region reconstruction: the B9–C20 contact first exceeds 2.5 Å near 0.09 ps in both media and eventually reaches maxima of 3.698 Å in the gas phase and 3.956 Å in PCM water. However, continued propagation now reveals a second stage. The O–O distance exceeds 2.0 Å at approximately 0.460 ps in the gas phase and 0.470 ps in PCM water, reaching maxima of 5.640 and 6.285 Å, respectively (Figure S10). Thus, the longer trajectories show that early reanchoring/contact switching is followed by O–O dissociation and reactive trapping rather than indefinitely preserving molecular superoxide.

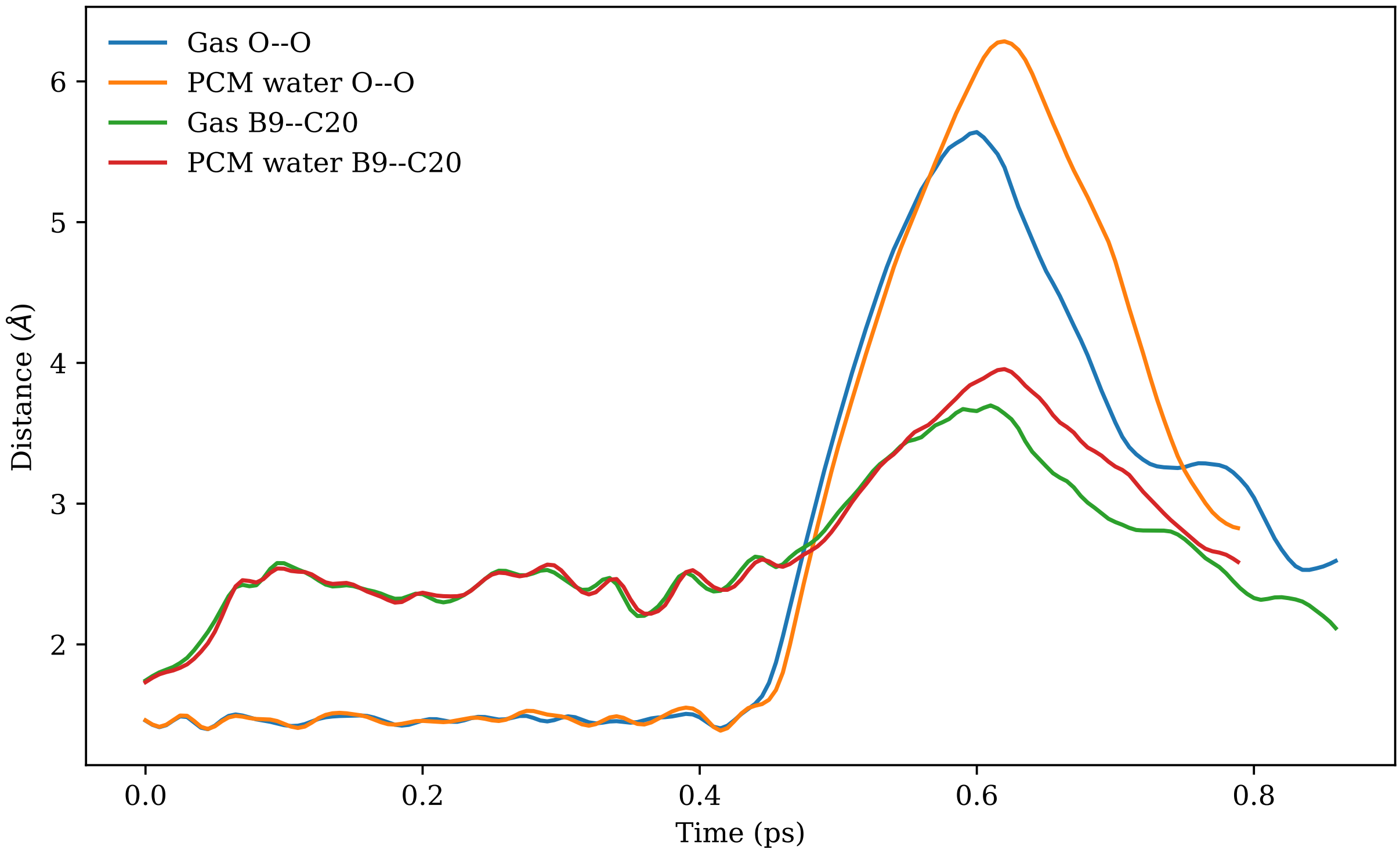

Figure S10: O–O and B9–C20 distances for $B_{12}N_9C_3–O_2^-$ in gas and PCM water. Early defect-localized reconstruction precedes a later O–O dissociation event in both media, revealing a two-stage reactive trapping process over the extended trajectories.

For NO adsorption on $B_{12}N_{10}C_2$, the C–N contact remains short while the adjacent B9–C10 cage bond reaches maxima of

3.125 Å in the gas phase and 3.055 Å in PCM water, compared with initial values of 1.651 and 1.664 Å, respectively. This trajectory therefore continues to represent defect-localized reconstruction with persistent NO binding rather than molecular desorption. By comparison, NO remains adsorbed on $B_{12}N_{11}C_1$ and $B_{12}N_9C_3$ over their available windows without the rapid desorption observed for pristine $B_{12}N_{12}$.

9.3 Co-adsorption dynamics

The extended $B_{12}N_{10}C_2$–NO/O_2^- trajectories reveal a medium-dependent fate (Figure S11). In the gas phase, the original selective-retention picture remains unchanged: NO moves from 3.06 to 10.67 Å from the cage, whereas molecular O_2^- remains surface-bound with an O–O distance of 1.311 Å and a final minimum cage contact of 1.435 Å. In PCM water, NO likewise separates to 11.14 Å; however, after approximately 1.225 ps the O–O coordinate enters a dissociative regime. The O–O distance reaches a maximum of 4.521 Å and ends at 2.201 Å, while the local C20–B21 contact opens from 1.578 to a maximum of 3.821 Å. Both oxygen-derived fragments remain surface-associated. Thus, preferential loss of NO is observed in both media, but preservation of the original molecular superoxide motif is supported only by the gas-phase co-adsorption trajectory over the present observation windows. The PCM-water trajectory instead indicates selective retention through reactive oxygen trapping.

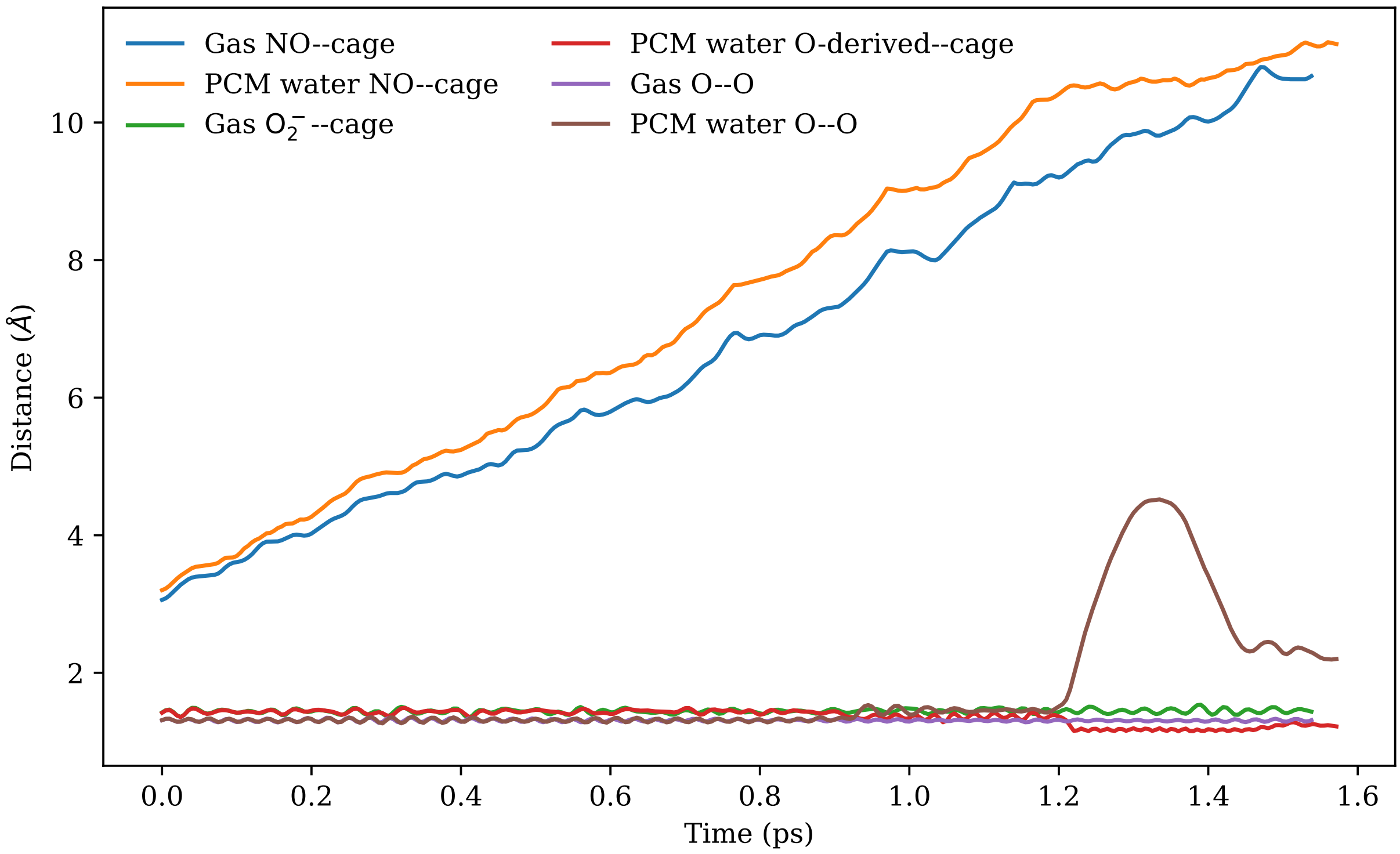

Figure S11: Minimum NO–cage and oxygen-derived–cage distances together with the O–O coordinate during co-adsorption on $B_{12}N_{10}C_2$. In the gas phase, molecular O_2^- remains adsorbed while NO separates. In PCM water, NO also separates, but the O–O bond later enters a dissociative regime accompanied by local cage reconstruction.

The longer gas-phase $B_{12}N_9C_3$ co-adsorption trajectory reaches 0.870 ps. Both NO and O_2^- remain surface-bound, with final minimum cage contacts of 1.380 and 1.530 Å, respectively. The local C10–C20 cage contact nevertheless remains reconstructed: it evolves from 1.638 Å to a maximum of 2.684 Å and is still 2.478 Å in the final sampled frame. Dual retention on this composition is therefore accompanied by persistent defect-region reconstruction rather than preservation of a rigid co-adsorbed geometry. For PCM water, only the longest valid contiguous 0.2255 ps trajectory is considered; both adsorbates remain bound over that shorter window.

The pristine $B_{12}N_{12}$ co-adsorption trajectories initiated from the optimized covalently connected O–N–O–O structure continue to show persistence of the preformed product-like motif. In the gas phase, the O25–N26, N26–O27, O27–O28, and O28–B1 distances evolve from 1.173, 1.375, 1.407, and 1.471 Å to 1.150, 1.420, 1.383, and 1.502 Å, respectively, over 2.698 ps. The corresponding PCM-water trajectory extends to 2.471 ps and ends at 1.161, 1.417, 1.484, and 1.493 Å. These trajectories therefore demonstrate finite-temperature persistence of a preformed surface-bound $ONOO^-$-like adduct, but by themselves do not establish its spontaneous formation.

9.4 Precursor-side spontaneous $ONOO^-$-like coupling on $B_{12}N_{12}$

To distinguish persistence of a preformed product from actual bond formation, an additional gas-phase ADMP trajectory was initiated from the precursor-like endpoint of the reverse IRC, for which NO and surface-bound O_2^- are not initially connected by the product-forming N–O bond. The starting N26–O27 and N26–O28 separations are 2.636 and 3.452 Å, respectively. During the trajectory, a spontaneous alternative N26–O28 coupling event occurs: the N26–O28 separation contracts to a minimum of 1.396 Å at approximately 0.475 ps while the O25–N26 and O27–O28 bonds remain intact, producing a transient covalently connected O25–N26–O28–O27 motif (Figure S12). The corresponding cage-only RMSD remains small, with a maximum of 0.096 Å.

The bond-formation event follows a different atom-to-atom connectivity from the static IRC, which proceeds primarily along N26–O27 formation. In the finite-temperature trajectory, N26–O28 formation defines an alternative oxygen-switching coordinate. The N26-O28 contact subsequently reopens and re-approaches, consistent with recrossing rather than irreversible conversion within the 0.7525 ps observation window. Because the trajectory starts from the precursor side rather than from a preformed O–N–O–O product, it directly shows that surface-associated $ONOO^{-}$-like coupling is dynamically accessible on $B_{12}N_{12}$ through a connectivity distinct from the static N26–O27 pathway.

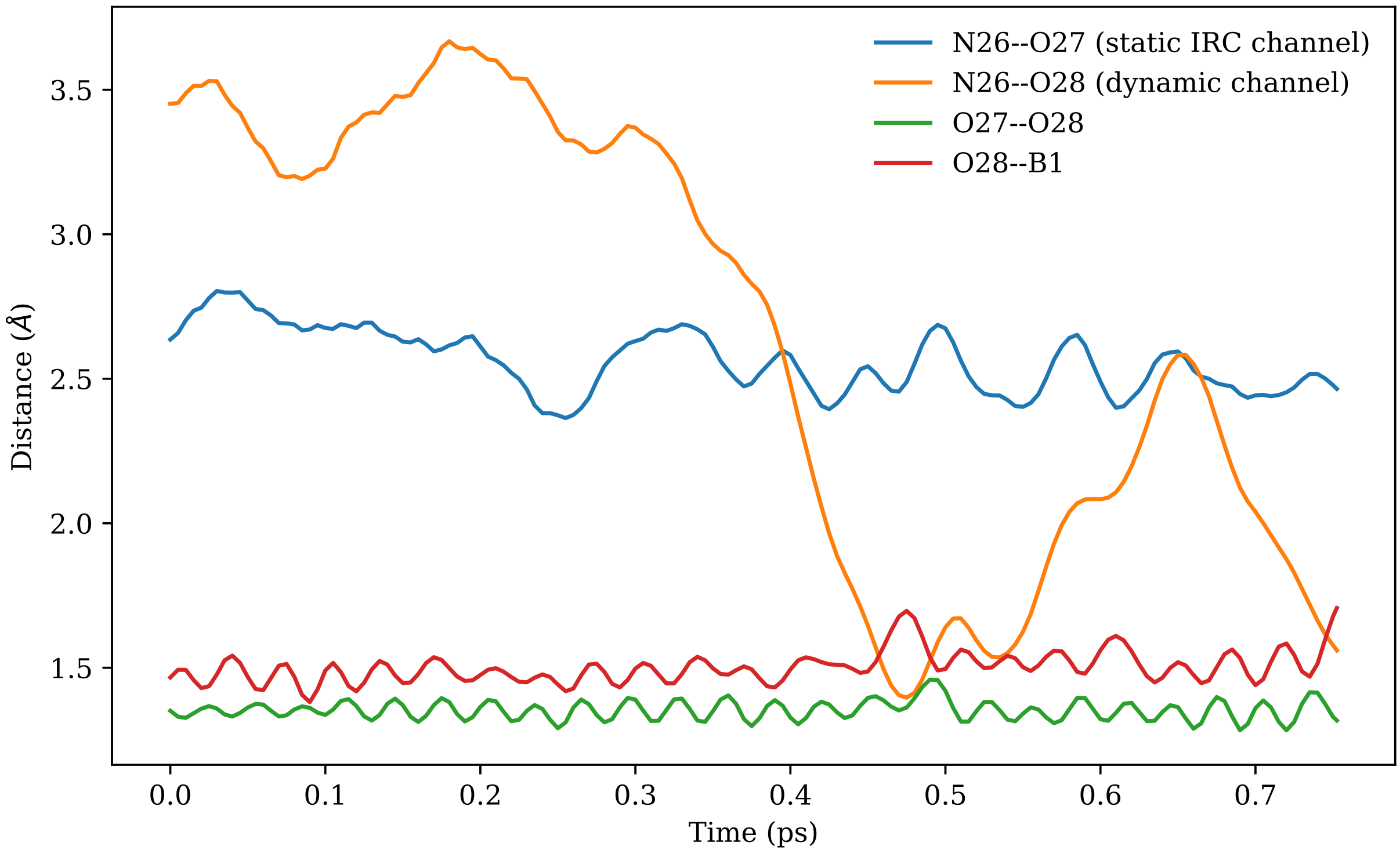

Figure S12: Key bond distances during the precursor-side gas-phase ADMP trajectory for $B_{12}N_{12}$ + NO + O_2^-. The N26–O28 separation contracts from a nonbonded precursor value to approximately 1.40 Å, generating a transient O–N–O–O connectivity distinct from the N26–O27 static IRC channel. Subsequent reopening and re-approach indicate finite-temperature recrossing.

Table S20: Summary of the ADMP trajectories selected for the finite-temperature analysis. Trajectory lengths correspond to the longest valid contiguous observation windows used for interpretation. The RMSD is calculated only for the 24 cage atoms after Kabsch alignment. The dedicated precursor-side ONOO$^-$ formation test is listed separately from the preformed co-adsorption trajectories.

System	Adsorbate	Medium	Time (ps)	Max. cage RMSD (Å)	Observed behavior
$B_{12}N_{10}C_2$	—	Isolated	3.4230	0.085	Closed cage; no substantial framework disruption.
$B_{12}N_{10}C_2$	NO	Gas	2.0645	0.278	NO remains C-bound with local defect-bond opening.
$B_{12}N_{10}C_2$	NO + O_2^-	Gas	1.5380	0.097	Molecular O_2^- remains bound while NO separates.
$B_{12}N_{10}C_2$	NO + O_2^-	PCM water	1.5715	0.388	NO separates; O–O dissociation and local reconstruction develop after ∼1.2 ps.
$B_{12}N_{10}C_2$	NO	PCM water	1.8135	0.263	NO remains C-bound with local defect-bond opening.
$B_{12}N_{10}C_2$	O_2^-	Gas	2.0155	0.100	Molecular O_2^- remains adsorbed.
$B_{12}N_{10}C_2$	O_2^-	PCM water	1.8540	0.101	Molecular O_2^- remains adsorbed.
$B_{12}N_{11}C_1$	—	Isolated	2.7690	0.088	Closed cage; no substantial framework disruption.
$B_{12}N_{11}C_1$	NO	Gas	2.9490	0.098	NO remains adsorbed; cage remains globally intact.
$B_{12}N_{11}C_1$	NO + O_2^-	Gas	1.6775	0.090	Both adsorbates remain surface-bound within the observed window.
$B_{12}N_{11}C_1$	NO + O_2^-	PCM water	1.6085	0.094	Both adsorbates remain surface-bound within the observed window.
$B_{12}N_{11}C_1$	NO	PCM water	2.6985	0.100	NO remains adsorbed; cage remains globally intact.
$B_{12}N_{11}C_1$	O_2^-	Gas	2.9525	0.372	O–O dissociation with defect-localized reactive reconstruction.
$B_{12}N_{11}C_1$	O_2^-	PCM water	2.7330	0.355	O–O dissociation with defect-localized reactive reconstruction.
$B_{12}N_{12}$	—	Isolated	4.0440	0.084	Closed cage; no substantial framework disruption.
$B_{12}N_{12}$	NO	Gas	1.5895	0.072	Rapid NO desorption; cage remains intact.
$B_{12}N_{12}$	NO + O_2^-	Gas	2.6980	0.090	Preformed surface-bound O–N–O–O motif persists.
$B_{12}N_{12}$	NO + O_2^-	PCM water	2.4710	0.088	Preformed surface-bound O–N–O–O motif persists.
$B_{12}N_{12}$	NO	PCM water	1.5420	0.099	Rapid NO desorption; cage remains intact.
$B_{12}N_{12}$	O_2^-	Gas	2.1550	0.076	Molecular O_2^- retained over the observed window.
$B_{12}N_{12}$	O_2^-	PCM water	2.0315	0.079	Molecular O_2^- retained over the observed window.
$B_{12}N_{12}$	precursor NO+O_2^-	Gas	0.7525	0.096	Spontaneous transient ONOO$^-$-like coupling through alternative N26–O28 formation.
$B_{12}N_9C_3$	—	Isolated	2.4110	0.107	Closed cage; no substantial framework disruption.
$B_{12}N_9C_3$	NO	Gas	2.7895	0.103	NO remains adsorbed over the observed window.
$B_{12}N_9C_3$	NO + O_2^-	Gas	0.8700	0.249	Both adsorbates remain bound with persistent defect-region reconstruction.
$B_{12}N_9C_3$	NO + O_2^-	PCM water	0.2255	0.091	Both adsorbates remain bound over the longest valid contiguous window.
$B_{12}N_9C_3$	NO	PCM water	0.8260	0.104	NO remains adsorbed over the observed window.
$B_{12}N_9C_3$	O_2^-	Gas	0.8590	0.380	Early reconstruction followed by O–O dissociation/reactive trapping.

System	Adsorbate	Medium	Time (ps)	Max. cage RMSD (Å)	Observed behavior
$B_{12}N_9C_3$	O_2^-	PCM water	0.7885	0.407	Early reconstruction followed by O–O dissociation/reactive trapping.

Interpretive scope. These trajectories provide finite-temperature structural tests of specific optimized starting states. They distinguish intrinsic cage stability, molecular desorption, molecular retention, adsorbate-induced reconstruction, dissociative trapping, and precursor-side bond formation on the picosecond and sub-picosecond time scales actually sampled. They do not by themselves provide macroscopic lifetimes, competitive association rate constants, or explicit-solvent reaction kinetics. The precursor-side $B_{12}N_{12}$ trajectory demonstrates that an $ONOO^-$-like covalent motif is dynamically accessible from the reactant side. The observed oxygen-switching event is reported here as a finite-temperature channel distinct from the static N26–O27 IRC pathway and is not interpreted as evidence that either connectivity represents a unique finite-temperature mechanism.
